# Supplementary material for: Provision of essential evidence-based interventions during facility-based childbirth: cross-sectional observations of births in northeast Nigeria
Source: BMJ Open. 2020 Oct 23;10(10):e037625. doi: 10.1136/bmjopen-2020-037625 (PMC7590366; doi:10.1136/bmjopen-2020-037625)
Supplement: Supplementary data [file bmjopen-2020-037625supp001.pdf]

Supplementary material: full table of results

	Observation period				
	**1 (Aug 2016)**	**2 (Mar 2017)**	**3 (Aug 2017)**	**4 (Mar 2018)**	**5 (Aug 2018)**
	**Percentage of women (95%CI)**				
**History taking & initial assessment**					
Encourages woman to have a support person	70.8% (50.0-85.5)	57.1% (31.1-79.7)	48.6% (28.0-69.6)	62.6% (45.0-77.4)	77.1% (67.8-84.4)
Asks if have any questions	34.8% (22.7-49.3)	30.0% (12.6-56.2)	16.6% (8.5-29.9)	25.7% (16.5-37.6)	53.4% (37.5-68.6)
Checks client card or asks client age, length of pregnancy and parity	73.2% (53.4-86.7)	82.3% (61.4-93.0)	74.4% (48.2-90.1)	85.3% (74.5-92.1)	85.0% (71.4-92.8)
Checks woman's HIV status	3.0% (1.4-6.3)	24.8% (12.8-42.6)	60.4% (40.9-77.0)	52.3% (32.4-71.6)	67.2% (56.1-76.6)
Asks whether experienced any complications during pregnancy	13.7% (7.9-22.8)	12.8% (7.1-22.1)	9.6% (4.0-20.9)	12.0% (7.2-19.3)	12.6% (6.6-22.7)
If had a previous pregnancy, whether experienced any complications during previous pregnancies	14.6% (9.5-21.8)	17.5% (8.1-34.0)	15.5% (10.3-22.6)	21.8% (14.7-31.0)	21.4% (13.9-31.6)
Health worker washes hands	25.3% (12.6-44.4)	13.4% (5.4-29.4)	11.5% (4.8-25.3)	50.9% (28.8-72.6)	37.2% (23.2-53.8)
Explains procedure to woman before proceeding	69.4% (55.6-80.3)	72.7% (52.3-86.6)	50.0% (27.6-72.4)	60.4% (42.9-75.6)	80.4% (73.3-85.9)
Performs vaginal examination	99.1% (96.6-99.8)	97.5% (94.2-998.9)	98.3% (95.8-99.3)	97.6% (94.2-99.0)	98.5% (97.0-99.3)
If performs vaginal exam, wears high-level disinfectant gloves	93.7% (82.8-97.9)	79.7% (48.6-94.2)	94.9% (84.3-98.5)	43.9% (21.3-69.3)	59.2% (35.9-79.0)
Takes temperature	0.6% (0.2-1.9)	1.1% (0.3-4.1)	3.4% (0.7-14.5)	8.3% (3.1-20.6)	4.7% (1.0-19.0)
Takes blood pressure	24.7% (11.2-46.0)	26.2% (11.0-50.5)	18.3% (9.2-33.0)	50.4% (33.1-67.6)	26.7% (11.7-50.0)
Checks foetal heart rate with fetoscope/Doppler/ultrasound	28.0% (14.0-48.0)	17.3% (7.2-36.0)	9.3% (3.4-23.0)	33.8% (21.0-49.4)	19.9% (11.0-33.5)
**First stage of labour: examination and procedures**					
Partograph used to monitor labour	0.6% (0.1-4.8)	32.3% (14.0-58.3)	29.1% (14.6-49.7)	37.1% (26.6-49.0)	15.4% (7.7-28.6)
Washes hands with soap & water or uses antiseptic prior to any examination of woman	37.2% (19.9-58.7)	21.7% (11.3-37.6)	14.3% (5.9-30.7)	57.2% (32.8-78.6)	53.7% (40.3-66.6)

Wears high-level disinfected or surgical gloves	94.2% (80.1-98.5)	88.9% (71.6-96.2)	94.2% (82.5-98.2)	51.6% (26.7-75.7)	65.2% (35.3-86.5)
Median number of vaginal examinations	2 (1-2)	1 (1-2)	2 (1-2)	1 (1-2)	1 (1-2)
Augments labour with oxytocin	14.5% (7.5-26.3)	12.6% (6.5-23.2)	24.9% (17.2-34.5)	18.0% (7.5-37.2)	15.4% (5.9-34.5)
Performs artificial rupture of membranes	7.6% (3.9-14.0)	9.6% (4.3-20.2)	9.0% (3.3-22.5)	3.5% (1.5-7.9)	1.8% (0.4-6.7)
A support person present at some point during labour	69.2% (47.3-84.9)	70.2% (46.8-86.3)	67.7% (46.1-83.7)	70.7% (54.5-82.9)	78.9% (57.8-91.0)
At least once explains what will happen in labour	46.5% (33.2-60.3)	55.1% (35.5-73.2)	33.9% (17.5-55.3)	42.1% (30.5-54.6)	67.0% (57.2-75.5)
At least encouraged to consume fluids/foods during labour	93.6% (90.4-95.8)	79.3% (66.5-88.1)	87.3% (82.4-91.0)	91.9% (84.7-95.8)	92.1% (78.7-97.3)
Drapes woman	28.5% (12.7-52.1)	59.1% (43.3-73.2)	67.7% (45.5-84.1)	53.7% (26.8-78.7)	80.2% (56.9-92.5)
At least once encourages woman to ambulate and assume different positions in labour	69.8% (50.5-83.9)	60.1% (42.8-75.2)	65.6% (41.2-83.9)	79.9% (63.4-90.1)	85.9% (71.7-93.6)
**First stage of labour: equipment and supplies laid out in preparation for delivery**					
At least two cloths/blankets	91.9% (81.2-96.7)	90.4% (82.7-94.9)	88.9% (58.0-97.9)	85.2% (67.5-94.1)	93.8% (86.6-97.3)
Disposable cord ties or clamps	96.5% (87.1-99.1)	97.0% (93.1-98.7)	98.4% (87.9-99.8)	99.3% (97.4-99.8)	99.6% (97.5-99.9)
Sterile scissors or blade	98.8% (94.9-99.8)	99.0% (96.6-99.7)	100%	98.9% (96.8-99.7)	98.7% (95.5-99.6)
Suction bulb	93.0% (87.3-96.3)	82.3% (71.0-89.9)	84.1% (71.4-91.8)	75.6% (59.1-87.0)	76.7% (44.6-93.0)
Bag & face mask (size 0 or 1)	14.0% (4.7-35.0)	46.0% (26.9-66.2)	50.3% (29.0-71.4)	43.5% (30.4-57.6)	41.0% (17.3-69.7)
**Second and third stage of labour**					
More than one HCW assist with delivery	60.1% (40.4-77.0)	51.4% (36.6-66.0)	59.8% (48.9-69.9)	62.1% (45.1-76.6)	48.7% (36.7-60.8)
A support person for mother present	42.3% (26.2-60.3)	34.2% (14.2-62.0)	20.5% (9.2-39.6)	34.0% (20.3-51.0)	49.3% (37.0-61.7)
Performs episiotomy	1.5% (0.4-5.4)	0.8% (0.2-4.4)	0.6% (0.1-2.4)	2.4% (1.0-5.8)	0.3% (0-2.6)
Mother gave birth in lithotomy position	97.9% (94.3-99.3)	98.9% (96.2-99.7)	91.9% (78.1-97.3)	95.6% (84.8-98.8)	98.5% (96.4-99.4)

As baby's head is delivered, supports perineum	97.3% (94.7-98.7)	94.7% (91.6-96.7)	84.0% (73.5-90.9)	93.9% (89.7-96.4)	94.1% (87.3-97.4)
Checks for another baby prior to giving the uterotonic	56.8% (31.7-78.9)	50.8% (33.6-67.9)	52.8% (37.9-67.2)	81.2% (59.5-92.7)	83.0% (67.9-91.9)
Received uterotonic	97.3% (95.6-98.4)	95.0% (92.7-96.6)	97.5% (95.4-98.6)	97.1% (92.0-99.0)	93.8% (69.2-99.0)
If uterotonic received, timing of administration					
At delivery	0.3% (0-2.4)	0.3% (0-2.7)	0.3% (0.1-1.3)	1.8% (0.6-5.0)	0
Within 1 minute of delivery	23.0% (9.4-46.4)	8.8% (2.7-25.0)	10.9% (5.8-19.5)	11.1% (4.9-23.1)	16.6% (9.0-28.6)
Within 3 minutes of delivery	28.5% (22.7-35.1)	30.7% (19.7-44.5)	26.1% (18.4-35.6)	37.0% (29.4-45.4)	51.3% (42.1-60.4)
More than 3 minutes	47.6% (31.6-64.1)	60.2% (41.1-76.7)	62.5% (54.0-70.3)	50.1% (37.9-62.4)	32.2% (25.3-39.9)
Applies traction to the cord while applying suprapubic counter traction	87.3% (80.8-91.8)	81.9% (61.9-92.7)	80.6% (70.5-87.9)	85.3% (67.3-94.3)	91.2% (71.7-97.7)
If skilled (doctor, nurse, midwife) birth assistant, applies traction to the cord while applying suprapubic counter traction	100%	95.4% (63.0-99.6)	88.7% (81.9-93.2)	82.4% (77.4-86.4)	90.5% (48.1-99.0)
If CHEW birth assistant, applies traction to the cord while applying suprapubic counter traction	87.6% (78.8-93.0)	80.9% (58.9-92.6)	81.9% (67.4-90.8)	85.7% (54.9-96.7)	88.9% (66.8-97.0)
If unskilled birth assistant, applies traction to the cord while applying suprapubic counter traction	86.1% (78.1-91.5)	77.8% (57.5-90.0)	77.0% (63.8-86.4)	85.2% (65.2-94.7)	93.0% (77.1-98.1)
Performs uterine massage immediately following the delivery of the placenta	73.7% (63.7-81.7)	78.6% (60.7-89.8)	63.8% (52.6-73.6)	79.0% (63.6-89.0)	77.4% (63.4-87.2)
Assesses completeness of placenta and membranes	70.7% (52.8-83.9)	25.8% (16.3-38.3)	32.9% (26.4-40.1)	53.8% (29.8-76.2)	61.6% (36.8-81.5)
Assesses for perineal and vaginal lacerations	90.2% (80.5-95.4)	89.4% (78.5-95.2)	75.0% (47.1-91.0)	90.5% (69.0-97.6)	88.0% (66.8-96.4)
**Immediate newborn and postpartum care**					
Immediately dries newborn with towel	89.3% (67.8-97.1)	90.5% (73.9-96.9)	93.9% (84.7-97.7)	98.7% (94.5-99.7)	99.4% (95.6-99.9)
Places baby on mother's abdomen "skin-to-skin"	68.1% (30.5-91.2)	49.0% (26.4-72.0)	66.9% (41.6-85.1)	92.8% (85.0-96.7)	92.3% (76.8-97.8)
Delayed bathing until at least 1 hour after birth	94.3% (87.6-97.5)	96.7% (93.4-98.4)	97.9% (95.6-99.0)	98.1% (96.9-99.0)	96.5% (88.9-99.0)

Ties or clamps cord when pulsations stop or by 2-3 minutes after birth	92.6% (81.6-97.3)	92.8% (82.7-97.2)	91.4% (87.3-94.3)	92.8% (85.4-96.6)	92.7% (76.6-98.0)
Cuts cord with clean blade or scissors	99.7% (97.8-100)	99.7% (97.9-100)	98.8% (95.2-99.7)	99.7% (97.8-100)	99.4% (97.3-99.9)
Administers chlorhexidine to the newborn cord	Not measured	73.7% (46.0-90.2)	81.9% (54.6-94.5)	95.0% (88.8-97.8)	91.1% (78.4-96.6)
Breastfeeding initiated within the first hour	67.5% (41.8-85.7)	27.6% (13.1-49.2)	33.7% (18.1-54.0)	60.6% (39.9-78.2)	55.3% (38.0-71.4)
Check's baby's temperature within 15 minutes after birth	2.7% (0.4-15.2)	0%	0.6% (0.1-5.7)	1.9% (0.8-4.3)	5.4% (1.2-22.0)
Takes mother's vital signs 15 minutes after birth	0.3% (0-2.8)	1.8% (0.5-5.9)	1.8% (0.4-7.3)	6.2% (2.4-14.7)	4.5% (0.9-19.1)
Weighs the baby	Not measured	86.3% (57.2-96.7)	85.6% (63.1-95.4)	96.3% (89.8-98.7)	88.5% (45.4-98.6)
Mother and newborn kept in same room after delivery (rooming in)	98.3% (96.1-99.3)	96.7% (88.3-99.1)	95.7% (92.8-97.5)	98.9% (96.8-99.7)	99.7% (98.4-98.8)
Baby kept skin-to-skin with mother for first hour after birth	68.8% (40.1-87.9)	39.5% (20.0-63.0)	50.3% (29.4-71.1)	72.3% (59.6-82.3)	73.2% (49.0-88.6)
Provides tetracycline ointment prophylaxis	0%	0.3% (0-2.9)	0.9% (0.2-4.6)	10.4% (4.4-22.4)	4.8% (1.5-13.9)
Administers Vitamin K to newborn	0%	0.3% (0-2.1)	0%	0%	0%
Administers chlorhexidine to the newborn cord	0%	73.7% (46.2-90.1)	81.9% (54.8-94.4)	95.0% (88.8-97.8)	91.1% (78.5-96.6)
If mother identified as HIV positive, administers ARV to newborn	100%	75.0% (20.5-97.2)	0%	0%	25.0% (2.3-82.5)
Administers antibiotics to mother	1.0% (0.3-3.6)	1.2% (0.5-2.7)	0%	1.8% (0.9-3.5)	6.0% (1.1-27.0)
