# Supplementary material for: Provision of essential evidence-based interventions during facility-based childbirth: cross-sectional observations of births in northeast Nigeria
Source: BMJ Open. 2020 Oct 23;10(10):e037625. doi: 10.1136/bmjopen-2020-037625 (PMC7590366; doi:10.1136/bmjopen-2020-037625)
Supplement: Author's manuscript [file bmjopen-2020-037625.draft_revisions.pdf]

BMJ Open is committed to open peer review. As part of this commitment we make the peer review history of every article we publish publicly available.

When an article is published we post the peer reviewers' comments and the authors' responses online. We also post the versions of the paper that were used during peer review. These are the versions that the peer review comments apply to.

The versions of the paper that follow are the versions that were submitted during the peer review process. They are not the versions of record or the final published versions. They should not be cited or distributed as the published version of this manuscript.

BMJ Open is an open access journal and the full, final, typeset and author-corrected version of record of the manuscript is available on our site with no access controls, subscription charges or pay-per-view fees (http://bmjopen.bmj.com).

If you have any questions on BMJ Open's open peer review process please email info.bmjopen@bmj.com

BMJ Open

Essential evidence-based interventions during facility-based childbirth: clinical observations in northeast Nigeria

Journal:	*BMJ Open*
Manuscript ID	bmjopen-2020-037625
Article Type:	Original research
Date Submitted by the Author:	11-Feb-2020
Complete List of Authors:	Exley, Josephine; London School of Hygiene and Tropical Medicine Faculty of Infectious and Tropical Diseases, Department of Disease Control<br>Hanson, Claudia; Karolinska Institute, Public Health Sciences; London School of Hygiene and Tropical Medicine Faculty of Infectious and Tropical Diseases, Dept of Disease Control<br>Umar, Nasir; London School of Hygiene and Tropical Medicine Faculty of Infectious and Tropical Diseases, Disease Control<br>Willey, Barbara; London School of Hygiene and Tropical Medicine, Disease Control Department; London School of Hygiene and Tropical Medicine, Dept of Global Health and Development<br>Shuaibu, Abdulrahman; Gombe State Primary Health Care Development Agency<br>Marchant, Tanya; London Sch Hyg , Disease Control
Keywords:	Maternal medicine < OBSTETRICS, Quality in health care < HEALTH SERVICES ADMINISTRATION & MANAGEMENT, PRIMARY CARE

SCHOLARONE™
Manuscripts

I, the Submitting Author has the right to grant and does grant on behalf of all authors of the Work (as defined in the below author licence), an exclusive licence and/or a non-exclusive licence for contributions from authors who are: i) UK Crown employees; ii) where BMJ has agreed a CC-BY licence shall apply, and/or iii) in accordance with the terms applicable for US Federal Government officers or employees acting as part of their official duties; on a worldwide, perpetual, irrevocable, royalty-free basis to BMJ Publishing Group Ltd ("BMJ") its licensees and where the relevant Journal is co-owned by BMJ to the co-owners of the Journal, to publish the Work in this journal and any other BMJ products and to exploit all rights, as set out in our licence.

The Submitting Author accepts and understands that any supply made under these terms is made by BMJ to the Submitting Author unless you are acting as an employee on behalf of your employer or a postgraduate student of an affiliated institution which is paying any applicable article publishing charge ("APC") for Open Access articles. Where the Submitting Author wishes to make the Work available on an Open Access basis (and intends to pay the relevant APC), the terms of reuse of such Open Access shall be governed by a Creative Commons licence – details of these licences and which Creative Commons licence will apply to this Work are set out in our licence referred to above.

Other than as permitted in any relevant BMJ Author's Self Archiving Policies, I confirm this Work has not been accepted for publication elsewhere, is not being considered for publication elsewhere and does not duplicate material already published. I confirm all authors consent to publication of this Work and authorise the granting of this licence.

For peer review only

Essential evidence-based interventions during facility-based childbirth: clinical observations in northeast Nigeria

Josephine Exley[1], Claudia Hanson[1,2], Nasir Umar[1], Barbara Willey[3], Abdulrahman Shuaibu[4], Tanya Marchant[1*]

[1] Department of Disease Control, London School of Hygiene and Tropical Medicine, UK

[2] Department of Public Health Sciences–Global Health, Karolinska Institutet, Stockholm, Sweden

[3] Department of Infectious Disease Epidemiology, London School of Hygiene and Tropical Medicine, UK

[4] The Executive Secretary, Gombe State Primary Health Care Development Agency, Nigeria

Correspondence to:
Josephine Exley
LSHTM
Keppel Street
London
WC1E 7HT
United Kingdom
Josephine.Exley@lshtm.ac.uk

Word Count: 4,764

For peer review only

Abstract

Objectives:

To examine evidence-based preventive and promotive interventions provided to women, and subsequently their newborns, during childbirth in a high-mortality setting.

Design:

Cross-sectional observations of care provided to women, and their newborns during the intra- and immediate postpartum period using a standardised checklist capturing health worker behaviours regarding lifesaving and respectful care.

Setting:

Ten high-volume primary healthcare facilities in the high mortality setting of Gombe State, northeast Nigeria.

Main outcome measures:

Data on 49 measures of internationally recommended evidence-based interventions and good practice. We developed a composite score of 10 essential items.

Results:

1,875 women were admitted to a health facility during the observation period; of these, 1,804 gave birth in the facility and did not experience an adverse event or death. On average, women received half of measures in our composite score. Many clinical interventions around the time of birth were routinely implemented, including provision of uterotonic (90% [95%CI 93-98]), whereas risk-assessment measures, such as history-taking or checking vital signs were rarely completed: just 2% (95%CI 2-7) of women had temperature taken and 12% (95%CI 9-16) were asked about complications during pregnancy.

Conclusions:

The majority of women did not receive the recommended routine processes of childbirth care they and their newborns needed to benefit from their choice to deliver in a health facility. In particular, few benefited from even basic risk-assessments, leading to missed opportunities to identify risks. To continue with the recommendation of childbirth care in primary healthcare in high mortality settings like Gombe it is crucial that birth attendant capacity, capability and prioritisation processes are addressed.

For peer review only

Strengths and limitations of this study

- Clinical observations provide unique insight into the provision of evidence-based practices during childbirth and highlights clear areas for action in this setting, actions that are also likely to be relevant elsewhere.
- This study has a relatively large sample size. Over 1,850 women were observed across five rounds of data collection.
- The study protocol prioritised events closest to birth, as such 40% of women were not observed during the first stage of labour. Missing observations are not anticipated to impact findings as non-observation was likely to be random.
- The relative consistency overtime and low levels of implementation of many measures suggest that any impact of being observed was minimal.
- This study was completed in the 10 primary care facilities with the highest volume of births in Gombe State. It is anticipated that these facilities represent the 'best' care available at the primary level and that findings are likely to overestimate the provision of evidence-based care available to the wider population.

For peer review only

Introduction

Global efforts to reduce preventable maternal and newborn mortality have focused on skilled attendance at birth. This has resulted in marked increases in birth with a skilled birth attendant, mainly operationalised through childbirth in facilities. However, these increases have not been accompanied by the anticipated improvements in maternal and newborn outcomes in many low and middle income countries [1-4], prompting a closer examination of the quality of care provided [5-7]. The growing body of evidence from low income settings frequently highlights low provider skills and limited facility capability to provide good-quality care at birth [1 8-10]. A recent review found large declines in the proportion of individuals estimated to have skilled birth attendance when some measure of quality was taken into account [11].

Good quality of care includes the timely and appropriate use of evidence-based clinical and non-clinical interventions that are acceptable to women [12-14]. The World Health Organisation (WHO) has developed evidence-based guidelines around intrapartum care, which have the potential to support healthcare providers to identify gaps in the quality of care and improve the provision and experience of care [15]. A continuing challenge, however, is that detailed evidence on the extent to which the recommended interventions are practised during routine childbirth in health facilities in low-income countries is scarce [1 16]. Available studies have predominantly focused on readiness for quality such as availability of drugs, supplies and other inputs, while neglecting processes of care [16]. In part, this is because these data are made available through surveys similar to the Service Provision Assessments [17], or through routine health information systems - information sources that include very little process quality data. Household surveys, including the Demographic and Health Survey and the Multiple Indicator Cluster Survey, do not collect extensive data on the content of care during childbirth, in part because evidence suggests women's self-reports have low validity [18 19]. The lack of data on coverage of evidence-based care during childbirth, impedes decision making around possible solutions. Although not a large-scale measurement solution, observations provide useful insight in to the behaviours of health workers and could support strategies to improve care.

In this study of birth observations, we report the maternal and newborn outcomes experienced by all women admitted for childbirth and postpartum haemorrhage in a sample of primary healthcare facilities in Gombe State, Nigeria. For women who had an uncomplicated labour, we examined the provision of evidence-based care provided to women, and subsequently their newborns, from initial assessment up to one hour postpartum.

Methods

We conducted direct observations of childbirth care in 10 primary healthcare facilities, in Gombe State, Nigeria, approximately every six months over a two-year period between June 2016 and August 2018.

Study setting

Gombe State is one of six states in northeast Nigeria, it has an area of 20,265km^2 and an estimated population of 2,857,042 [20]. The northeast region of Nigeria has some of the highest maternal and newborn death rates globally, estimated at 1,549 per 100,000 live births in 2015 and 33 per 1,000 live births in 2017, respectively [21 22].

Access to maternal healthcare services is relatively low in Gombe State. In 2018, 46% of women in the State reported at least one antenatal care visit from a doctor, nurse, midwife or nurse/midwife and 28% delivered in a health facility [23]. Recent work in Gombe on the drivers

For peer review only - http://bmjopen.bmj.com/site/about/guidelines.xhtml

of attending a facility for childbirth found that health system conditions including availability of staff, drugs and supply, and a clean environment had the biggest influence on respondents' decision around where to give birth [24].

Healthcare is predominantly delivered via a network of rural primary healthcare clinics run by the Gombe State Primary Healthcare Development Agency (GSPHCDA). In 2017, 460 primary healthcare clinics and 26 referral facilities provided childbirth services [25]. In response to the shortage and uneven distribution of health workers, under its task-shifting and task-sharing policy for essential healthcare services, Nigeria classifies community health extension workers (CHEWs) as skilled birth attendants [26]. In this paper, we define skilled birth attendants as a doctor, nurse or midwife, but also present evidence for CHEWs separately from unskilled birth attendants.

Led by the Gombe State Primary Healthcare Development Agency, intense NGO activity has been ongoing in 57 primary care facilities across Gombe State, aimed at increasing the quality of care since 2016 [27 28]. Interventions include training of CHEWs in all aspects of skilled birth attendance and basic emergency obstetric care, and improving the supply of essential maternal and newborn health commodities [29]. These facilities provide basic emergency obstetric and newborn care. Emergency care and complicated cases from these health facilities are referred to referral facilities.

Data collection

Data collection methods have been described in detail elsewhere [19 30]. Briefly, in November 2015, 10 primary care facilities were selected from the 57 facilities for an in-depth assessment of quality of care. These were the 10 primary care facilities with the highest number of births in the preceding six months, as recorded in the maternity register. The mean number of births per month in the 10 primary care facilities was 15.7 (standard deviation [sd] 12.0), compared to 4.3 (sd 6.3) births per facility per month across Gombe State as a whole [19].

Five rounds of data collection took place over the two-year study period. Each round lasted three weeks, during which observers aimed to collect data from a total of around 350 women. Two trained female observers (local midwives, not employed by the facility) and one clinical supervisor were assigned to each facility. Observers worked in 8- or 12-hour shifts to provide near continual data collection during the period. Depending on the observation team's work schedule, the first point of contact for any observation may have been during initial assessment of a newly admitted pregnant woman or at a later stage of labour. Observers aimed to observe all women who were admitted but they prioritised observing women during the second and third stage of labour and immediately postpartum rather than observing women earlier in the process. Observers stayed continuously with women from the first point of contact until the first hour after birth.

A structured clinical observation checklist, administered on a Lenovo A3300 tablet using CSPro version 7.0 (United States Census Bureau and ICF Macro, Suitland, MD, USA), was used to record the processes of care and birth attendant-client interactions. The content of the checklist was developed from the USAID-funded Maternal and Child Health Integrated Program's tool for observing vaginal births and the following complications: postpartum haemorrhage, pre-eclampsia/eclampsia and newborn asphyxia [31]. The checklist was piloted and modified to the Gombe context.

For peer review only - http://bmjopen.bmj.com/site/about/guidelines.xhtml

Defining provision of evidence-based care

For this analysis, the content of the clinical observation checklist was mapped against current recommendations for high quality mother and newborn care [13 15 32-35], resulting in 50 measures of evidence-based interventions and good practice during childbirth being included in the analysis (Box 1). The measures are grouped into four organising categories based on the stage of childbirth: (1) initial assessment; (2) first stage of labour; (3) second and third stage of labour; (4) immediate newborn and postpartum care.

Box 1 Measures of evidence based childbirth care included in this analysis[1]

History taking and initial assessment

- **Checks client card or asks client her age, length of pregnancy, and parity**
- **Asks whether woman has experienced any complications during current pregnancy**
- If woman has had any previous pregnancies, asks about complications during previous pregnancies
- Checks client card or asks client her HIV status
- Washes his/her hands with soap and water or uses disinfectant before initial any examination
- **Takes temperature**
- Takes blood pressure
- **Checks foetal heart rate with fetoscope/doppler/ultrasound**
- Performs vaginal examination
- Encourages the women to have a support person present during labour and birth
- **Explains procedures to woman (support person) before proceeding**
- Asks women (and support person) if she has any questions

First stage of labour

- Partograph used to monitor labour
- Washes his/her hands with soap and water or uses antiseptic prior to any examination of woman
- Wears high-level disinfected or sterile surgical gloves
- A support person (or companion) for mother is present at some point during labour
- At least once, explains what will happen in labour to woman (support person)
- At least once, encourages woman to consume fluids/food during labour
- Drapes woman (one drape under buttocks, one over abdomen)
- At least once, encourages/assists woman to ambulate and assume different positions during labour
- Following items of equipment laid out in preparation for birth:
  - Newborn face mask size 0 or 1 and self-inflating ventilation bag (250 or 500 mL)
  - Suction bulb
  - Disposable cord ties or clamps
  - Sterile scissors or blade
  - At least two cloths/blankets (one to dry; one to cover)

Second & third stage of labour

- Assisted by more than one health worker at one point during labour
- Mother gave birth in lithotomy position (on back)
- **As baby's head is delivered, supports perineum**
- **Administers uterotonic**
- Timing of uterotonic
- If care provided by a skilled birth attendant, applies traction to the cord while applying suprapubic counter traction

For peer review only

For peer review only - http://bmjopen.bmj.com/site/about/guidelines.xhtml

• **Assesses completeness of the placenta and membranes**
• **Assesses for perineal and vaginal lacerations**
• **A support person (companion) for mother is present at birth**
**Immediate newborn and postpartum care**
• Drying baby immediately after birth with towel
• Baby placed skin-to-skin with mother
• Bathing delayed for at least one hour
• Ties or clamps cord when pulsations stop, or by 2-3 minutes after birth (not immediately after birth)
• Cuts cord with clean blade or clean scissors
• Breastfeeding initiated within first hour
• Takes mother's vital signs within the first 15 minutes after birth
• Administers antibiotics to mother postpartum
• Checks baby's temperature within the first 15 minutes after birth
• Baby weighed
• Mother and newborn kept in same room after birth (rooming-in)
• Baby kept skin-to-skin for first hour
• Administers Vitamin K to newborn
• Provides tetracycline eye ointment prophylaxis
• Administers chlorhexidine to the newborn cord
• If the mother is HIV positive, administers ARVs to newborn

[1]Measures highlighted in bold are also included in a composite score

Study population

Data from the five data collection periods were combined into a single dataset. Observations were excluded from the dataset if the woman's outcome was not recorded. For all women observed, we mapped the different pathways from the reason women were admitted to the facility (childbirth or postpartum haemorrhage event) to their outcome. For women who experienced an uncomplicated labour the outcome of their baby was also mapped. An uncomplicated labour was defined as a woman who was sent to the ward for recuperation or discharged home after birth and who did not experience an adverse event (referral, postpartum haemorrhage or pre-eclampsia/eclampsia) or death.

For the analysis of the provision of essential evidence based care, our population of interest was women with an uncomplicated labour and detailed information on their care and that of their newborn are included here. Women who were admitted but experienced an adverse event or death were excluded from the analysis because of their individual medical needs. For measures related to newborn care the analysis was further restricted to newborns recorded as being alive and who did not require resuscitation care or were not referred to another facility.

Analysis

For each measure, percent frequencies and 95% confidence intervals were calculated, adjusted for clustering by primary healthcare facility and stratified by time point using the svyset and svy commands in STATA version 15.1. Results are presented graphically by time point to highlight any variability. A composite score of evidence-based care was generated as the sum of 10 measures across 'initial assessment' and 'second and third stage of labour' (highlighted in Box 1). The mean number of measures undertaken from this composite score was compared between newborns that did or did not experience an adverse event.

For peer review only - http://bmjopen.bmj.com/site/about/guidelines.xhtml

Consent to participate and ethical approval

All women attending the facility in active labour or experiencing postpartum haemorrhage were invited to participate at the time of admission, prior to receiving any care. All potential participants were provided with a study information sheet and a consent form in Hausa. The observer read the information sheet, explained the purpose of the study, the risks and benefits of participating and answered questions before seeking written consent from the woman and verbal consent from the birth attendant. Women who were not able to write their name were asked to provide a thumb print on the consent form. Participation was voluntary and participants were free to withdraw at any time.

Observers were required to prioritise the safety of the mother and newborn; protocols were established on the actions to take during any life-threatening events. Before each round of data collection, observers underwent four days of training on how to conduct unobtrusive observations, the safety and confidentiality protocols and how to ensure consistency of rating between observers.

Ethical approval for this study were obtained from the London School of Hygiene & Tropical Medicine (reference 14091) and the Health Research Ethics Committees for Nigeria (reference NHREC/01/01/2007) and Gombe State (reference ADM/S/658/Vol. II/66).

Patient and Public Involvement

Patients and the public were not involved in the design, conduct, reporting or dissemination plans of our research. Observations were recorded in English and pre-testing completed in health facilities by staff.

Results

In total 1,875 women were admitted to a facility during the five observations periods. The median age of the women was 24 years (interquartile range [IQR] 20–29) and for 19% it was their first birth: median parity 2 (IQR 1–5). The median gestational age was 39 weeks (IQR 38–39); 6% of women had a gestational age of less than 37 weeks and for 21% of women gestational age was not recorded on their client card and/or they did not know. Eleven percent of women were seen by a skilled birth attendant (doctor, midwife, nurse), 39% by a community health extension worker (CHEW) or junior CHEW and 50% by an 'other' birth attendant. 'Other' included traditional birth attendants, environmental health officers/technicians/assistants and health attendant/assistant.

We first present the outcomes for all 1,875 women who were admitted to the facility, followed by the coverage of the provision of evidence based care measures, outlined in Box 1, for the 1,804 women who had an uncomplicated labour and the 1,635 babies born to these women who did not experience an adverse event or death. The full table of results disaggregated by time-point is presented in supplementary material.

Outcomes

Figure 1 presents the outcomes for the 1,875 women admitted to a facility during the five observation periods. Nine women were admitted with postpartum haemorrhage and 1,866 were admitted for childbirth. Fourteen women admitted for childbirth were referred to another facility during labour. The main reason for referral was prolonged labour (Panel 1). Of the 1,852 women who gave birth in the facility, all delivered vaginally with one woman recorded as requiring an assisted delivery; caesarean sections are not available in primary healthcare

For peer review only - http://bmjopen.bmj.com/site/about/guidelines.xhtml

facilities in this setting. Over half of births occurred on a weekday (56% [1,031/1,852]) between 7am and 7 pm (58% [1,067/1,852]). Two per cent (34/1,852) of deliveries were multiple births.

Postpartum, 45 women experienced an adverse event and three women died: the mortality rate was 1.6 per 1,000 women. Four women were referred intrapartum due to complications associated with multiple births and seven women postpartum (Panel 1).

For the babies born to women who had an uncomplicated labour, 96 received resuscitation care and 79 died, 23% (18/79) of whom died after an attempted resuscitation event: the perinatal mortality rate was 44.1 per 1,000 newborns (Figure 1). For babies born to women who experienced an adverse an event or died, 19% (9/48) were referred and four died: the perinatal morality rate among this group was 83.3 per 1,000 newborns.

Panel 1 Observers free text comments on reason for referral

In total 25 women were referred to another facility. Fourteen during labour - of whom one had eclampsia and three had severe pre-eclampsia; four women with multiple births were referred intrapartum; and seven were referred postpartum - of whom two had eclampsia and three experienced a postpartum haemorrhage event.

The most common reason for referral during labour was prolonged labour. One observer noted "*the mother was referred to a specialist hospital, because no progress of labour, [...] [the mother was] 4cm [dilated] after about 18 hours*" and another commented *"she was referred due to non-progress of labour despite augmentation with oxytocin*". One woman was referred because her baby was in a breech position.

For the four women diagnosed with severe pre-eclampsia/eclampsia during labour, insufficient assessment and monitoring of the woman during labour was noted: "*there were delays in needed treatment. No observations undertaken during the first hour of admission to detect disease condition and take appropriate action",* whilst another observed *"woman was admitted for normal labour but only vaginal examination was observed without physical nor vital signs or urine test".*

Five women were referred due to complications with the birth of the second twin: "*mother delivered first twin alive, mother referred […] as second twin was in transverse lie with hand prolapse"* and *"mother came with a history of twins. Delivered one at 9:48pm but the other baby refused to come out and the mother was referred in the morning around 8:39am*".

Two women with eclampsia were referred postpartum because their condition could not be stabilised. Both women experienced convulsions and lost consciousness. They both received magnesium sulphate before referral: one woman's baby was born alive and referred with the mother; the other was a fresh stillbirth.

For the three women who experienced a postpartum haemorrhage event none had severe postpartum haemorrhage (abnormal bleeding of more than 1000 mL). All received oxytocin in the facility before referral: one woman's condition was reported not to be stable and the baby required resuscitation before referral. For the other two women, who were reported to be stable, their baby was referred with them.

The remaining woman referred postpartum was referred because "*mother had a retain placenta, mentor and birth attendant tried everything but it failed*". The newborn was referred with their mother.

For peer review only - http://bmjopen.bmj.com/site/about/guidelines.xhtml

Provision of evidence-based care

All 1,804 women who had an uncomplicated labour were observed during the second and third stage of labour. Observers were required to prioritise the second and third stage of labour so the number of women and newborns observed during other stages of childbirth varied (Table 1).

Table 1 Proportion of all women admitted for childbirth who were observed, disaggregated by stage of care

Stage of care	Percentage observed	
	Women	Newborns
Initial assessment and history taking	99.8% (1,801/1,804)	n/a
First stage of labour	59.3% (1,069/1,804)	
Second & third stage of labour	100% (1,804/1,804)	
Immediate newborn & postpartum care	94.1% (1,697/1,804)	98.9% (1,617/1,635)

History taking and Initial assessment

In total, 1,801 women were observed during the initial assessment and history taking. Birth attendants were routinely observed to check the women's record for or, if not available, asked women's age, length of pregnancy and parity; 80% (95%CI 73-86), see

Figure 2. However, other aspects of history taking including asking about complications during both current and, if relevant, previous pregnancies were very low: 12% (95%CI 9-16) and 18% (95%CI 15-23), respectively. The most common complications that birth attendants asked about for the current pregnancy were fever (37% [81/218]), vaginal bleeding (32% [70/218]) and severe abdominal pain (25% [54/218]).

The majority of women were encouraged to have a support person present during labour and birth (63% [95%CI 53–72]). While around two thirds of birth attendants explained procedures to woman (and their support person) before proceeding (66% [95%CI 57–74]), less than a third of women (and their support person) were asked if they had any questions (32% [95%CI 25–40]).

Vaginal examination was almost universal (98% [95%CI 97-99]) but the proportion of women who had their temperature and blood pressure measured, and the foetal heart rate checked was low: 4% (95%CI 2-7), 30% (95%CI 23-39) and 22% (95%CI 16–29), respectively (

Figure 2). Few birth attendants washed their hands with soap and water or antiseptic before examining women (28% [95%CI 21-37]).

.

For peer review only - http://bmjopen.bmj.com/site/about/guidelines.xhtml

First stage of labour

One thousand and sixty-nine women were observed during first stage of labour. Partograph was used to monitor labour in just 24% (95%CI 18-32) of cases (

Figure 3). Data were not collected on whether there was a delay in the progress of labour but 17% (95%CI 12-24) of women had their labour augmented with oxytocin and 6% (95%CI 4-9) of women's membranes were artificially ruptured.

The median number of vaginal examinations undertaken during the first stage of labour was one (IQR 1-2). Birth attendants were observed to wash their hands with soap & water or use antiseptic 39% (95%CI 30-49) of the time prior to any examination of woman, although a greater proportion were observed to wear high-level disinfectant or surgical gloves when performing the vaginal exam 76% (95%CI 64-85).

Seventy two per cent (95%CI 63–79) of women had a support person present at some point during labour (

Figure 3). Just under half of birth attendants were observed to explain to the woman (and their support person) what will happen during labour (49% [95%CI 42–56]). The majority of birth attendants were observed to encourage the woman to consume fluids/food at least once during labour (89% [95%CI 85-92]) and to encourage or help woman to ambulate and assume different positions (73% [95%CI 66–80]). During more than half of births observed, birth attendants were observed to drape women with a cloth, one under the buttocks and one over the abdomen (59% [95%CI 48–69]).

Data were collected on whether clean cloths/blankets, tie or cord clamp, sterile blade to cut cord, suction device and bag and mask (either size 0 or 1) were laid out in preparation for birth. Cord clamps, sterile blade and at least two cloths/blankets were available for over 90% of women (Figure 3). However, preparation of a bag and mask was substantially lower at 40% (95%CI 31-50).

Second and third stage of labour

All 1,804 women were observed during the second and third stage of labour. Just over half of women observed were assisted at some point during the second and third stage of labour by more than one healthcare worker (57% ([95%CI 50-63]) and 36% (95%CI 27-45) had a support person present (Figure 4). Women universally gave birth in the lithotomy position (97% [95% CI 93-98]). The use of episiotomy was very low (1% [95%CI 1-2]). Other inappropriate practices were also low: 3% (95%CI 1-7) fundal pressure, 1% (95%CI 0-2) excessive stretching of perineum and less than 1% had lavage of uterus.

The use of prophylactic uterotonic drugs immediately after birth was universal (96% [95%CI 93-98]), and 65% (95%CI 56-73) of birth attendants checked for the presence of a second baby before administering a uterotonic. Of women that received a uterotonic, the majority received either oxytocin (58% [999/1,735]) or misoprostol (42% [733/1,735]). Fourteen per cent (95%CI 9-20) of deliveries where uterotonics were administered were given within 1 minute of birth and for 52% (95%CI 44-57) of women a uterotonic was administered more than 3 minutes after birth. Despite the recent recommendation that uterine massage should not be performed if women have received a uterotonic, 75% (95%CI 69-80) of birth attendants were observed to perform uterine massage immediately following the birth of the placenta.

For peer review only - http://bmjopen.bmj.com/site/about/guidelines.xhtml

The use of controlled cord traction was high and despite recommendations that only a skilled birth attendant should undertake controlled cord traction, it was consistently undertaken by all cadres: skilled 91% (95%CI 83-96), CHEW 85% (95%CI 76-92) and 'other' birth attendant 84% (95%CI 77-89).

Following the birth of the placenta, 49% (95%CI 39-58) of birth attendants were observed to assess the completeness of placenta and membranes, a considerably higher proportion (87% [95%CI 79-92]) assessed for perineal and vaginal lacerations.

Immediate newborn and postpartum care

During the immediate postpartum period, 1,617 newborns and 1,697 women were observed. The majority of newborns received all three elements of thermal care (immediate drying, skin-to-skin and not bathing in the first hour) and clean cord care (ties or clamps cord when pulsation stop or within 2-3 minutes after birth and cuts cord with clean scissors or blade): 71% (95%CI 60-80) and 92% (95%CI 89-95), respectively (

Figure 5). Eighty six per cent (95%CI 75-93) of newborns had chlorohexidine applied to their cord within the first hour of birth. However, breastfeeding was initiated within the first hour after birth in just under half of newborns: 49% (95%CI 39-59).

Eighty-nine per cent (95%CI 79-95) of newborns were weighed. Of these infants, 95% (1,122/1,182) weighed more than 2.5kg (Figure 5). Mothers and newborns were universally kept in the same room after birth (rooming in), but only 61% (95%CI 51-71) were kept skin-to-skin during the first hour.

For the fifteen babies born to mothers' known to have HIV, 33% (95%CI 14-60) were observed to receive antiretroviral therapy. The proportion of newborns on postnatal care wards who received a vitamin K injection, tetracycline eye ointment and had their temperature checked within 15 minutes after birth was close to zero throughout the study period (Figure 5): 0.1% (95%CI 0-0.4), 4% (95%CI 2-7) and 2% (95%CI 1-5), respectively.

Just 3% (95%CI 2-6) of women had their vital signs checked 15 minutes after birth and 2% (95%CI 1-5) of women received antibiotics.

Composite score of evidence-based care

A composite score of evidence-based care was generated as the sum of 10 measures: checks client card or asks client her age, length of pregnancy, and parity; asks whether woman has experienced any complications during current pregnancy; takes temperature; checks foetal heart rate with fetoscope/doppler/ultrasound; as baby's head is delivered, supports perineum; administers uterotonic; assesses completeness of the placenta and membranes; assesses for perineal and vaginal lacerations; and a support person (companion) for mother is present at birth.

Overall, less than one per cent of women received all ten components of care (7/1,793). On average women received 5.4 (sd 1.6) out of the 10 components. The mean number of measures received was similar for mothers' whose newborns did or did not experience an adverse event: 5.5 (sd 1.5) vs. 5.4 (sd 1.6), respectively.

Discussion

Our observations in ten high volume primary healthcare facilities in northeast Nigeria indicated that, while some essential processes of childbirth care were performed for almost all women

For peer review only - http://bmjopen.bmj.com/site/about/guidelines.xhtml

and newborns, the proportion of women who consistently received evidence-based care during uncomplicated labour was low. Three percent of women (48/1,852) who gave birth at the facility experienced major risks and complications while at the facility, including six severe pre-eclampsia/eclampsia and six twin deliveries. Three women died. There were large numbers of perinatal deaths and newborn referrals among this group of women. Further, among women who we defined as having an uncomplicated labour 79 perinatal deaths were recorded, of which almost three-quarters were intrapartum stillbirths.

On average, women received only half of the measures in our summary score that captured elements of care from initial assessment through to the immediate postpartum period. Our study highlights substantial variation in the implementation of recommended evidence-based interventions both within and across the different stages of childbirth: during all four stages, fewer than half of measures reached more than 80% of women. Implementation was highest for clinical interventions at the time of birth that have received international attention, for example, the provision of uterotonic, thermal and clean cord care. Implementation was lowest for measures designed as risk assessments, for example, history-taking or checking vital signs. All measures related to risk assessment during the initial assessment (asks about complications in current and previous pregnancies, takes temperature and blood pressure, checks foetal heart rate) were completed for fewer than 30% of women observed.

The evidence-based care measures included in this study were based on WHO guidelines for essential intrapartum care [15]: their implementation should be covered in basic pre-service training for midwives, nurses and Community Health Extension Workers in this setting; and they require only basic supplies of equipment. However, half of all observed births were attended by 'other' staff who would be defined as unskilled birth attendants both globally and in the Nigerian context: not doctors, nurses or midwives, nor Community Health Extension Workers who have received additional training under Nigeria's task-shifting policy. Even so, far fewer than half of the women received many measures, raising questions around the behaviours of birth attendants who have received training [36]. Further, although some variation was observed over time, in general, coverage of individual measures remained relatively constant throughout the observation period, including for a number of practices that are no longer recommended by WHO, for example, uterine massage [37]. Taken together, this study suggests that there may be limited opportunities for birth attendants to keep their knowledge up-to-date and enhance their skills once in post. Implementation research is needed in this setting to identify mechanisms to improve implementation, such as the use of checklists and in-service supervision and coaching, which has shown potential in other settings to improve uptake of and adherence to essential birth practices [32 38].

A potential alternative reason for the variation in care across the continuum is that some birth attendants were working alone for extended periods. In these circumstances, birth attendants may prioritise interventions during the third stage of labour and immediate newborn care. Higher workload, as a result of staff shortages, has been found to limit health workers time for history-taking, thorough assessment of women, and ability to provide timely care [39]. Further, when working alone, birth attendants struggle to implement multiple recommended interventions simultaneously, for example, administering uterotonic and providing essential newborn care. While administering uterotonic was universal, less than half of women received a uterotonic within three minutes of birth. The importance of the exact timing of the application of uterotonic is not well-established, and as such, it is not clear whether this should be prioritised over immediate newborn care interventions [40 41] and clearer guidelines may be

For peer review only - http://bmjopen.bmj.com/site/about/guidelines.xhtml

needed if it is important to prioritise certain components of care that cannot be done simultaneously by a single health worker [42].

Poor assessment of women and newborns before and after birth has previously been documented in India and other African settings [43-45]. Observations of WHO-recommended practices for screening of pre-eclampsia/eclampsia in six sub-Saharan African countries, similarly found that a low proportion of women admitted to labour and delivery services were asked about danger signs, but substantially more women (77%) had their blood pressure checked on admission [44]. Low implementation of evidence-based care measures during the initial assessment and postpartum period indicate potential missed opportunities to identify and manage complications, as evident in the referral cases (Panel 1). Referrals post- and intrapartum included women with high-risk pregnancies such as severe pre-eclampsia/eclampsia, breech position and twins who should have been referred to a higher level of care [46]. Given, in this setting, only 37% of women attended at least four antenatal care visits and 10% of women and 7% of newborns had a postnatal check within 2-days of birth in 2015, proper risk assessment in the facility is vital [47].

Poor quality of care has been acknowledged as a critical roadblock in Nigeria's attempt to reach universal health coverage. The Federal government has committed to strengthening Nigeria's health system, particularly primary care, and specifically to accelerate the reduction of maternal and neonatal mortality by expanding access to, and quality of, maternal and child health services [48]. The implementation of the national strategy is supported by Nigeria's participation in the Network for Improving Quality of Care for Maternal, Newborn and Child Health (Quality of Care Network) [49]. The processes of care prioritised in these strategies focus on clinical interventions at the time of birth, including use of uterotonic drugs, skin-to-skin and chlorhexidine for umbilical cord care – interventions that were found to be routinely implemented in this low resource primary care setting. This study's findings suggest if these policies are to have an impact they need to extend their focus to also include basic risk-assessment. Further, while they aim to be support health workers at the health facilities through quality improvement cycles, clinical mentoring and peer-to-peer learning they may also need to consider the quality of pre-service training.

Strengths and Limitations

This study provides unique insight into the provision of evidence-based practices during childbirth and highlights clear areas for action in this setting, actions that are also likely to be relevant elsewhere. A particular strength was the relatively large sample size from multiple time points although, due to the study protocol to prioritise events closest to birth, over 40% of women were not observed during the first stage of labour. The missing observations are not anticipated to impact on findings as non-observation was likely to be random and women who were not observed during the first stage of labour are not likely to systematically differ from those who were observed. Being observed may have impacted on birth attendants' behaviour, however, the relative consistency overtime and the low levels of implementation of many measures suggest that any effect of being observed was minimal.

This study was completed in the 10 primary care facilities with the highest volume of births in Gombe State and is not therefore representative of all primary care facilities. It is anticipated that these facilities represent the 'best' care available at the primary level and therefore findings are likely to overestimate the provision of evidence-based care available to the wider population. These findings support the growing body of evidence that giving birth in a primary

For peer review only - http://bmjopen.bmj.com/site/about/guidelines.xhtml

health facility might not be sufficient to ensure the effective care of women and newborns [1 4 50], and raise questions about the safety and quality of rural primary care facilities that have low-volume of deliveries [16 51].

Conclusions

The recommendation for women to deliver in health facilities is designed to improve birth outcomes. This study of clinical observations of labour and the immediate postpartum period in primary facilities in Gombe State has revealed that, while some processes of clinical care were well adhered to, most women do not receive the complete repertoire of childbirth care that they and their newborns needed to benefit from their choice to deliver in a health facility. In particular, few women or newborns benefited from even basic risk assessments, leading to missed opportunities to identify risks and consequently late referrals and deaths. To continue with the recommendation of childbirth care in primary healthcare in high mortality settings like Gombe it is crucial that birth attendant capacity, capability and prioritisation processes are purposively addressed.

For peer review only

Supporting information

Acknowledgements
The authors would like to thank Data Research and Mapping Consult for coordinating the data collection, the Gombe State Primary Health Care Development Agency, Gombe State Ministry of Health and our partners Society for Family Health and Pact Nigeria for their support in carrying out this study. We would like to thank all the participants who contributed to our study.

Ethical approval and consent to participate
This research was conducted with approval from the Federal Ministry of Health Abuja, Nigeria, the State Ministry of Health Gombe State, Nigeria and the London School of Hygiene & Tropical Medicine (reference 12181).

Funding
This work is part of the IDEAS (Informed Decisions for Actions to improve maternal and newborn health) project. IDEAS is funded through a grant from the Bill & Melinda Gates Foundation to the London School of Hygiene & Tropical Medicine. Gates Global Health Grant Number: OPP1149259. The funder had no role in study design, data collection and analysis, decision to publish or preparation of the manuscript.

Competing interests
We have read the BMJ policy and the authors declare that they have no competing interests.

Authors' contributions
JE conducted the analysis and drafted the manuscript. CH supported the refinement of our definition of evidence-based care and reviewed the manuscript for intellectual content. NU, BW and AS all critically reviewed the manuscript for intellectual content. TM conceived the study and drafted the manuscript. All authors have read and approved the final version of the manuscript.

Data sharing
The data are available from the principal investigator of the IDEAS project and co-author for this manuscript, Dr Tanya Marchant ORCID id 0000-0002-4228-4334. Reuse permitted on request.

Patient consent and confidentiality
Participation in the study was voluntary and participants were free to withdraw from the study at any time without giving a reason. Researchers explained the purpose of the study and answered any questions participants had. The free and informed consent of all women and birth attendants observed was obtained before participating.

Patient and public involvement
Patients or the public were not involved in the design, conduct, reporting or dissemination plans of our research. Observations were recorded in English and pre-testing completed in health facilities by staff.

For peer review only

For peer review only - http://bmjopen.bmj.com/site/about/guidelines.xhtml

References

1. Campbell OM, Calvert C, Testa A, et al. The scale, scope, coverage, and capability of childbirth care. *Lancet (London, England)* 2016;388(10056):2193-208. doi: 10.1016/s0140-6736(16)31528-8 [published Online First: 2016/09/20]
2. Gabrysch S, Nesbitt RC, Schoeps A, et al. Does facility birth reduce maternal and perinatal mortality in Brong Ahafo, Ghana? A secondary analysis using data on 119 244 pregnancies from two cluster-randomised controlled trials. *The Lancet Global health* 2019;7(8):e1074-e87. doi: 10.1016/s2214-109x(19)30165-2 [published Online First: 2019/07/16]
3. Randive B, Diwan V, De Costa A. India's Conditional Cash Transfer Programme (the JSY) to Promote Institutional Birth: Is There an Association between Institutional Birth Proportion and Maternal Mortality? *PloS one* 2013;8(6):e67452. doi: 10.1371/journal.pone.0067452 [published Online First: 2013/07/05]
4. Scott S, Ronsmans C. The relationship between birth with a health professional and maternal mortality in observational studies: a review of the literature. *Tropical medicine & international health : TM & IH* 2009;14(12):1523-33. doi: 10.1111/j.1365-3156.2009.02402.x [published Online First: 2009/10/02]
5. Kruk ME, Gage AD, Joseph NT, et al. Mortality due to low-quality health systems in the universal health coverage era: a systematic analysis of amenable deaths in 137 countries. *Lancet (London, England)* 2018;392(10160):2203-12. doi: 10.1016/s0140-6736(18)31668-4 [published Online First: 2018/09/10]
6. Kruk ME, Gage AD, Arsenault C, et al. High-quality health systems in the Sustainable Development Goals era: time for a revolution. *The Lancet Global Health* 2018;6(11):e1196-e252. doi: 10.1016/S2214-109X(18)30386-3
7. World Health Organization. Strategies towards ending preventable maternal mortality (EPMM). Geneva: World Health Organization 2015:44 p. Executive summary published as WHO/RHR/15.03.
8. Merali HS, Lipsitz S, Hevelone N, et al. Audit-identified avoidable factors in maternal and perinatal deaths in low resource settings: a systematic review. *BMC pregnancy and childbirth* 2014;14:280. doi: 10.1186/1471-2393-14-280 [published Online First: 2014/08/19]
9. Hobbs AJ, Moller AB, Kachikis A, et al. Scoping review to identify and map the health personnel considered skilled birth attendants in low-and-middle income countries from 2000-2015. *PloS one* 2019;14(2):e0211576. doi: 10.1371/journal.pone.0211576 [published Online First: 2019/02/02]
10. Radovich E, Benova L, Penn-Kekana L, et al. 'Who assisted with the delivery of (NAME)?' Issues in estimating skilled birth attendant coverage through population-based surveys and implications for improving global tracking. *BMJ global health* 2019;4(2):e001367. doi: 10.1136/bmjgh-2018-001367 [published Online First: 2019/05/30]
11. Amouzou A, Leslie HH, Ram M, et al. Advances in the measurement of coverage for RMNCH and nutrition: from contact to effective coverage. *BMJ global health* 2019;4(Suppl 4):e001297. doi: 10.1136/bmjgh-2018-001297 [published Online First: 2019/07/13]
12. Tuncalp, Were WM, MacLennan C, et al. Quality of care for pregnant women and newborns-the WHO vision. *BJOG : an international journal of obstetrics and gynaecology* 2015;122(8):1045-9. doi: 10.1111/1471-0528.13451 [published Online First: 2015/05/02]
13. Miller S, Abalos E, Chamillard M, et al. Beyond too little, too late and too much, too soon: a pathway towards evidence-based, respectful maternity care worldwide. *Lancet (London, England)* 2016;388(10056):2176-92. doi: 10.1016/s0140-6736(16)31472-6 [published Online First: 2016/09/20]
14. Oladapo O, Tunçalp Ö, Bonet M, et al. WHO model of intrapartum care for a positive childbirth experience: transforming care of women and babies for improved health and

For peer review only - http://bmjopen.bmj.com/site/about/guidelines.xhtml

wellbeing. *BJOG: An International Journal of Obstetrics & Gynaecology* 2018;125(8):918-22. doi: 10.1111/1471-0528.15237
15. World Health Organization. WHO recommendations: intrapartum care for a positive childbirth experience. Geneva: World Health Organization, 2018.
16. Kruk ME, Leslie HH, Verguet S, et al. Quality of basic maternal care functions in health facilities of five African countries: an analysis of national health system surveys. *The Lancet Global health* 2016;4(11):e845-e55. doi: 10.1016/s2214-109x(16)30180-2 [published Online First: 2016/10/22]
17. The DHS Program. Service Provision Assessment (SPA) Overview.online: https://dhsprogram.com/What-We-Do/Survey-Types/SPA.cfm [accessed on 16 January 2020]
18. Moxon SG, Ruysen H, Kerber KJ, et al. Count every newborn; a measurement improvement roadmap for coverage data. *BMC pregnancy and childbirth* 2015;15 Suppl 2:S8. doi: 10.1186/1471-2393-15-s2-s8 [published Online First: 2015/09/24]
19. Bhattacharya AA, Allen E, Umar N, et al. Monitoring childbirth care in primary health facilities: a validity study in Gombe State, northeastern Nigeria. *Journal of global health* 2019;9(2):020411. doi: 10.7189/jogh.09.020411 [published Online First: 2019/07/31]
20. Gombe State Government. History of Gombe State, Nigeria. 2018;online: http://gombestate.gov.ng/history-2/ [accessed on 9 September 2019]
21. Izugbara CO, Wekesah FM, Adedini SA. Maternal Health in Nigeria. A Situation Update. Nairobi, Kenya: African Population and Health Research Centre, 2016.
22. National Bureau of Statistics and United Nations Children's Fund. Multiple Indicator Cluster Survey 2016–17, Survey Findings Report. Abuja: Nigeria National Bureau of Statistics and United Nations Children's Fund, 2017.
23. National Population Commission and ICF. Nigeria Demographic and Health Survey 2018. Key Indicators Report. Abuja, Nigeria, and Rockville, Maryland, USA, 2019.
24. Umar N, Quaife M, Exley J, et al. Toward improving respectful maternity care: a discrete choice experiment with rural women in northeast Nigeria. forthcoming
25. Bhattacharya AA, Umar N, Audu A, et al. Quality of routine facility data for monitoring priority maternal and newborn indicators in DHIS2: A case study from Gombe State, Nigeria. *PloS one* 2019;14(1):e0211265. doi: 10.1371/journal.pone.0211265 [published Online First: 2019/01/27]
26. Federal Ministry of Health. Task-shifting and task-sharing policy for essential health care services in Nigeria. Abuja, Nigeria, 2014.
27. IDEAS team. Informed Decisions for Actions in Maternal and Newborn Health 2010-17 Report: What works, why and how in maternal and newborn health. online: https://ideaslshtmacuk/wp-content/uploads/2017/09/LSHTM_IDEAS_PhaseIReport_Web-reducedpdf [accessed on 9 August 2019]: IDEAS, London School of Hygiene & Tropical Medicine, 2017.
28. Makowiecka K, Marchant T, Betemariam W, et al. Characterising innovations in maternal and newborn health based on a common theory of change: lessons from developing and applying a characterisation framework in Nigeria, Ethiopia and India. *BMJ global health* 2019;4(4):e001405. doi: 10.1136/bmjgh-2019-001405 [published Online First: 2019/08/14]
29. Makowiecka K. The Pathway to Improved Maternal and Newborn Health Outcomes. Use of data for maternal and newborn health in Gombe State, Nigeria. online: https://ideaslshtmacuk/wp-content/uploads/2017/08/Makowiecka_IDEAS_Pathway_Improved_Maternal_Newborn_Health_Gombe_Nigeria_2016pdf [accessed on 9 September 2019]: IDEAS, London School of Hygiene & Tropical Medicine, 2016.
30. Marchant T. Change in maternal and newborn health care. Interactions between families and frontline workers - their frequency, quality and equity - and coverage of interventions of mothers and newborns. Report from Gombe State, Nigeria, 2012-2015. online: https://ideaslshtmacuk/wp-content/uploads/2017/08/Marchant_IDEAS_Gombe_State_Follow-

For peer review only - http://bmjopen.bmj.com/site/about/guidelines.xhtml

upSurvey_2016pdf [accessed on 9 September 2019]: IDEAS, London School of Hygiene & Tropical Medicine, 2016.

31. USAID. Maternal and Newborn Quality of Care Surveys. *Maternal and Child Health Integrated Program* 2013;online: https://www.mchip.net/qocsurveys/ [accessed on 10 September 2019]
32. Marx Delaney M, Maji P, Kalita T, et al. Improving Adherence to Essential Birth Practices Using the WHO Safe Childbirth Checklist With Peer Coaching: Experience From 60 Public Health Facilities in Uttar Pradesh, India. *Global Health: Science and Practice* 2017;5(2):217-31. doi: 10.9745/GHSP-D-16-00410
33. World Health Organization. Standards for improving quality of maternal and newborn care in health facilities. Geneva: World Health Organization, 2016.
34. World Health Organization. Consultation on improving measurement of the quality of maternal, newborn and child care in health facilities. Geneva: World Health Organization, 2013.
35. World Health Organization, United Nations Population Fund, UNICEF. Pregnancy, childbirth, postpartum and newborn care. A guide for essential practice (3rd edition). Geneva: World Health Organization, 2015.
36. Harvey SA, Blandon YC, McCaw-Binns A, et al. Are skilled birth attendants really skilled? A measurement method, some disturbing results and a potential way forward. *Bulletin of the World Health Organization* 2007;85(10):783-90. doi: 10.2471/blt.06.038455 [published Online First: 2007/11/27]
37. World Health Organization. WHO recommendations for the prevention and treatment of postpartum haemorrhage. Geneva: World Health Organization, 2012.
38. Semrau KEA, Hirschhorn LR, Marx Delaney M, et al. Outcomes of a Coaching-Based WHO Safe Childbirth Checklist Program in India. *The New England journal of medicine* 2017;377(24):2313-24. doi: 10.1056/NEJMoa1701075 [published Online First: 2017/12/14]
39. Munabi-Babigumira S, Glenton C, Lewin S, et al. Factors that influence the provision of intrapartum and postnatal care by skilled birth attendants in low- and middle-income countries: a qualitative evidence synthesis. *Cochrane Database of Systematic Reviews* 2017(11) doi: 10.1002/14651858.CD011558.pub2
40. Vogel JP, Williams M, Gallos I, et al. WHO recommendations on uterotonics for postpartum haemorrhage prevention: what works, and which one? *BMJ global health* 2019;4(2):e001466. doi: 10.1136/bmjgh-2019-001466
41. Bartlett L, Cantor D, Lynam P, et al. Facility-based active management of the third stage of labour: assessment of quality in six countries in sub-Saharan Africa. *Bulletin of the World Health Organization* 2015;93(11):759-67. doi: 10.2471/blt.14.142604 [published Online First: 2015/11/10]
42. Moller AB, Newby H, Hanson C, et al. Measures matter: A scoping review of maternal and newborn indicators. *PloS one* 2018;13(10):e0204763. doi: 10.1371/journal.pone.0204763 [published Online First: 2018/10/10]
43. de Graft-Johnson J, Vesel L, Rosen HE, et al. Cross-sectional observational assessment of quality of newborn care immediately after birth in health facilities across six sub-Saharan African countries. *BMJ open* 2017;7(3):e014680. doi: 10.1136/bmjopen-2016-014680 [published Online First: 2017/03/30]
44. Rawlins B, Plotkin M, Rakotovao JP, et al. Screening and management of pre-eclampsia and eclampsia in antenatal and labor and delivery services: findings from cross-sectional observation studies in six sub-Saharan African countries. *BMC pregnancy and childbirth* 2018;18(1):346. doi: 10.1186/s12884-018-1972-1
45. Saxena M, Srivastava A, Dwivedi P, et al. Is quality of care during childbirth consistent from admission to discharge? A qualitative study of delivery care in Uttar Pradesh, India. *PloS one* 2018;13(9):e0204607. doi: 10.1371/journal.pone.0204607 [published Online First: 2018/09/28]
46. Hanson C, Munjanja S, Binagwaho A, et al. National policies and care provision in pregnancy and childbirth for twins in Eastern and Southern Africa: A mixed-methods

For peer review only - http://bmjopen.bmj.com/site/about/guidelines.xhtml

multi-country study. *PLoS medicine* 2019;16(2):e1002749. doi: 10.1371/journal.pmed.1002749 [published Online First: 2019/02/20]
47. Marchant T, Beaumont E, Makowiecka K, et al. Coverage and equity of maternal and newborn health care in rural Nigeria, Ethiopia and India. *CMAJ : Canadian Medical Association journal = journal de l'Association medicale canadienne* 2019;191(43):E1179-e88. doi: 10.1503/cmaj.190219 [published Online First: 2019/10/30]
48. Federal Government of Nigeria. Second National Strategic Health Development Plan 2018-2022. 2019;online: http://www.health.gov.ng/doc/NSHDP%20II%20Final.pdf [accessed on 3 February 2020]
49. Network for Improving Quality of Care for Maternal NaCH. Quality of Care in Nigeria. 2019;online: http://www.qualityofcarenetwork.org/country-data/nigeria [accessed on 3 February 2020]
50. Sharma G, Powell-Jackson T, Haldar K, et al. Quality of routine essential care during childbirth: clinical observations of uncomplicated births in Uttar Pradesh, India. *Bulletin of the World Health Organization* 2017;95(6):419-29. doi: 10.2471/blt.16.179291 [published Online First: 2017/06/13]
51. Hanson C, Schellenberg J. Redesigning maternal health services: is centralisation the answer in low-resource settings? *BMJ global health* 2019;4(3):e001488. doi: 10.1136/bmjgh-2019-001488 [published Online First: 2019/07/03]

For peer review only

For peer review only - http://bmjopen.bmj.com/site/about/guidelines.xhtml

Figure legends

Figure 1 Women and newborn's pathway from admission

Figure 2 History taking and initial examination

[FIGURE]

FIGURE NOTE: red line represents the average percent across all five timepoints. For problems experienced during previous pregnancies 10% of observers recorded that they did not know if the birth attendant asked the women. For HIV status 30% of observers recorded that they did not know if it had been checked

Figure 3 Examinations and procedures during first stage of labour

[FIGURE]

FIGURE NOTE: red line represents the average percent across all five timepoints

Figure 4 Care received during second and third stage of labour

[FIGURE]

FIGURE NOTE: red line represents the average percent across all five timepoints

Figure 5 Immediate newborn and postpartum care

[FIGURE]

FIGURE NOTE: red line represents the average across all five timepoints. Administers chlorhexidine and weighs baby was not included in the checklist in the first observation period (timepoint 1).

For peer review only

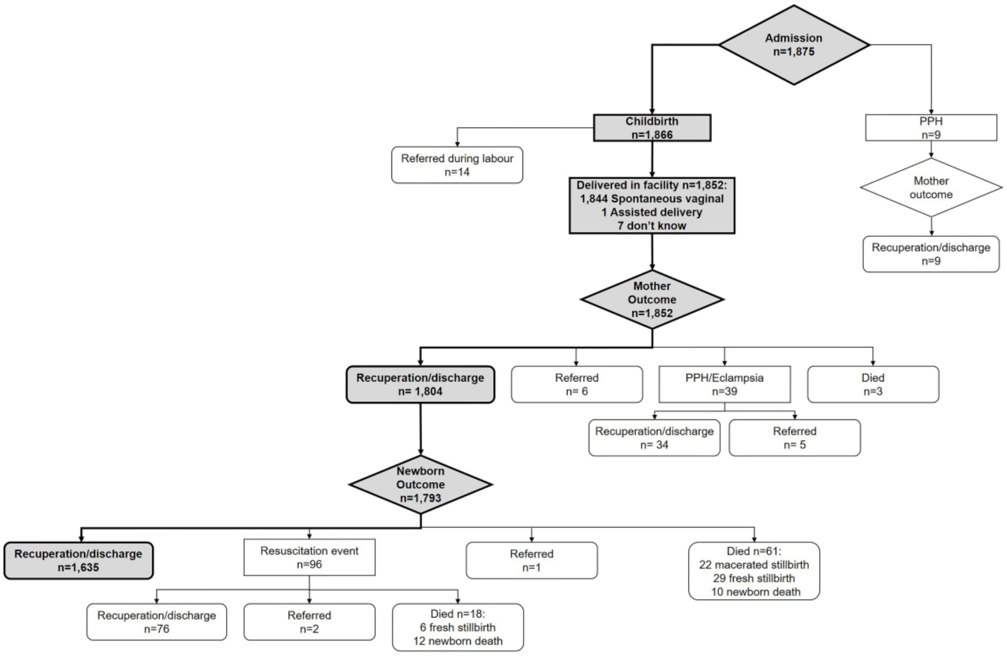

Figure 1 Women and newborn's pathway from admission

437x284mm (300 x 300 DPI)

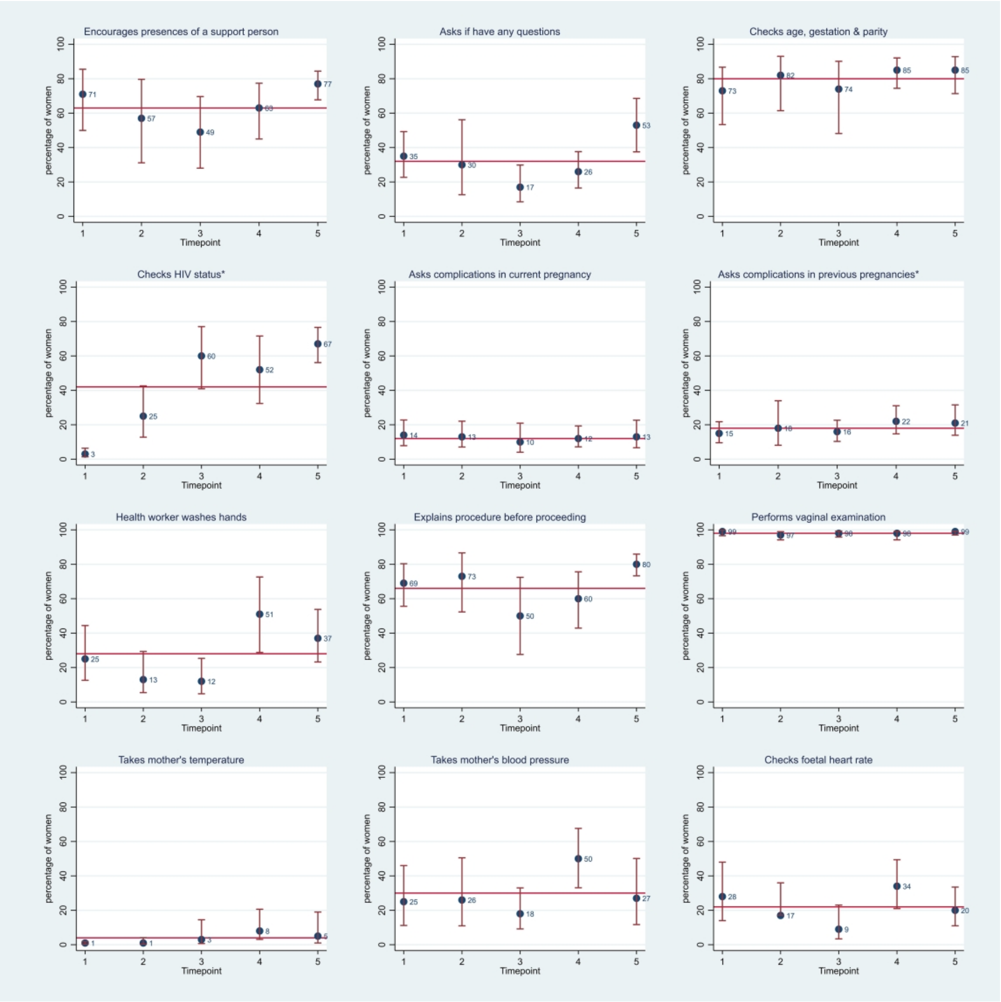

Figure 2 History taking and initial examination
[FIGURE]
FIGURE NOTE: red line represents the average percent across all five timepoints. For problems experienced during previous pregnancies 10% of observers recorded that they did not know if the birth attendant asked the women. For HIV status 30% of observers recorded that they did not know if it had been checked

360x360mm (300 x 300 DPI)

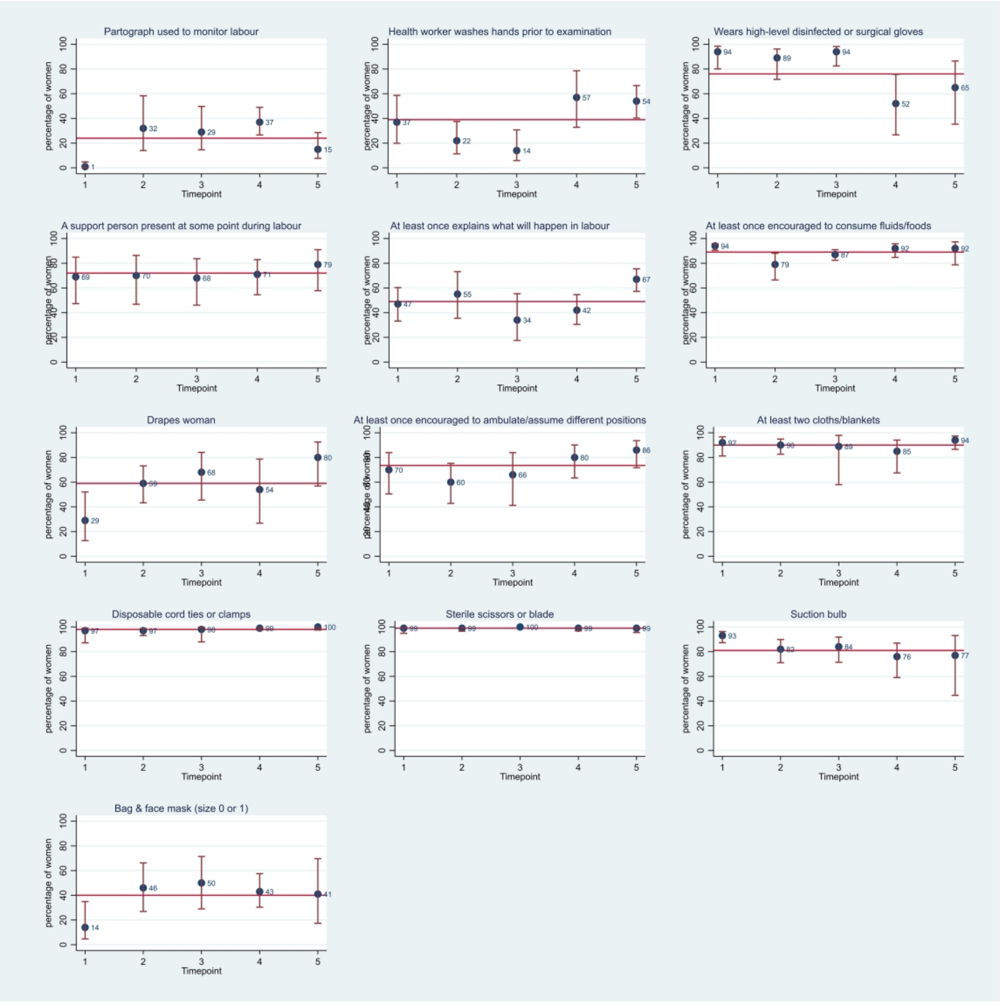

Figure 3 Examinations and procedures during first stage of labour
[FIGURE]
FIGURE NOTE: red line represents the average percent across all five timepoints

360x360mm (300 x 300 DPI)

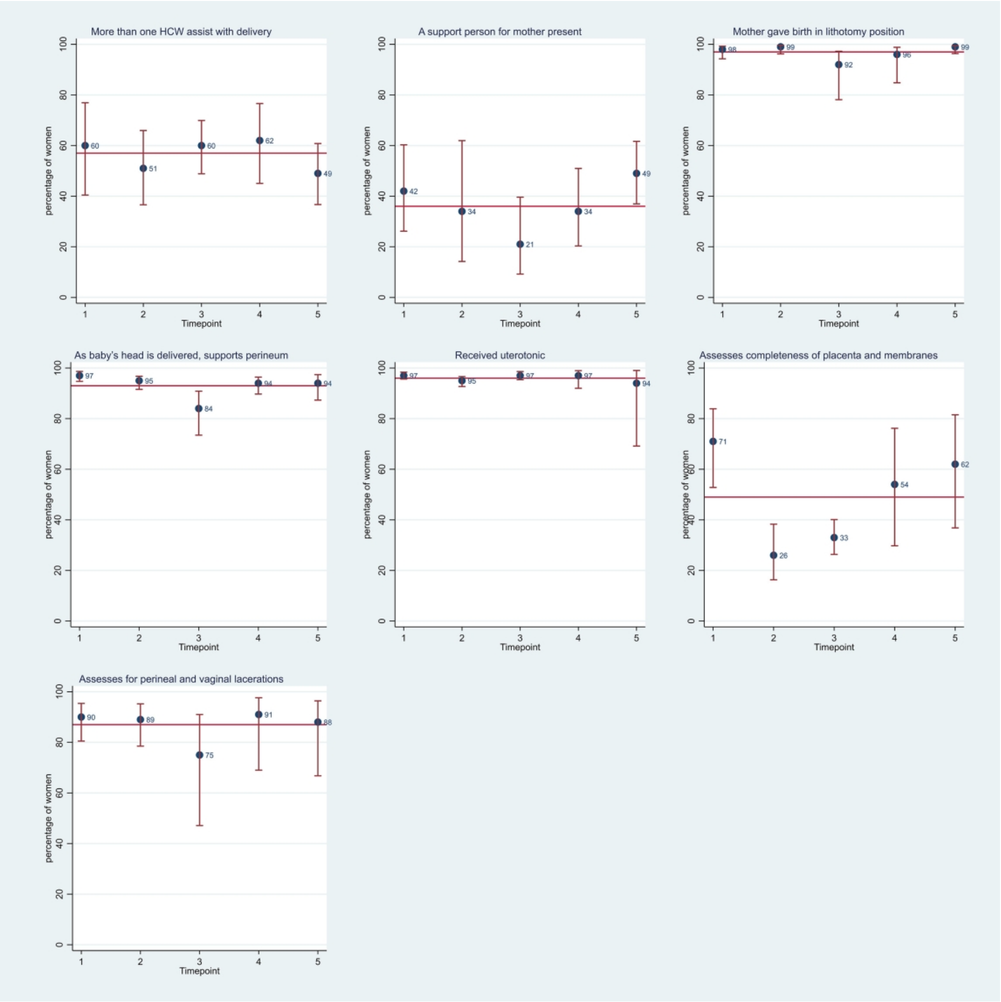

Figure 4 Care received during second and third stage of labour
[FIGURE]
FIGURE NOTE: red line represents the average percent across all five timepoints

240x240mm (300 x 300 DPI)

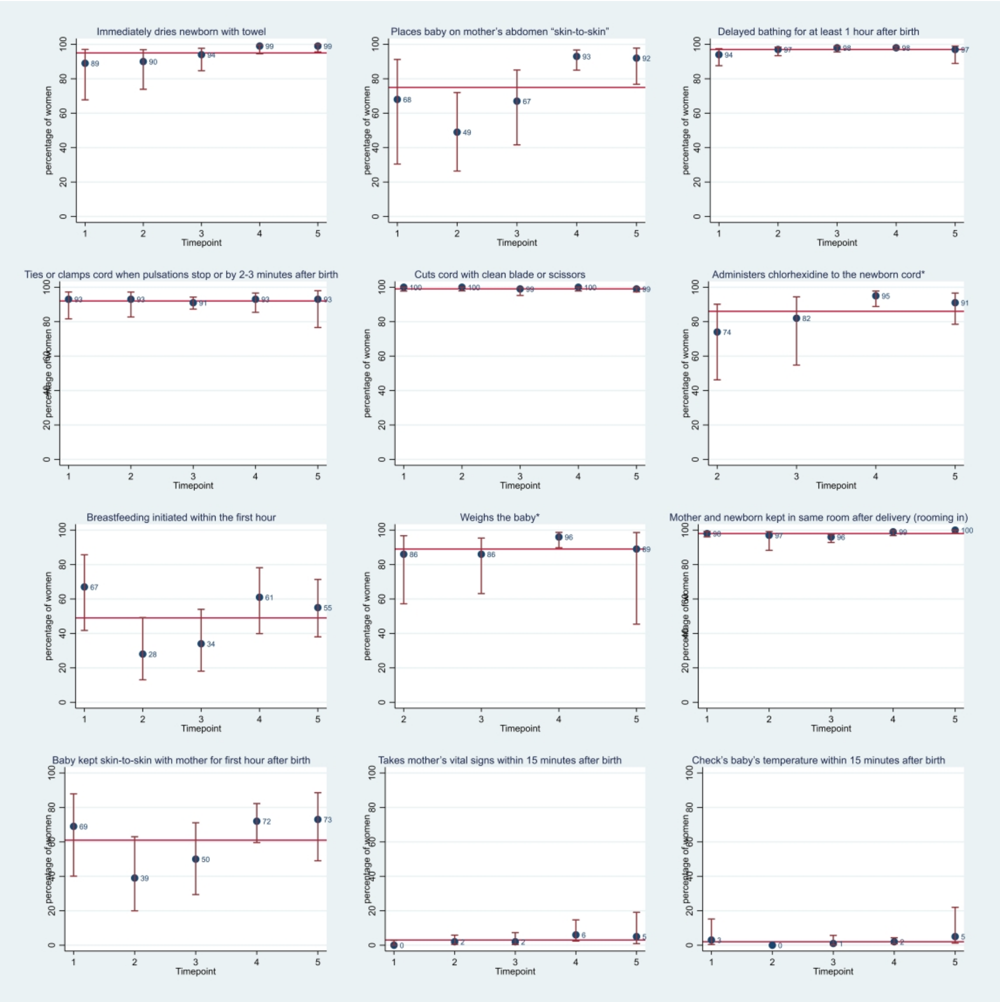

Figure 5 Immediate newborn and postpartum care
[FIGURE]
FIGURE NOTE: red line represents the average across all five timepoints. Administers chlorhexidine and weighs baby was not included in the checklist in the first observation period (timepoint 1).

Supplementary material: full table of results

	Observation period				
	1 (Aug 2016)	2 (Mar 2017)	3 (Aug 2017)	4 (Mar 2018)	5 (Aug 2018)
	Percentage of women (95%CI)				
**History taking & initial assessment**					
Encourages woman to have a support person	70.8% (50.0-85.5)	57.1% (31.1-79.7)	48.6% (28.0-69.6)	62.6% (45.0-77.4)	77.1% (67.8-84.4)
Asks if have any questions	34.8% (22.7-49.3)	30.0% (12.6-56.2)	16.6% (8.5-29.9)	25.7% (16.5-37.6)	53.4% (37.5-68.6)
Checks client card or asks client age, length of pregnancy and parity	73.2% (53.4-86.7)	82.3% (61.4-93.0)	74.4% (48.2-90.1)	85.3% (74.5-92.1)	85.0% (71.4-92.8)
Checks woman's HIV status	3.0% (1.4-6.3)	24.8% (12.8-42.6)	60.4% (40.9-77.0)	52.3% (32.4-71.6)	67.2% (56.1-76.6)
Asks whether experienced any complications during pregnancy	13.7% (7.9-22.8)	12.8% (7.1-22.1)	9.6% (4.0-20.9)	12.0% (7.2-19.3)	12.6% (6.6-22.7)
If had a previous pregnancy, whether experienced any complications during previous pregnancies	14.6% (9.5-21.8)	17.5% (8.1-34.0)	15.5% (10.3-22.6)	21.8% (14.7-31.0)	21.4% (13.9-31.6)
Health worker washes hands	25.3% (12.6-44.4)	13.4% (5.4-29.4)	11.5% (4.8-25.3)	50.9% (28.8-72.6)	37.2% (23.2-53.8)
Explains procedure to woman before proceeding	69.4% (55.6-80.3)	72.7% (52.3-86.6)	50.0% (27.6-72.4)	60.4% (42.9-75.6)	80.4% (73.3-85.9)
Performs vaginal examination	99.1% (96.6-99.8)	97.5% (94.2-998.9)	98.3% (95.8-99.3)	97.6% (94.2-99.0)	98.5% (97.0-99.3)
If performs vaginal exam, wears high-level disinfectant gloves	93.7% (82.8-97.9)	79.7% (48.6-94.2)	94.9% (84.3-98.5)	43.9% (21.3-69.3)	59.2% (35.9-79.0)
Takes temperature	0.6% (0.2-1.9)	1.1% (0.3-4.1)	3.4% (0.7-14.5)	8.3% (3.1-20.6)	4.7% (1.0-19.0)
Takes blood pressure	24.7% (11.2-46.0)	26.2% (11.0-50.5)	18.3% (9.2-33.0)	50.4% (33.1-67.6)	26.7% (11.7-50.0)
Checks foetal heart rate with fetoscope/Doppler/ultrasound	28.0% (14.0-48.0)	17.3% (7.2-36.0)	9.3% (3.4-23.0)	33.8% (21.0-49.4)	19.9% (11.0-33.5)
**First stage of labour: examination and procedures**					
Partograph used to monitor labour	0.6% (0.1-4.8)	32.3% (14.0-58.3)	29.1% (14.6-49.7)	37.1% (26.6-49.0)	15.4% (7.7-28.6)
Washes hands with soap & water or uses antiseptic prior to any examination of woman	37.2% (19.9-58.7)	21.7% (11.3-37.6)	14.3% (5.9-30.7)	57.2% (32.8-78.6)	53.7% (40.3-66.6)

For peer review only

Wears high-level disinfected or surgical gloves	94.2% (80.1-98.5)	88.9% (71.6-96.2)	94.2% (82.5-98.2)	51.6% (26.7-75.7)	65.2% (35.3-86.5)
Median number of vaginal examinations	2 (1-2)	1 (1-2)	2 (1-2)	1 (1-2)	1 (1-2)
Augments labour with oxytocin	14.5% (7.5-26.3)	12.6% (6.5-23.2)	24.9% (17.2-34.5)	18.0% (7.5-37.2)	15.4% (5.9-34.5)
Performs artificial rupture of membranes	7.6% (3.9-14.0)	9.6% (4.3-20.2)	9.0% (3.3-22.5)	3.5% (1.5-7.9)	1.8% (0.4-6.7)
A support person present at some point during labour	69.2% (47.3-84.9)	70.2% (46.8-86.3)	67.7% (46.1-83.7)	70.7% (54.5-82.9)	78.9% (57.8-91.0)
At least once explains what will happen in labour	46.5% (33.2-60.3)	55.1% (35.5-73.2)	33.9% (17.5-55.3)	42.1% (30.5-54.6)	67.0% (57.2-75.5)
At least encouraged to consume fluids/foods during labour	93.6% (90.4-95.8)	79.3% (66.5-88.1)	87.3% (82.4-91.0)	91.9% (84.7-95.8)	92.1% (78.7-97.3)
Drapes woman	28.5% (12.7-52.1)	59.1% (43.3-73.2)	67.7% (45.5-84.1)	53.7% (26.8-78.7)	80.2% (56.9-92.5)
At least once encourages woman to ambulate and assume different positions in labour	69.8% (50.5-83.9)	60.1% (42.8-75.2)	65.6% (41.2-83.9)	79.9% (63.4-90.1)	85.9% (71.7-93.6)
**First stage of labour: equipment and supplies laid out in preparation for delivery**					
At least two cloths/blankets	91.9% (81.2-96.7)	90.4% (82.7-94.9)	88.9% (58.0-97.9)	85.2% (67.5-94.1)	93.8% (86.6-97.3)
Disposable cord ties or clamps	96.5% (87.1-99.1)	97.0% (93.1-98.7)	98.4% (87.9-99.8)	99.3% (97.4-99.8)	99.6% (97.5-99.9)
Sterile scissors or blade	98.8% (94.9-99.8)	99.0% (96.6-99.7)	100%	98.9% (96.8-99.7)	98.7% (95.5-99.6)
Suction bulb	93.0% (87.3-96.3)	82.3% (71.0-89.9)	84.1% (71.4-91.8)	75.6% (59.1-87.0)	76.7% (44.6-93.0)
Bag & face mask (size 0 or 1)	14.0% (4.7-35.0)	46.0% (26.9-66.2)	50.3% (29.0-71.4)	43.5% (30.4-57.6)	41.0% (17.3-69.7)
**Second and third stage of labour**					
More than one HCW assist with delivery	60.1% (40.4-77.0)	51.4% (36.6-66.0)	59.8% (48.9-69.9)	62.1% (45.1-76.6)	48.7% (36.7-60.8)
A support person for mother present	42.3% (26.2-60.3)	34.2% (14.2-62.0)	20.5% (9.2-39.6)	34.0% (20.3-51.0)	49.3% (37.0-61.7)
Performs episiotomy	1.5% (0.4-5.4)	0.8% (0.2-4.4)	0.6% (0.1-2.4)	2.4% (1.0-5.8)	0.3% (0-2.6)
Mother gave birth in lithotomy position	97.9% (94.3-99.3)	98.9% (96.2-99.7)	91.9% (78.1-97.3)	95.6% (84.8-98.8)	98.5% (96.4-99.4)

For peer review only

As baby's head is delivered, supports perineum	97.3% (94.7-98.7)	94.7% (91.6-96.7)	84.0% (73.5-90.9)	93.9% (89.7-96.4)	94.1% (87.3-97.4)
Checks for another baby prior to giving the uterotonic	56.8% (31.7-78.9)	50.8% (33.6-67.9)	52.8% (37.9-67.2)	81.2% (59.5-92.7)	83.0% (67.9-91.9)
Received uterotonic	97.3% (95.6-98.4)	95.0% (92.7-96.6)	97.5% (95.4-98.6)	97.1% (92.0-99.0)	93.8% (69.2-99.0)
If uterotonic received, timing of administration					
At delivery	0.3% (0-2.4)	0.3% (0-2.7)	0.3% (0.1-1.3)	1.8% (0.6-5.0)	0
Within 1 minute of delivery	23.0% (9.4-46.4)	8.8% (2.7-25.0)	10.9% (5.8-19.5)	11.1% (4.9-23.1)	16.6% (9.0-28.6)
Within 3 minutes of delivery	28.5% (22.7-35.1)	30.7% (19.7-44.5)	26.1% (18.4-35.6)	37.0% (29.4-45.4)	51.3% (42.1-60.4)
More than 3 minutes	47.6% (31.6-64.1)	60.2% (41.1-76.7)	62.5% (54.0-70.3)	50.1% (37.9-62.4)	32.2% (25.3-39.9)
Applies traction to the cord while applying suprapubic counter traction	87.3% (80.8-91.8)	81.9% (61.9-92.7)	80.6% (70.5-87.9)	85.3% (67.3-94.3)	91.2% (71.7-97.7)
If skilled (doctor, nurse, midwife) birth assistant, applies traction to the cord while applying suprapubic counter traction	100%	95.4% (63.0-99.6)	88.7% (81.9-93.2)	82.4% (77.4-86.4)	90.5% (48.1-99.0)
If CHEW birth assistant, applies traction to the cord while applying suprapubic counter traction	87.6% (78.8-93.0)	80.9% (58.9-92.6)	81.9% (67.4-90.8)	85.7% (54.9-96.7)	88.9% (66.8-97.0)
If unskilled birth assistant, applies traction to the cord while applying suprapubic counter traction	86.1% (78.1-91.5)	77.8% (57.5-90.0)	77.0% (63.8-86.4)	85.2% (65.2-94.7)	93.0% (77.1-98.1)
Performs uterine massage immediately following the delivery of the placenta	73.7% (63.7-81.7)	78.6% (60.7-89.8)	63.8% (52.6-73.6)	79.0% (63.6-89.0)	77.4% (63.4-87.2)
Assesses completeness of placenta and membranes	70.7% (52.8-83.9)	25.8% (16.3-38.3)	32.9% (26.4-40.1)	53.8% (29.8-76.2)	61.6% (36.8-81.5)
Assesses for perineal and vaginal lacerations	90.2% (80.5-95.4)	89.4% (78.5-95.2)	75.0% (47.1-91.0)	90.5% (69.0-97.6)	88.0% (66.8-96.4)
**Immediate newborn and postpartum care**					
Immediately dries newborn with towel	89.3% (67.8-97.1)	90.5% (73.9-96.9)	93.9% (84.7-97.7)	98.7% (94.5-99.7)	99.4% (95.6-99.9)
Places baby on mother's abdomen "skin-to-skin"	68.1% (30.5-91.2)	49.0% (26.4-72.0)	66.9% (41.6-85.1)	92.8% (85.0-96.7)	92.3% (76.8-97.8)
Delayed bathing until at least 1 hour after birth	94.3% (87.6-97.5)	96.7% (93.4-98.4)	97.9% (95.6-99.0)	98.1% (96.9-99.0)	96.5% (88.9-99.0)

For peer review only - http://bmjopen.bmj.com/site/about/guidelines.xhtml

Ties or clamps cord when pulsations stop or by 2-3 minutes after birth	92.6% (81.6-97.3)	92.8% (82.7-97.2)	91.4% (87.3-94.3)	92.8% (85.4-96.6)	92.7% (76.6-98.0)
Cuts cord with clean blade or scissors	99.7% (97.8-100)	99.7% (97.9-100)	98.8% (95.2-99.7)	99.7% (97.8-100)	99.4% (97.3-99.9)
Administers chlorhexidine to the newborn cord	Not measured	73.7% (46.0-90.2)	81.9% (54.6-94.5)	95.0% (88.8-97.8)	91.1% (78.4-96.6)
Breastfeeding initiated within the first hour	67.5% (41.8-85.7)	27.6% (13.1-49.2)	33.7% (18.1-54.0)	60.6% (39.9-78.2)	55.3% (38.0-71.4)
Check's baby's temperature within 15 minutes after birth	2.7% (0.4-15.2)	0%	0.6% (0.1-5.7)	1.9% (0.8-4.3)	5.4% (1.2-22.0)
Takes mother's vital signs 15 minutes after birth	0.3% (0-2.8)	1.8% (0.5-5.9)	1.8% (0.4-7.3)	6.2% (2.4-14.7)	4.5% (0.9-19.1)
Weighs the baby	Not measured	86.3% (57.2-96.7)	85.6% (63.1-95.4)	96.3% (89.8-98.7)	88.5% (45.4-98.6)
Mother and newborn kept in same room after delivery (rooming in)	98.3% (96.1-99.3)	96.7% (88.3-99.1)	95.7% (92.8-97.5)	98.9% (96.8-99.7)	99.7% (98.4-98.8)
Baby kept skin-to-skin with mother for first hour after birth	68.8% (40.1-87.9)	39.5% (20.0-63.0)	50.3% (29.4-71.1)	72.3% (59.6-82.3)	73.2% (49.0-88.6)
Provides tetracycline ointment prophylaxis	0%	0.3% (0-2.9)	0.9% (0.2-4.6)	10.4% (4.4-22.4)	4.8% (1.5-13.9)
Administers Vitamin K to newborn	0%	0.3% (0-2.1)	0%	0%	0%
Administers chlorhexidine to the newborn cord	0%	73.7% (46.2-90.1)	81.9% (54.8-94.4)	95.0% (88.8-97.8)	91.1% (78.5-96.6)
If mother identified as HIV positive, administers ARV to newborn	100%	75.0% (20.5-97.2)	0%	0%	25.0% (2.3-82.5)
Administers antibiotics to mother	1.0% (0.3-3.6)	1.2% (0.5-2.7)	0%	1.8% (0.9-3.5)	6.0% (1.1-27.0)

For peer review only

Reporting checklist for cross sectional study.

Based on the STROBE cross sectional guidelines.

Instructions to authors

Complete this checklist by entering the page numbers from your manuscript where readers will find each of the items listed below.

Your article may not currently address all the items on the checklist. Please modify your text to include the missing information. If you are certain that an item does not apply, please write "n/a" and provide a short explanation.

Upload your completed checklist as an extra file when you submit to a journal.

In your methods section, say that you used the STROBE cross sectionalreporting guidelines, and cite them as:

von Elm E, Altman DG, Egger M, Pocock SJ, Gotzsche PC, Vandenbroucke JP. The Strengthening the Reporting of Observational Studies in Epidemiology (STROBE) Statement: guidelines for reporting observational studies.

		Reporting Item	Page Number
**Title and abstract**			
Title	#1a	Indicate the study’s design with a commonly used term in the title or the abstract	1

For peer review only

Abstract	#1b	Provide in the abstract an informative and balanced summary of what was done and what was found	2
**Introduction**			
Background / rationale	#2	Explain the scientific background and rationale for the investigation being reported	4
Objectives	#3	State specific objectives, including any prespecified hypotheses	4
**Methods**			
Study design	#4	Present key elements of study design early in the paper	4
Setting	#5	Describe the setting, locations, and relevant dates, including periods of recruitment, exposure, follow-up, and data collection	4
Eligibility criteria	#6a	Give the eligibility criteria, and the sources and methods of selection of participants.	5-7
	#7	Clearly define all outcomes, exposures, predictors, potential confounders, and effect modifiers. Give diagnostic criteria, if applicable	6-7
Data sources / measurement	#8	For each variable of interest give sources of data and details of methods of assessment (measurement). Describe comparability of assessment methods if there is more than one group. Give information separately for for exposed and unexposed groups if applicable.	5

For peer review only

Bias	#9	Describe any efforts to address potential sources of bias	n/a
Study size	#10	Explain how the study size was arrived at	5
Quantitative variables	#11	Explain how quantitative variables were handled in the analyses. If applicable, describe which groupings were chosen, and why	7
Statistical methods	#12a	Describe all statistical methods, including those used to control for confounding	7
Statistical methods	#12b	Describe any methods used to examine subgroups and interactions	n/a
Statistical methods	#12c	Explain how missing data were addressed	n/a
Statistical methods	#12d	If applicable, describe analytical methods taking account of sampling strategy	7
Statistical methods	#12e	Describe any sensitivity analyses	n/a
**Results**			
Participants	#13a	Report numbers of individuals at each stage of study—eg numbers potentially eligible, examined for eligibility, confirmed eligible, included in the study, completing follow-up, and analysed. Give information separately for for exposed and unexposed groups if applicable.	8-10
Participants	#13b	Give reasons for non-participation at each stage	5

For peer review only

Participants	#13c	Consider use of a flow diagram	8
Descriptive data	#14a	Give characteristics of study participants (eg demographic, clinical, social) and information on exposures and potential confounders. Give information separately for exposed and unexposed groups if applicable.	8
Descriptive data	#14b	Indicate number of participants with missing data for each variable of interest	9-10
Outcome data	#15	Report numbers of outcome events or summary measures. Give information separately for exposed and unexposed groups if applicable.	10-12
Main results	#16a	Give unadjusted estimates and, if applicable, confounder-adjusted estimates and their precision (eg, 95% confidence interval). Make clear which confounders were adjusted for and why they were included	10-12
Main results	#16b	Report category boundaries when continuous variables were categorized	n/a
Main results	#16c	If relevant, consider translating estimates of relative risk into absolute risk for a meaningful time period	n/a
Other analyses	#17	Report other analyses done—e.g., analyses of subgroups and interactions, and sensitivity analyses	12
**Discussion**			
Key results	#18	Summarise key results with reference to study objectives	12-13

For peer review only

Limitations	#19	Discuss limitations of the study, taking into account sources of potential bias or imprecision. Discuss both direction and magnitude of any potential bias.	14
Interpretation	#20	Give a cautious overall interpretation considering objectives, limitations, multiplicity of analyses, results from similar studies, and other relevant evidence.	14-15
Generalisability	#21	Discuss the generalisability (external validity) of the study results	14

Other Information

Funding	#22	Give the source of funding and the role of the funders for the present study and, if applicable, for the original study on which the present article is based	16

None The STROBE checklist is distributed under the terms of the Creative Commons Attribution License CC-BY. This checklist can be completed online using https://www.goodreports.org/, a tool made by the EQUATOR Network in collaboration with Penelope.ai

For peer review only

BMJ Open

The provision of Eessential evidence-based interventions during facility-based childbirth: cross sectional clinical observations of births in northeast Nigeria

Journal:	*BMJ Open*
Manuscript ID	bmjopen-2020-037625.R1
Article Type:	Original research
Date Submitted by the Author:	29-Jun-2020
Complete List of Authors:	Exley, Josephine; London School of Hygiene and Tropical Medicine Faculty of Infectious and Tropical Diseases, Department of Disease Control<br>Hanson, Claudia; Karolinska Institute, Public Health Sciences; London School of Hygiene and Tropical Medicine Faculty of Infectious and Tropical Diseases, Dept of Disease Control<br>Umar, Nasir; London School of Hygiene and Tropical Medicine Faculty of Infectious and Tropical Diseases, Disease Control<br>Willey, Barbara; London School of Hygiene and Tropical Medicine, Disease Control Department; London School of Hygiene and Tropical Medicine, Dept of Global Health and Development<br>Shuaibu, Abdulrahman; Gombe State Primary Health Care Development Agency<br>Marchant, Tanya; London Sch Hyg , Disease Control
**Primary Subject Heading**:	Health services research
Secondary Subject Heading:	Evidence based practice, Obstetrics and gynaecology
Keywords:	Maternal medicine < OBSTETRICS, Quality in health care < HEALTH SERVICES ADMINISTRATION & MANAGEMENT, PRIMARY CARE

SCHOLARONE™
Manuscripts

I, the Submitting Author has the right to grant and does grant on behalf of all authors of the Work (as defined in the below author licence), an exclusive licence and/or a non-exclusive licence for contributions from authors who are: i) UK Crown employees; ii) where BMJ has agreed a CC-BY licence shall apply, and/or iii) in accordance with the terms applicable for US Federal Government officers or employees acting as part of their official duties; on a worldwide, perpetual, irrevocable, royalty-free basis to BMJ Publishing Group Ltd ("BMJ") its licensees and where the relevant Journal is co-owned by BMJ to the co-owners of the Journal, to publish the Work in this journal and any other BMJ products and to exploit all rights, as set out in our licence.

The Submitting Author accepts and understands that any supply made under these terms is made by BMJ to the Submitting Author unless you are acting as an employee on behalf of your employer or a postgraduate student of an affiliated institution which is paying any applicable article publishing charge ("APC") for Open Access articles. Where the Submitting Author wishes to make the Work available on an Open Access basis (and intends to pay the relevant APC), the terms of reuse of such Open Access shall be governed by a Creative Commons licence – details of these licences and which Creative Commons licence will apply to this Work are set out in our licence referred to above.

Other than as permitted in any relevant BMJ Author's Self Archiving Policies, I confirm this Work has not been accepted for publication elsewhere, is not being considered for publication elsewhere and does not duplicate material already published. I confirm all authors consent to publication of this Work and authorise the granting of this licence.

For peer review only

The provision of essential evidence-based interventions during facility-based childbirth: cross sectional observations of births in northeast Nigeria

Josephine Exley[1], Claudia Hanson[1,2], Nasir Umar[1], Barbara Willey[3], Abdulrahman Shuaibu[4], Tanya Marchant[1*]

[1] Department of Disease Control, London School of Hygiene & Tropical Medicine, UK

[2] Department of Public Health Sciences–Global Health, Karolinska Institutet, Stockholm, Sweden

[3] Department of Infectious Disease Epidemiology, London School of Hygiene & Tropical Medicine, UK

[4] The Executive Secretary, Gombe State Primary Health Care Development Agency, Nigeria

Correspondence to:
Josephine Exley
LSHTM
Keppel Street
London
WC1E 7HT
United Kingdom
Josephine.Exley@lshtm.ac.uk

Word Count: 4,764

For peer review only

For peer review only - http://bmjopen.bmj.com/site/about/guidelines.xhtml

Abstract

Objectives:

To measure the provision of evidence-based preventive and promotive interventions to women, and subsequently their newborns, during childbirth in a high-mortality setting.

Design and participants:

Cross-sectional observations of care provided to women, and their newborns during the intra- and immediate postpartum period using a standardised checklist capturing healthcare worker behaviours regarding lifesaving and respectful care.

Setting:

Ten primary healthcare facilities in Gombe State, northeast Nigeria. The northeast region of Nigeria has some of the highest maternal and newborn death rates globally.

Main outcome measures:

Data on 50 measures of internationally recommended evidence-based interventions and good practice.

Results:

1,875 women were admitted to a health facility during the observation period; of these, 1,804 gave birth in the facility and did not experience an adverse event or death. Many clinical interventions around the time of birth were routinely implemented, including provision of uterotonic (96% [95%CI 93-98]), whereas risk-assessment measures, such as history-taking or checking vital signs were rarely completed: just 2% (95%CI 2-7) of women had temperature taken and 12% (95%CI 9-16) were asked about complications during pregnancy.

Conclusions:

The majority of women did not receive the recommended routine processes of childbirth care they and their newborns needed to benefit from their choice to deliver in a health facility. In particular, few benefited from even basic risk-assessments, leading to missed opportunities to identify risks. To continue with the recommendation of childbirth care in primary healthcare in high mortality settings like Gombe it is crucial that birth attendant capacity, capability and prioritisation processes are addressed.

For peer review only

Strengths and limitations of this study

- This study has a large sample size; over 1,850 women were observed across five rounds of data collection.
- The relative consistency overtime and low levels of implementation of many measures suggest that any impact of being observed was minimal.
- Observers received training, used a structured checklist and were overseen by a clinical supervisor to improve the reliability of observations.
- The study protocol prioritised observation of events closest to birth, as such 40% of women were not observed during the first stage of labour.
- This study was completed in the 10 primary healthcare facilities with the highest volume of births in Gombe State and therefore results are not representative of all facilities.

For peer review only

Introduction

Global efforts to reduce preventable maternal and newborn mortality have focused on skilled attendance at birth. This has resulted in marked increases in coverage of births with a skilled birth attendant, mainly operationalised through childbirth in facilities. However, these increases have not been accompanied by the anticipated improvements in maternal and newborn outcomes in many low and middle income countries [1-4], prompting a closer examination of the quality of care provided [5-7]. A growing body of evidence from low income settings highlights low provider skills and limited facility capability to provide good-quality care at birth [1 8-10]. A recent review found large declines in the proportion of individuals estimated to have skilled birth attendance when some measure of quality was taken into account [11].

Good quality of care includes the timely and appropriate use of evidence-based clinical and non-clinical interventions that are acceptable to women [12-14]. The World Health Organisation (WHO) has developed evidence-based guidelines around intrapartum care, which have the potential to support healthcare providers to identify gaps in the quality of care and improve the provision and experience of care [15]. A continuing challenge, however, is that detailed evidence on the extent to which the recommended interventions are practised during routine childbirth in health facilities in low-income countries is scarce [1 16]. Available studies have predominantly focused on readiness for quality such as availability of drugs, supplies and other inputs, while neglecting processes of care [16]. In part, this is because these data are made available through surveys similar to the Service Provision Assessments [17], or through routine health information systems - information sources that include very little process quality data. Household surveys, including the Demographic and Health Survey and the Multiple Indicator Cluster Survey, do not collect extensive data on the content of care during childbirth, in part because evidence suggests women's self-reports have low validity [18 19]. This lack of data on coverage of evidence-based care during childbirth impedes decision making around possible solutions. Although not a large-scale measurement solution, observations provide useful insight in to the behaviours of healthcare workers and could support strategies to improve care.

In this study of birth observations, we aimed to examine the maternal and newborn outcomes experienced by all women admitted for childbirth and postpartum haemorrhage in a sample of primary healthcare facilities in Gombe State, Nigeria. For women who had an uncomplicated labour, we evaluated the provision of evidence-based care provided to women, and subsequently their newborns, from initial assessment up to one hour postpartum.

Methods

We conducted direct observations of childbirth care in 10 primary healthcare facilities, in Gombe State, Nigeria, approximately every six months over a two-year period between June 2016 and August 2018.

Study setting

Gombe State is one of six states in northeast Nigeria, it has an area of 20,265km^2 and a population of 2,857,042 [20]. Over 80% of the population live in rural areas and are reliant on subsistence farming as their primary source of income [21]. The northeast region of Nigeria has some of the highest maternal and newborn death rates globally, estimated at 1,549 per 100,000 live births in 2015 and 33 per 1,000 live births in 2017, respectively [22 23].

Access to maternal healthcare services is relatively low in Gombe State. In 2018, 46% of women in the State reported at least one antenatal care visit from a doctor, nurse, midwife or

For peer review only - http://bmjopen.bmj.com/site/about/guidelines.xhtml

nurse/midwife and 28% delivered in a health facility [24]. Over 70% of facility deliveries, in 2018, took place in a rural primary healthcare facility [25]. Recent work in Gombe on the drivers of attending a facility for childbirth found that health system conditions including availability of staff, drugs and supply, and a clean environment had the biggest influence on respondents' decision around where to give birth [26].

Healthcare is predominantly delivered via a network of rural primary healthcare clinics run by the Gombe State Primary Healthcare Development Agency (GSPHCDA). In 2017, 460 primary healthcare clinics and 26 referral facilities provided childbirth services [27]. In primary healthcare facilities care is typically delivered by lower cadres of healthcare workers, for example, community health extension workers (CHEWS), junior CHEWS and health officers [28 29]. In response to the shortage and uneven distribution of healthcare workers, under its task-shifting and task-sharing policy for essential healthcare services, Nigeria classifies CHEWs as skilled birth attendants [30].

Primary healthcare facilities in Gombe are poorly resourced, often lacking essential supplies and commodities to provide basic maternal and newborn health care [31-33]. Led by the Gombe State Primary Healthcare Development Agency, since 2016 intense NGO activity has been ongoing in 57 primary healthcare facilities across Gombe State, aimed at increasing the quality of care [34 35]. Interventions include training of CHEWs in all aspects of skilled birth attendance and basic emergency obstetric care, and improving the supply of essential maternal and newborn health commodities [36]. These facilities provide basic emergency obstetric and newborn care. Emergency care and complicated cases from these health facilities are referred to referral facilities. None of the 57 primary healthcare facilities have a medical doctor, 4% have at least one nurse and 19% have at least one midwife.[37]

Sampling methods have been described in detail elsewhere [19 32]. Briefly, in November 2015, 10 primary healthcare facilities were selected from the 57 facilities for an in-depth assessment of quality of care. To achieve a sufficiently large number of observations and minimise the duration of data collection, the 10 primary healthcare facilities with the highest number of births in the preceding six months, as recorded in the maternity register, were purposively selected. The mean number of births per month in the 10 primary healthcare facilities was 15.7 (standard deviation [sd] 12.0), compared to 4.3 (sd 6.3) births per facility per month across Gombe State as a whole [19].

Data collection

Five rounds of data collection took place over the two-year study period. Each round lasted three weeks, during which observers aimed to collect data from a total of around 350 women. Two trained female observers (local midwives, not employed by the facility) and one clinical supervisor were assigned to each facility. Observers worked in 8- or 12-hour shifts to provide near continual data collection during the period. Depending on the observation team's work schedule, the first point of contact for any observation may have been during initial assessment of a newly admitted pregnant woman or at a later stage of labour. Observers aimed to observe all women who were admitted irrespective of the cadre of the attending healthcare worker, but they prioritised observing women during the second and third stage of labour and immediately postpartum rather than observing women earlier in the process. Observers stayed continuously with women from the first point of contact until the first hour after birth. The healthcare worker observed may have been different at different timepoints in the same facility. The clinical supervisor was always available onsite but not present in the delivery room.

For peer review only - http://bmjopen.bmj.com/site/about/guidelines.xhtml

A structured clinical observation checklist, administered on a Lenovo A3300 tablet using CSPro version 7.0 (United States Census Bureau and ICF Macro, Suitland, MD, USA), was used to record the processes of care and birth attendant-client interactions and client characteristics. The content of the checklist was developed from the USAID-funded Maternal and Child Health Integrated Program's tool for observing vaginal births and the following complications: postpartum haemorrhage, pre-eclampsia/eclampsia and newborn asphyxia [38]. The checklist was piloted and modified to the Gombe context.

All women attending the facility in active labour or experiencing postpartum haemorrhage were invited to participate at the time of admission. All potential participants were provided with a study information sheet and a consent form in English and Hausa. Taking care to include any support persons accompanying potential participants, the observer read the information sheet, explained the purpose of the study, the risks and benefits of participating and answered questions before seeking written consent from the woman and verbal consent from the healthcare worker attending. Women who were not able to write their name were asked to provide a thumb print on the consent form. Participation was voluntary and participants were free to withdraw at any time.

Before each round of data collection, observers underwent four days of training on how to conduct unobtrusive observations, the safety and confidentiality protocols and how to ensure consistency of rating between observers. Throughout the observation period, clinic supervisors conducted spot checks of observers and data to provide ongoing quality assurance.

Observers were required to prioritise the safety of the mother and newborn; protocols were established on the actions to take during any life-threatening events. This included immediately stopping the observation activity and calling for the clinical supervisor who could advise the attending healthcare worker. A formal report detailing any actions and decisions made, was made available to the Executive Secretary of the GSPHCDA. Where data collection was stopped, observations were excluded from the study.

Defining provision of evidence-based care

For this analysis, the content of the clinical observation checklist was mapped against current recommendations for high quality mother and newborn care [13 15 39-42]. Fifty measures were identified (Box 1), grouped into four organising categories based on the stage of childbirth: (1) initial assessment; (2) first stage of labour; (3) second and third stage of labour; (4) immediate newborn and postpartum care.

Box 1 Measures of evidence based childbirth care included in this analysis

**History taking and initial assessment**
• Checks client card or asks client her age, length of pregnancy, and parity
• Asks whether woman has experienced any complications during current pregnancy
• If woman has had any previous pregnancies, asks about complications during previous pregnancies
• Checks client card or asks client her HIV status
• Washes his/her hands with soap and water or uses disinfectant before initial any examination
• Takes temperature
• Takes blood pressure
• Checks foetal heart rate with fetoscope/doppler/ultrasound
• Performs vaginal examination

For peer review only - http://bmjopen.bmj.com/site/about/guidelines.xhtml

• Encourages the women to have a support person present during labour and birth<br>• Explains procedures to woman (support person) before proceeding<br>• Asks women (and support person) if she has any questions
**First stage of labour**
• Partograph used to monitor labour<br>• Washes his/her hands with soap and water or uses antiseptic prior to any examination of woman<br>• Wears high-level disinfected or sterile surgical gloves<br>• A support person (or companion) for mother is present at some point during labour<br>• At least once, explains what will happen in labour to woman (support person)<br>• At least once, encourages woman to consume fluids/food during labour<br>• Drapes woman (one drape under buttocks, one over abdomen)<br>• At least once, encourages/assists woman to ambulate and assume different positions during labour<br>• Following items of equipment laid out in preparation for birth:<br>○ Newborn face mask size 0 or 1 and self-inflating ventilation bag (250 or 500 mL)<br>○ Suction bulb<br>○ Disposable cord ties or clamps<br>○ Sterile scissors or blade<br>○ At least two cloths/blankets (one to dry; one to cover)
**Second & third stage of labour**
• Assisted by more than one healthcare worker at one point during labour<br>• Mother gave birth in lithotomy position (on back)<br>• As baby's head is delivered, supports perineum<br>• Administers uterotonic<br>• Timing of uterotonic<br>• If care provided by a skilled birth attendant, applies traction to the cord while applying suprapubic counter traction<br>• Assesses completeness of the placenta and membranes<br>• Assesses for perineal and vaginal lacerations<br>• A support person (companion) for mother is present at birth

For peer review only

Immediate newborn and postpartum care
• Drying baby immediately after birth with towel
• Baby placed skin-to-skin with mother
• Bathing delayed for at least one hour
• Ties or clamps cord when pulsations stop, or by 2-3 minutes after birth (not immediately after birth)
• Cuts cord with clean blade or clean scissors
• Breastfeeding initiated within first hour
• Takes mother's vital signs within the first 15 minutes after birth
• Administers antibiotics to mother postpartum
• Checks baby's temperature within the first 15 minutes after birth
• Baby weighed
• Mother and newborn kept in same room after birth (rooming-in)
• Baby kept skin-to-skin for first hour
• Administers Vitamin K to newborn
• Provides tetracycline eye ointment prophylaxis
• Administers chlorhexidine to the newborn cord
• If the mother is HIV positive, administers ARVs to newborn

Inclusion criteria

Data from the five data collection periods were combined into a single dataset. Observations were excluded from the dataset if the woman's outcome was not recorded. For all women observed, we mapped the different pathways from admission to the facility (childbirth or postpartum haemorrhage event) to their outcome. For women who experienced an uncomplicated labour the outcome of their baby was also mapped. An uncomplicated labour was defined as a woman who was sent to the ward for recuperation or discharged home after birth and who did not experience an adverse event to her own health (referral, postpartum haemorrhage or pre-eclampsia/eclampsia) or death.

For the analysis of the provision of essential evidence based care, our population of interest was women with an uncomplicated labour and detailed information on their care and that of their newborn are included here. Women who were admitted but experienced an adverse event or death were excluded from the analysis because of their individual medical needs. For measures related to newborn care the analysis was further restricted to newborns recorded as being alive and who did not require resuscitation care or were not referred to another facility.

Analysis

For each measure, percent frequencies and 95% confidence intervals were calculated, adjusted for clustering by primary healthcare facility and stratified by time point using the svyset and svy commands in STATA version 15.1 (StataCorp, 2017, College Station, TX). Results are presented graphically by time point to highlight any variability and the average across all five time points is presented in the text.

Ethical approval

Ethical approval for this study were obtained from the London School of Hygiene & Tropical Medicine (reference 14091) and the Health Research Ethics Committees for Nigeria (reference NHREC/01/01/2007) and Gombe State (reference ADM/S/658/Vol. II/66).

For peer review only - http://bmjopen.bmj.com/site/about/guidelines.xhtml

Patient and Public Involvement

Patients and the public were not involved in the design, conduct, reporting or dissemination plans of our research. Observations were recorded in English and pre-testing completed in health facilities by staff.

Results

In total 1,875 women were admitted to a facility during the five observations periods. The median age of the women was 24 years (interquartile range [IQR] 20–29) and for 19% it was their first birth: median parity 2 (IQR 1–5). The median gestational age of women on admission was 39 weeks (IQR 38–39); 6% of women had a gestational age of less than 37 weeks and for 21% of women gestational age was not recorded on their client card and/or they did not know. At the start of the observation period, 11% of women were attended by a skilled birth attendant (doctor, midwife, nurse), 39% by a CHEW or junior CHEW and 50% by an 'other' birth attendant. 'Other' included environmental health officers/technicians/assistants (43%), health attendant/assistant (43%), traditional birth attendants (4%), community health officer (1%) and other (9%).

We first present the outcomes for all 1,875 women who were admitted to the facility, followed by the coverage of the provision of evidence based care measures, outlined in Box 1, for the 1,804 women who had an uncomplicated labour and the 1,635 babies born to these women who did not experience an adverse event or death. The full table of results disaggregated by time-point is presented in supplementary material.

Outcomes

Figure 1 presents the outcomes for the 1,875 women admitted to a facility during the five observation periods. Nine women were admitted with postpartum haemorrhage and 1,866 were admitted for childbirth. Fourteen women admitted for childbirth were referred to another facility during labour. The main reason for referral was prolonged labour (Panel 1). Of the 1,852 women who gave birth in the facility, all delivered vaginally with one woman recorded as requiring an assisted delivery; caesarean sections are not available in primary healthcare facilities in this setting. Over half of births occurred on a weekday (56% [1,031/1,852]) between 7am and 7 pm (58% [1,067/1,852]). Two per cent (34/1,852) of deliveries were multiple births.

Postpartum, 45 women experienced an adverse event and three women died: the mortality rate was 1.6 per 1,000 women. Four women were referred intrapartum due to complications associated with multiple births and seven women were referred postpartum (Panel 1).

For the babies born to women who had an uncomplicated labour, 96 received resuscitation care of whom 19% subsequently died. Overall, 79 babies died: the perinatal mortality rate was 44.1 per 1,000 newborns (Figure 1). For babies born to women who experienced an adverse an event or died, 19% (9/48) were referred and four died: the perinatal morality rate among this group was 83.3 per 1,000 newborns.

Panel 1 Observers free text comments on reason for referral

In total 25 women were referred to another facility. Fourteen during labour - of whom one had eclampsia and three had severe pre-eclampsia; four women with multiple births were referred intrapartum; and seven were referred postpartum - of whom two had eclampsia and three experienced a postpartum haemorrhage event.

For peer review only - http://bmjopen.bmj.com/site/about/guidelines.xhtml

The most common reason for referral during labour was prolonged labour. One observer noted "*the mother was referred to a specialist hospital, because no progress of labour, [...] [the mother was] 4cm [dilated] after about 18 hours*" and another commented *"she was referred due to non-progress of labour despite augmentation with oxytocin*". One woman was referred because her baby was in a breech position.

For the four women diagnosed with severe pre-eclampsia/eclampsia during labour, insufficient assessment and monitoring of the woman during labour was noted: "*there were delays in needed treatment. No observations undertaken during the first hour of admission to detect disease condition and take appropriate action",* whilst another observed *"woman was admitted for normal labour but only vaginal examination was observed without physical nor vital signs or urine test".*

Five women were referred due to complications with the birth of the second twin: "*mother delivered first twin alive, mother referred […] as second twin was in transverse lie with hand prolapse"* and *"mother came with a history of twins. Delivered one at 9:48pm but the other baby refused to come out and the mother was referred in the morning around 8:39am*".

Two women with eclampsia were referred postpartum because their condition could not be stabilised. Both women experienced convulsions and lost consciousness. They both received magnesium sulphate before referral: one woman's baby was born alive and referred with the mother; the other was a fresh stillbirth.

For the three women who experienced a postpartum haemorrhage event none had severe postpartum haemorrhage (abnormal bleeding of more than 1000 mL). All received oxytocin in the facility before referral: one woman's condition was reported not to be stable and the baby required resuscitation before referral. For the other two women, who were reported to be stable, their baby was referred with them.

The remaining woman referred postpartum was referred because "*mother had a retain placenta, mentor and birth attendant tried everything but it failed*". The newborn was referred with their mother.

Provision of evidence-based care

All 1,804 women who had an uncomplicated labour were observed during the second and third stage of labour. Observers were required to prioritise the second and third stage of labour so the number of women and newborns observed during other stages of childbirth varied (Table 1).

Table 1 Proportion of all women admitted for childbirth who were observed, disaggregated by stage of care

Stage of care	Percentage observed	
	Women	Newborns
Initial assessment and history taking	99.8% (1,801/1,804)	n/a
First stage of labour	59.3% (1,069/1,804)	
Second & third stage of labour	100% (1,804/1,804)	
Immediate newborn & postpartum care	94.1% (1,697/1,804)	98.9% (1,617/1,635)

For peer review only - http://bmjopen.bmj.com/site/about/guidelines.xhtml

History taking and Initial assessment

In total, 1,801 women were observed during the initial assessment and history taking. Birth attendants were routinely observed to check the women's record for or, if not available, asked women's age, length of pregnancy and parity; 80% (95%CI 73-86), see

Figure 2. However, other aspects of history taking including asking about complications during both current and, if relevant, previous pregnancies were very low: 12% (95%CI 9-16) and 18% (95%CI 15-23), respectively. The most common complications that birth attendants asked about for the current pregnancy were fever (37% [81/218]), vaginal bleeding (32% [70/218]) and severe abdominal pain (25% [54/218]).

The majority of women were encouraged to have a support person present during labour and birth (63% [95%CI 53–72]). While around two thirds of birth attendants explained procedures to woman (and their support person) before proceeding (66% [95%CI 57–74]), less than a third of women (and their support person) were asked if they had any questions (32% [95%CI 25–40]).

Vaginal examination was almost universal (98% [95%CI 97-99]) but the proportion of women who had their temperature and blood pressure measured, and the foetal heart rate checked was low: 4% (95%CI 2-7), 30% (95%CI 23-39) and 22% (95%CI 16–29), respectively (

Figure 2). Few birth attendants washed their hands with soap and water or antiseptic before examining women (28% [95%CI 21-37]).

.

For peer review only

For peer review only - http://bmjopen.bmj.com/site/about/guidelines.xhtml

First stage of labour

In total, 1,069 women were observed during first stage of labour. Partograph was used to monitor labour in just 24% (95%CI 18-32) of cases (

Figure 3). Data were not collected on whether there was a delay in the progress of labour but 17% (95%CI 12-24) of women had their labour augmented with oxytocin and 6% (95%CI 4-9) of women's membranes were artificially ruptured.

The median number of vaginal examinations undertaken during the first stage of labour was one (IQR 1-2). Birth attendants were observed to wash their hands with soap & water or use antiseptic 39% (95%CI 30-49) of the time prior to any examination of woman, although a greater proportion were observed to wear high-level disinfectant or surgical gloves when performing the vaginal exam 76% (95%CI 64-85).

Seventy two per cent (95%CI 63–79) of women had a support person present at some point during labour (

Figure 3). Just under half of birth attendants were observed to explain to the woman (and their support person) what will happen during labour (49% [95%CI 42–56]). The majority of birth attendants were observed to encourage the woman to consume fluids/food at least once during labour (89% [95%CI 85-92]) and to encourage or help woman to ambulate and assume different positions (73% [95%CI 66–80]). During more than half of births observed, birth attendants were observed to drape women with a cloth, one under the buttocks and one over the abdomen (59% [95%CI 48–69]).

Data were collected on whether clean cloths/blankets, tie or cord clamp, sterile blade to cut cord, suction device and bag and mask (either size 0 or 1) were laid out in preparation for birth. Cord clamps, sterile blade and at least two cloths/blankets were available for over 90% of women (Figure 3). However, preparation of a bag and mask was substantially lower at 40% (95%CI 31-50).

Second and third stage of labour

All 1,804 women were observed during the second and third stage of labour. Just over half of women observed were assisted at some point during the second and third stage of labour by more than one healthcare worker (57% ([95%CI 50-63]) and 36% (95%CI 27-45) had a support person present (Figure 4). Women universally gave birth in the lithotomy position (97% [95% CI 93-98]). The use of episiotomy was very low (1% [95%CI 1-2]). Other inappropriate practices were also low: 3% (95%CI 1-7) fundal pressure, 1% (95%CI 0-2) excessive stretching of perineum and less than 1% had lavage of uterus.

The use of prophylactic uterotonic drugs immediately after birth was universal (96% [95%CI 93-98]), and 65% (95%CI 56-73) of birth attendants checked for the presence of a second baby before administering a uterotonic. Of women that received a uterotonic, 58% received oxytocin (999/1,735) and 42% misoprostol (733/1,735). Fourteen per cent (95%CI 9-20) of deliveries where uterotonics were administered were given within 1 minute of birth and for 52% (95%CI 44-57) of women a uterotonic was administered more than 3 minutes after birth. Immediately following the delivery/expulsion of the placenta, 75% (95%CI 69-80) of birth attendants were observed to perform uterine massage.

For peer review only - http://bmjopen.bmj.com/site/about/guidelines.xhtml

257 The use of controlled cord traction was consistently undertaken by all cadres of birth
258 attendants: skilled 91% (95%CI 83-96), CHEW 85% (95%CI 76-92) and 'other' birth attendant
259 84% (95%CI 77-89).

260 Following the birth of the placenta, 49% (95%CI 39-58) of birth attendants were observed to
261 assess the completeness of placenta and membranes, a considerably higher proportion (87%
262 [95%CI 79-92]) assessed for perineal and vaginal lacerations.

263 *Immediate newborn and postpartum care*

264 During the immediate postpartum period, 1,617 newborns and 1,697 women were observed.
265 The majority of newborns received all three elements of thermal care (immediate drying, skin-
266 to-skin and not bathing in the first hour) and clean cord care (ties or clamps cord when
267 pulsation stop or within 2-3 minutes after birth and cuts cord with clean scissors or blade):
268 71% (95%CI 60-80) and 92% (95%CI 89-95), respectively (

269 Figure 5). Eighty-six per cent (95%CI 75-93) of newborns had chlorohexidine applied to their
270 cord within the first hour of birth. However, breastfeeding was initiated within the first hour after
271 birth in just under half of newborns: 49% (95%CI 39-59).

272 Eighty-nine per cent (95%CI 79-95) of newborns were weighed. Of these infants, 95%
273 (1,122/1,182) weighed more than 2.5kg (Figure 5). Mothers and newborns were universally
274 kept in the same room after birth (rooming in), but only 61% (95%CI 51-71) were kept skin-to-
275 skin during the first hour.

276 For the fifteen babies born to mothers known to have HIV, 33% (95%CI 14-60) were observed
277 to receive antiretroviral therapy. The proportion of newborns on postnatal care wards who
278 received a vitamin K injection, tetracycline eye ointment and had their temperature checked
279 within 15 minutes after birth was close to zero throughout the study period (Figure 5): 0.1%
280 (95%CI 0-0.4), 4% (95%CI 2-7) and 2% (95%CI 1-5), respectively.

281 Just 3% (95%CI 2-6) of women had their vital signs checked 15 minutes after birth and 2%
282 (95%CI 1-5) of women received antibiotics.

283

284 Discussion

285 Our observations in ten primary healthcare facilities in northeast Nigeria indicated that, while
286 some essential processes of childbirth care were performed for almost all women and
287 newborns, the proportion of women who consistently received evidence-based care during
288 uncomplicated labour was low. Three percent of women (48/1,852) who gave birth at the
289 facility experienced major risks and complications while at the facility, including six severe pre-
290 eclampsia/eclampsia and six twin deliveries. Three women died. There were large numbers
291 of perinatal deaths and newborn referrals among this group of women. Further, among women
292 who we defined as having an uncomplicated labour 79 perinatal deaths were recorded, of
293 which almost three-quarters were intrapartum stillbirths.

294 Our study highlights substantial variation in the implementation of recommended evidence-
295 based interventions both within and across the different stages of childbirth: during all four
296 stages, fewer than half of measures reached more than 80% of women. Implementation was
297 highest for clinical interventions at the time of birth that have received international attention,
298 for example, the provision of prophylactic uterotonic and newborn thermal and clean cord care.
299 Implementation was lowest for measures designed as risk assessments, for example, history-

For peer review only - http://bmjopen.bmj.com/site/about/guidelines.xhtml

taking or checking vital signs. All measures related to risk assessment during the initial assessment (asks about complications in current and previous pregnancies, takes temperature and blood pressure, checks foetal heart rate) were completed for fewer than 30% of women observed.

The evidence-based care measures included in this study were based on WHO guidelines for essential intrapartum care [15]: their implementation should be covered in basic pre-service training for midwives, nurses and CHEWs in this setting; and they require only basic supplies of equipment. However, half of all observed births were attended by 'other' staff who would be defined as unskilled birth attendants both globally and in the Nigerian context: not doctors, nurses or midwives, nor CHEWs who have received additional training under Nigeria's task-shifting policy. Even so, far fewer than half of the women received many measures, raising questions even about the behaviours of birth attendants who have received training [43]. Further, although some variation was observed over time, in general, coverage of individual measures remained relatively constant throughout the observation period, including for a number of practices that are no longer recommended by WHO, for example, uterine massage [44]. Taken together, this study suggests that there may be limited opportunities for birth attendants to keep their knowledge up-to-date and enhance their skills once in post. Implementation research is needed to identify mechanisms to continuously support and improve healthcare worker practices, such as the use of checklists and in-service supervision and coaching, which has shown potential in other settings to improve uptake of and adherence to essential birth practices [39 45].

A potential alternative reason for the variation in care across the continuum is that some birth attendants were working alone for extended periods. In these circumstances, birth attendants may prioritise interventions during the third stage of labour and immediate newborn care. Higher workload, as a result of staff shortages, has been found to limit healthcare workers time for history-taking, thorough assessment of women, and ability to provide timely care [37 46]. Further, when working alone, birth attendants struggle to implement multiple recommended interventions simultaneously, for example, administering uterotonic and providing essential newborn care. While administering uterotonic was universal, less than half of women received a uterotonic within three minutes of birth. The importance of the exact timing of the application of uterotonic is not well-established, and as such, it is not clear whether this should be prioritised over immediate newborn care interventions [47 48] and clearer guidelines may be needed if it is important to prioritise certain components of care that cannot be done simultaneously by a single healthcare worker [49].

Poor assessment of women and newborns before and after birth has previously been documented in India and other African settings [50-52]. Observations of WHO-recommended practices for screening of pre-eclampsia/eclampsia in six sub-Saharan African countries, similarly found that a low proportion of women admitted to labour and delivery services were asked about danger signs, but substantially more women (77%) had their blood pressure checked on admission [51]. Low implementation of evidence-based care measures during the initial assessment and postpartum period indicate potential missed opportunities to identify and manage complications, as evident in the referral cases (Panel 1). Referrals post- and intrapartum included women with high-risk pregnancies such as severe pre-eclampsia/eclampsia, breech position and twins who should have been referred to a higher level of care [53]. This study has identified proper risk assessment at the time of birth as a priority, not least because, in this setting, only 37% of women attended at least four antenatal

For peer review only - http://bmjopen.bmj.com/site/about/guidelines.xhtml

care visits and 10% of women and 7% of newborns received a postnatal check within 2-days of birth in 2015 [54].

Poor quality of care has been acknowledged as a critical roadblock in Nigeria's attempt to reach universal health coverage. The Federal government has committed to strengthening Nigeria's health system, particularly primary healthcare, and specifically to accelerate the reduction of maternal and neonatal mortality by expanding access to, and quality of, maternal and child health services [55]. The implementation of the national strategy is supported by Nigeria's participation in the Network for Improving Quality of Care for Maternal, Newborn and Child Health (Quality of Care Network) [56]. The processes of care prioritised in these strategies focus on clinical interventions at the time of birth, including use of uterotonic drugs, skin-to-skin and chlorhexidine for umbilical cord care – interventions that were found to be routinely well implemented in this low resource primary healthcare setting. This study's findings suggest if these policies are to have an impact they need to extend their focus to also include basic risk-assessment. Further, while they aim to support healthcare workers at the health facilities through quality improvement cycles, clinical mentoring and peer-to-peer learning they may also need to consider the quality of pre-service training.

Strengths and Limitations

This study provides unique insight into the provision of evidence-based practices during childbirth and highlights clear areas for action in this setting, actions that are also likely to be relevant elsewhere. A particular strength was the relatively large sample size from multiple time points although, due to the study protocol to prioritise events closest to birth, over 40% of women were not observed during the first stage of labour. The missing observations are not anticipated to impact on findings as non-observation was random, with women who were not observed during the first stage of labour unlikely to differ systematically from those who were. Being observed may have impacted on birth attendants' behaviour; evidence suggests that being observed can positively improve behaviours although any change is likely to be short lived [57 58]. The sample size was estimated to be sufficiently large to reduce the impact of any potential Hawthorne effect and the relative consistency overtime and the low levels of implementation of many measures suggest that any effect of being observed was minimal.

This study was completed in the 10 primary healthcare facilities with the highest volume of births in Gombe State and is not therefore representative of all primary healthcare facilities. It is anticipated that these facilities represent the 'best' care available at the primary level and therefore findings are likely to overestimate the provision of evidence-based care available to the wider population. These findings support the growing body of evidence that giving birth in a primary healthcare facility might not be sufficient to ensure the effective care of women and newborns [1 4 59], and raise questions about the safety and quality of rural primary healthcare facilities that have low-volume of deliveries [16 60].

Conclusions

The recommendation for women to deliver in health facilities is designed to improve birth outcomes. This study of clinical observations of labour and the immediate postpartum period in primary facilities in Gombe State has revealed that, while some processes of clinical care were well adhered to, most women delivering in primary healthcare facilities do not receive the complete repertoire of childbirth care that they and their newborns needed to benefit from their choice to deliver in a health facility. In particular, few women or newborns benefited from even basic risk assessments, leading to missed opportunities to identify risks and

For peer review only - http://bmjopen.bmj.com/site/about/guidelines.xhtml

consequently late referrals and deaths. To continue with the recommendation of childbirth care in primary healthcare in high mortality settings like Gombe it is crucial that birth attendant capacity, capability and prioritisation processes are purposively addressed.

For peer review only

For peer review only - http://bmjopen.bmj.com/site/about/guidelines.xhtml

Supporting information

Acknowledgements
The authors would like to thank Data Research and Mapping Consult for coordinating the data collection, the Gombe State Primary Health Care Development Agency, Gombe State Ministry of Health and our partners Society for Family Health and Pact Nigeria for their support in carrying out this study. We would like to thank all the participants who contributed to our study.

Ethical approval and consent to participate
This research was conducted with approval from the Federal Ministry of Health Abuja, Nigeria, the State Ministry of Health Gombe State, Nigeria and the London School of Hygiene & Tropical Medicine (reference 12181).

Funding
This work is part of the IDEAS (Informed Decisions for Actions to improve maternal and newborn health) project. IDEAS is funded through a grant from the Bill & Melinda Gates Foundation to the London School of Hygiene & Tropical Medicine. Gates Global Health Grant Number: OPP1149259/INV-007644. The funder had no role in study design, data collection and analysis, decision to publish or preparation of the manuscript.

Competing interests
We have read the BMJ policy and the authors declare that they have no competing interests.

Authors' contributions
JE conducted the analysis and drafted the manuscript. CH supported the refinement of our definition of evidence-based care and reviewed the manuscript for intellectual content. NU, BW and AS all critically reviewed the manuscript for intellectual content. TM conceived the study and drafted the manuscript. All authors have read and approved the final version of the manuscript.

Data sharing
The data are available from the principal investigator of the IDEAS project and co-author for this manuscript, Dr Tanya Marchant ORCID id 0000-0002-4228-4334. Reuse permitted on request.

Patient consent and confidentiality
Participation in the study was voluntary and participants were free to withdraw from the study at any time without giving a reason. Researchers explained the purpose of the study and answered any questions participants had. The free and informed consent of all women and birth attendants observed was obtained before participating.

Patient and public involvement
Patients or the public were not involved in the design, conduct, reporting or dissemination plans of our research. Observations were recorded in English and pre-testing completed in health facilities by staff.

For peer review only

For peer review only - http://bmjopen.bmj.com/site/about/guidelines.xhtml

References

1. Campbell OM, Calvert C, Testa A, et al. The scale, scope, coverage, and capability of childbirth care. *Lancet (London, England)* 2016;388(10056):2193-208. doi: 10.1016/s0140-6736(16)31528-8 [published Online First: 2016/09/20]
2. Gabrysch S, Nesbitt RC, Schoeps A, et al. Does facility birth reduce maternal and perinatal mortality in Brong Ahafo, Ghana? A secondary analysis using data on 119 244 pregnancies from two cluster-randomised controlled trials. *The Lancet Global health* 2019;7(8):e1074-e87. doi: 10.1016/s2214-109x(19)30165-2 [published Online First: 2019/07/16]
3. Randive B, Diwan V, De Costa A. India's Conditional Cash Transfer Programme (the JSY) to Promote Institutional Birth: Is There an Association between Institutional Birth Proportion and Maternal Mortality? *PloS one* 2013;8(6):e67452. doi: 10.1371/journal.pone.0067452 [published Online First: 2013/07/05]
4. Scott S, Ronsmans C. The relationship between birth with a health professional and maternal mortality in observational studies: a review of the literature. *Tropical medicine & international health : TM & IH* 2009;14(12):1523-33. doi: 10.1111/j.1365-3156.2009.02402.x [published Online First: 2009/10/02]
5. Kruk ME, Gage AD, Joseph NT, et al. Mortality due to low-quality health systems in the universal health coverage era: a systematic analysis of amenable deaths in 137 countries. *Lancet (London, England)* 2018;392(10160):2203-12. doi: 10.1016/s0140-6736(18)31668-4 [published Online First: 2018/09/10]
6. Kruk ME, Gage AD, Arsenault C, et al. High-quality health systems in the Sustainable Development Goals era: time for a revolution. *The Lancet Global Health* 2018;6(11):e1196-e252. doi: 10.1016/S2214-109X(18)30386-3
7. World Health Organization. Strategies towards ending preventable maternal mortality (EPMM). Geneva: World Health Organization 2015:44 p. Executive summary published as WHO/RHR/15.03.
8. Merali HS, Lipsitz S, Hevelone N, et al. Audit-identified avoidable factors in maternal and perinatal deaths in low resource settings: a systematic review. *BMC pregnancy and childbirth* 2014;14:280. doi: 10.1186/1471-2393-14-280 [published Online First: 2014/08/19]
9. Hobbs AJ, Moller AB, Kachikis A, et al. Scoping review to identify and map the health personnel considered skilled birth attendants in low-and-middle income countries from 2000-2015. *PloS one* 2019;14(2):e0211576. doi: 10.1371/journal.pone.0211576 [published Online First: 2019/02/02]
10. Radovich E, Benova L, Penn-Kekana L, et al. 'Who assisted with the delivery of (NAME)?' Issues in estimating skilled birth attendant coverage through population-based surveys and implications for improving global tracking. *BMJ global health* 2019;4(2):e001367. doi: 10.1136/bmjgh-2018-001367 [published Online First: 2019/05/30]
11. Amouzou A, Leslie HH, Ram M, et al. Advances in the measurement of coverage for RMNCH and nutrition: from contact to effective coverage. *BMJ global health* 2019;4(Suppl 4):e001297. doi: 10.1136/bmjgh-2018-001297 [published Online First: 2019/07/13]
12. Tuncalp, Were WM, MacLennan C, et al. Quality of care for pregnant women and newborns-the WHO vision. *BJOG : an international journal of obstetrics and gynaecology* 2015;122(8):1045-9. doi: 10.1111/1471-0528.13451 [published Online First: 2015/05/02]
13. Miller S, Abalos E, Chamillard M, et al. Beyond too little, too late and too much, too soon: a pathway towards evidence-based, respectful maternity care worldwide. *Lancet (London, England)* 2016;388(10056):2176-92. doi: 10.1016/s0140-6736(16)31472-6 [published Online First: 2016/09/20]
14. Oladapo O, Tunçalp Ö, Bonet M, et al. WHO model of intrapartum care for a positive childbirth experience: transforming care of women and babies for improved health and

For peer review only - http://bmjopen.bmj.com/site/about/guidelines.xhtml

wellbeing. *BJOG: An International Journal of Obstetrics & Gynaecology* 2018;125(8):918-22. doi: 10.1111/1471-0528.15237

15. World Health Organization. WHO recommendations: intrapartum care for a positive childbirth experience. Geneva: World Health Organization, 2018.
16. Kruk ME, Leslie HH, Verguet S, et al. Quality of basic maternal care functions in health facilities of five African countries: an analysis of national health system surveys. *The Lancet Global health* 2016;4(11):e845-e55. doi: 10.1016/s2214-109x(16)30180-2 [published Online First: 2016/10/22]
17. The DHS Program. Service Provision Assessment (SPA) Overview.online: https://dhsprogram.com/What-We-Do/Survey-Types/SPA.cfm [accessed on 16 January 2020]
18. Moxon SG, Ruysen H, Kerber KJ, et al. Count every newborn; a measurement improvement roadmap for coverage data. *BMC pregnancy and childbirth* 2015;15 Suppl 2:S8. doi: 10.1186/1471-2393-15-s2-s8 [published Online First: 2015/09/24]
19. Bhattacharya AA, Allen E, Umar N, et al. Monitoring childbirth care in primary health facilities: a validity study in Gombe State, northeastern Nigeria. *Journal of global health* 2019;9(2):020411. doi: 10.7189/jogh.09.020411 [published Online First: 2019/07/31]
20. Gombe State Government. History of Gombe State, Nigeria. 2018;online: http://gombestate.gov.ng/history-2/ [accessed on 9 September 2019]
21. Oruonye E, Abubakar H, Ahmed M, et al. Hiv/Aids interventions in Gombe state Nigeria; challenges of sustaining the gains. *International Journal of Asian Social Science* 2017;7(6):448-57.
22. Izugbara CO, Wekesah FM, Adedini SA. Maternal Health in Nigeria. A Situation Update. Nairobi, Kenya: African Population and Health Research Centre, 2016.
23. National Bureau of Statistics and United Nations Children's Fund. Multiple Indicator Cluster Survey 2016–17, Survey Findings Report. Abuja: Nigeria National Bureau of Statistics and United Nations Children's Fund, 2017.
24. National Population Commission and ICF. Nigeria Demographic and Health Survey 2018. Key Indicators Report. Abuja, Nigeria, and Rockville, Maryland, USA, 2019.
25. Nigeria Health Management Information System. Gombe State facility deliveries, by facility type, January-December 2018. *Nigeria District Health Information System, version 2* 2019
26. Umar N, Quaife M, Exley J, et al. Toward improving respectful maternity care: a discrete choice experiment with rural women in northeast Nigeria. *BMJ global health* 2020;5(3):e002135. doi: 10.1136/bmjgh-2019-002135 [published Online First: 2020/03/24]
27. Bhattacharya AA, Umar N, Audu A, et al. Quality of routine facility data for monitoring priority maternal and newborn indicators in DHIS2: A case study from Gombe State, Nigeria. *PloS one* 2019;14(1):e0211265. doi: 10.1371/journal.pone.0211265 [published Online First: 2019/01/27]
28. Gombe State Ministry of Health. Gombe state framework for the implimentation of expanded access to family planning services. 2012;online: https://www.fhi360.org/sites/default/files/media/documents/nigeria-gombe-state-framework.pdf [accessed on 19 June 2020]
29. Gombe State Ministry of Health. Gombe State government strategic health development plan. 2010;online: https://drive.google.com/file/d/0B1DAmtM1BcbMeVBRUjNEakViXzQ/view [accessed on 19 June 2020]
30. Federal Ministry of Health. Task-shifting and task-sharing policy for essential health care services in Nigeria. Abuja, Nigeria, 2014.
31. Exley JL, Umar N, Moxon S, et al. Newborn resuscitation in Gombe State, northeastern Nigeria. *Journal of global health* 2018;8(2):020420. doi: 10.7189/jogh.08.020420 [published Online First: 2018/11/10]
32. Marchant T. Change in maternal and newborn health care. Interactions between families and frontline workers - their frequency, quality and equity - and coverage of

For peer review only - http://bmjopen.bmj.com/site/about/guidelines.xhtml

interventions of mothers and newborns. Report from Gombe State, Nigeria, 2012-2015. online: https://ideas.lshtm.ac.uk/wp-content/uploads/2017/08/Marchant_IDEAS_Gombe_State_Follow-upSurvey_2016.pdf [accessed on 9 September 2019]: IDEAS, London School of Hygiene & Tropical Medicine, 2016.
33. E4A-MamaYe. Gome PHC stockout of essential life-saving commodities scorecards. Jan - June 2018. 2018;online: https://mamaye.org/resources/scorecards/gombe-phc-stockout-essential-life-saving-commodities-scorecard [accessed on 20 June 2020]
34. IDEAS team. Informed Decisions for Actions in Maternal and Newborn Health 2010-17 Report: What works, why and how in maternal and newborn health. online: https://ideas.lshtm.ac.uk/wp-content/uploads/2017/09/LSHTM_IDEAS_PhaseIReport_Web-reduced.pdf [accessed on 9 August 2019]: IDEAS, London School of Hygiene & Tropical Medicine, 2017.
35. Makowiecka K, Marchant T, Betemariam W, et al. Characterising innovations in maternal and newborn health based on a common theory of change: lessons from developing and applying a characterisation framework in Nigeria, Ethiopia and India. *BMJ global health* 2019;4(4):e001405. doi: 10.1136/bmjgh-2019-001405 [published Online First: 2019/08/14]
36. Makowiecka K. The Pathway to Improved Maternal and Newborn Health Outcomes. Use of data for maternal and newborn health in Gombe State, Nigeria. online: https://ideas.lshtm.ac.uk/wp-content/uploads/2017/08/Makowiecka_IDEAS_Pathway_Improved_Maternal_Newborn_Health_Gombe_Nigeria_2016.pdf [accessed on 9 September 2019]: IDEAS, London School of Hygiene & Tropical Medicine, 2016.
37. Shobo OG, Umar N, Gana A, et al. Factors influencing the early initiation of breast feeding in public primary healthcare facilities in Northeast Nigeria: a mixed-method study. *BMJ open* 2020;10(4):e032835. doi: 10.1136/bmjopen-2019-032835
38. USAID. Maternal and Newborn Quality of Care Surveys. *Maternal and Child Health Integrated Program* 2013;online: https://www.mchip.net/qocsurveys/ [accessed on 10 September 2019]
39. Marx Delaney M, Maji P, Kalita T, et al. Improving Adherence to Essential Birth Practices Using the WHO Safe Childbirth Checklist With Peer Coaching: Experience From 60 Public Health Facilities in Uttar Pradesh, India. *Global Health: Science and Practice* 2017;5(2):217-31. doi: 10.9745/GHSP-D-16-00410
40. World Health Organization. Standards for improving quality of maternal and newborn care in health facilities. Geneva: World Health Organization, 2016.
41. World Health Organization. Consultation on improving measurement of the quality of maternal, newborn and child care in health facilities. Geneva: World Health Organization, 2013.
42. World Health Organization, United Nations Population Fund, UNICEF. Pregnancy, childbirth, postpartum and newborn care. A guide for essential practice (3rd edition). Geneva: World Health Organization, 2015.
43. Harvey SA, Blandon YC, McCaw-Binns A, et al. Are skilled birth attendants really skilled? A measurement method, some disturbing results and a potential way forward. *Bulletin of the World Health Organization* 2007;85(10):783-90. doi: 10.2471/blt.06.038455 [published Online First: 2007/11/27]
44. World Health Organization. WHO recommendations for the prevention and treatment of postpartum haemorrhage. Geneva: World Health Organization, 2012.
45. Semrau KEA, Hirschhorn LR, Marx Delaney M, et al. Outcomes of a Coaching-Based WHO Safe Childbirth Checklist Program in India. *The New England journal of medicine* 2017;377(24):2313-24. doi: 10.1056/NEJMoa1701075 [published Online First: 2017/12/14]
46. Munabi-Babigumira S, Glenton C, Lewin S, et al. Factors that influence the provision of intrapartum and postnatal care by skilled birth attendants in low- and middle-income

For peer review only - http://bmjopen.bmj.com/site/about/guidelines.xhtml

countries: a qualitative evidence synthesis. *Cochrane Database of Systematic Reviews* 2017(11) doi: 10.1002/14651858.CD011558.pub2
47. Vogel JP, Williams M, Gallos I, et al. WHO recommendations on uterotonics for postpartum haemorrhage prevention: what works, and which one? *BMJ global health* 2019;4(2):e001466. doi: 10.1136/bmjgh-2019-001466
48. Bartlett L, Cantor D, Lynam P, et al. Facility-based active management of the third stage of labour: assessment of quality in six countries in sub-Saharan Africa. *Bulletin of the World Health Organization* 2015;93(11):759-67. doi: 10.2471/blt.14.142604 [published Online First: 2015/11/10]
49. Moller AB, Newby H, Hanson C, et al. Measures matter: A scoping review of maternal and newborn indicators. *PloS one* 2018;13(10):e0204763. doi: 10.1371/journal.pone.0204763 [published Online First: 2018/10/10]
50. de Graft-Johnson J, Vesel L, Rosen HE, et al. Cross-sectional observational assessment of quality of newborn care immediately after birth in health facilities across six sub-Saharan African countries. *BMJ open* 2017;7(3):e014680. doi: 10.1136/bmjopen-2016-014680 [published Online First: 2017/03/30]
51. Rawlins B, Plotkin M, Rakotovao JP, et al. Screening and management of pre-eclampsia and eclampsia in antenatal and labor and delivery services: findings from cross-sectional observation studies in six sub-Saharan African countries. *BMC pregnancy and childbirth* 2018;18(1):346. doi: 10.1186/s12884-018-1972-1
52. Saxena M, Srivastava A, Dwivedi P, et al. Is quality of care during childbirth consistent from admission to discharge? A qualitative study of delivery care in Uttar Pradesh, India. *PloS one* 2018;13(9):e0204607. doi: 10.1371/journal.pone.0204607 [published Online First: 2018/09/28]
53. Hanson C, Munjanja S, Binagwaho A, et al. National policies and care provision in pregnancy and childbirth for twins in Eastern and Southern Africa: A mixed-methods multi-country study. *PLoS medicine* 2019;16(2):e1002749. doi: 10.1371/journal.pmed.1002749 [published Online First: 2019/02/20]
54. Marchant T, Beaumont E, Makowiecka K, et al. Coverage and equity of maternal and newborn health care in rural Nigeria, Ethiopia and India. *CMAJ : Canadian Medical Association journal = journal de l'Association medicale canadienne* 2019;191(43):E1179-e88. doi: 10.1503/cmaj.190219 [published Online First: 2019/10/30]
55. Federal Government of Nigeria. Second National Strategic Health Development Plan 2018-2022. 2019;online: http://www.health.gov.ng/doc/NSHDP%20II%20Final.pdf [accessed on 3 February 2020]
56. Network for Improving Quality of Care for Maternal NaCH. Quality of Care in Nigeria. 2019;online: http://www.qualityofcarenetwork.org/country-data/nigeria [accessed on 3 February 2020]
57. Leonard K, Masatu MC. Outpatient process quality evaluation and the Hawthorne Effect. *Social Science & Medicine* 2006;63(9):2330-40. doi: https://doi.org/10.1016/j.socscimed.2006.06.003
58. Leonard KL. Is patient satisfaction sensitive to changes in the quality of care? An exploitation of the Hawthorne effect. *Journal of Health Economics* 2008;27(2):444-59. doi: https://doi.org/10.1016/j.jhealeco.2007.07.004
59. Sharma G, Powell-Jackson T, Haldar K, et al. Quality of routine essential care during childbirth: clinical observations of uncomplicated births in Uttar Pradesh, India. *Bulletin of the World Health Organization* 2017;95(6):419-29. doi: 10.2471/blt.16.179291 [published Online First: 2017/06/13]
60. Hanson C, Schellenberg J. Redesigning maternal health services: is centralisation the answer in low-resource settings? *BMJ global health* 2019;4(3):e001488. doi: 10.1136/bmjgh-2019-001488 [published Online First: 2019/07/03]

For peer review only - http://bmjopen.bmj.com/site/about/guidelines.xhtml

Figure legends

Figure 1 Women and newborn's pathway from admission

Figure 2 History taking and initial examination

[FIGURE]

FIGURE NOTE: red line represents the average percent across all five time points. For problems experienced during previous pregnancies 10% of observers recorded that they did not know if the birth attendant asked the women. For HIV status 30% of observers recorded that they did not know if it had been checked

Figure 3 Examinations and procedures during first stage of labour

[FIGURE]

FIGURE NOTE: red line represents the average percent across all five time points

Figure 4 Care received during second and third stage of labour

[FIGURE]

FIGURE NOTE: red line represents the average percent across all five time points

Figure 5 Immediate newborn and postpartum care

[FIGURE]

FIGURE NOTE: red line represents the average across all five time points. Administers chlorhexidine and weighs baby was not included in the checklist in the first observation period (time point 1).

For peer review only

For peer review only - http://bmjopen.bmj.com/site/about/guidelines.xhtml

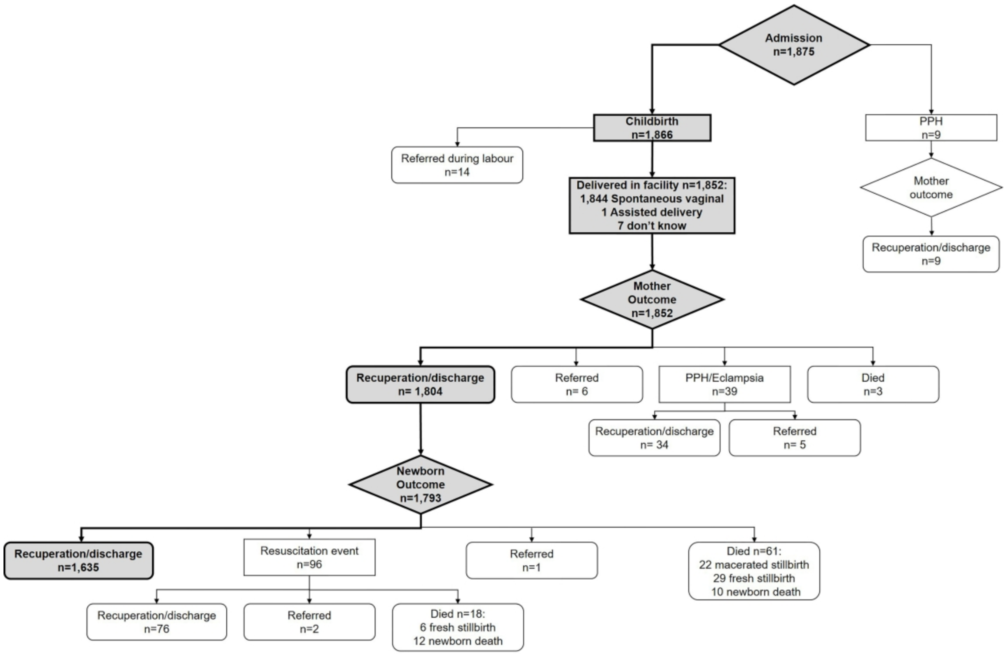

Figure 1 Women and newborn's pathway from admission

437x284mm (300 x 300 DPI)

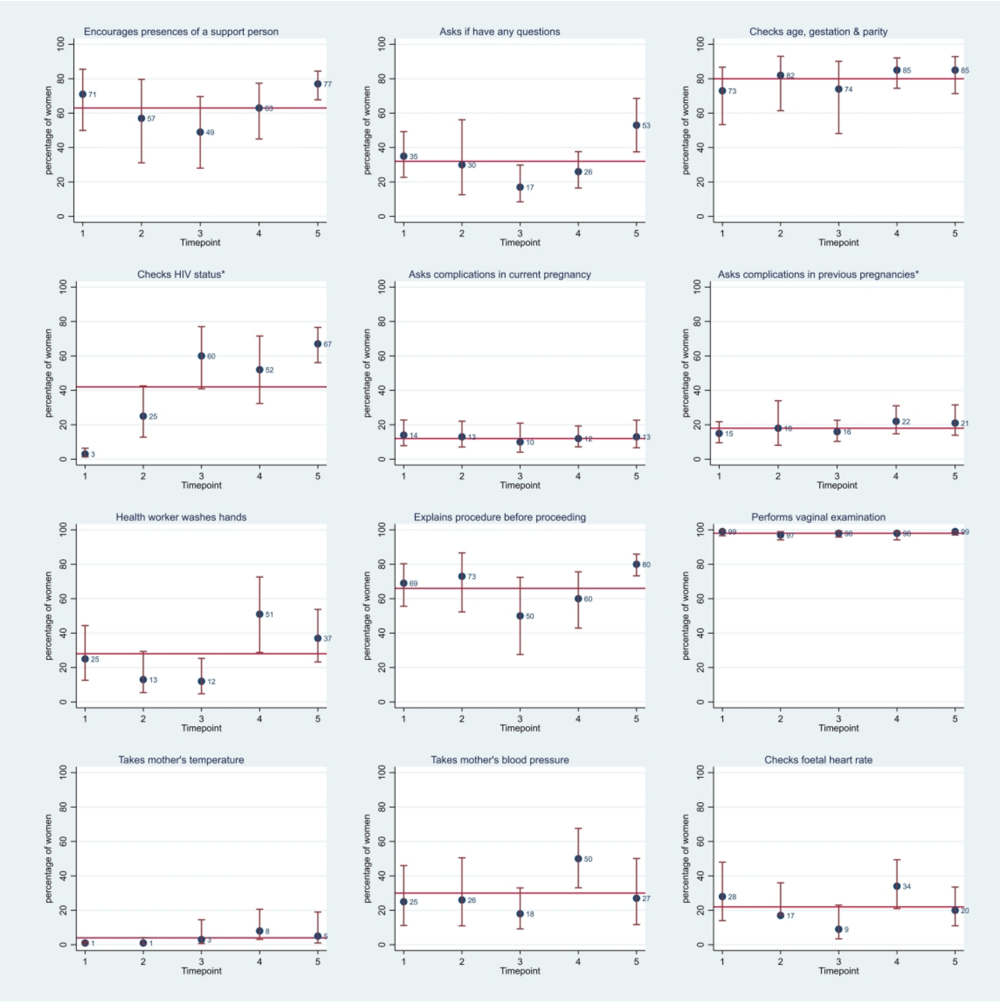

Figure 2 History taking and initial examination
[FIGURE]
FIGURE NOTE: red line represents the average percent across all five timepoints. For problems experienced during previous pregnancies 10% of observers recorded that they did not know if the birth attendant asked the women. For HIV status 30% of observers recorded that they did not know if it had been checked

360x360mm (300 x 300 DPI)

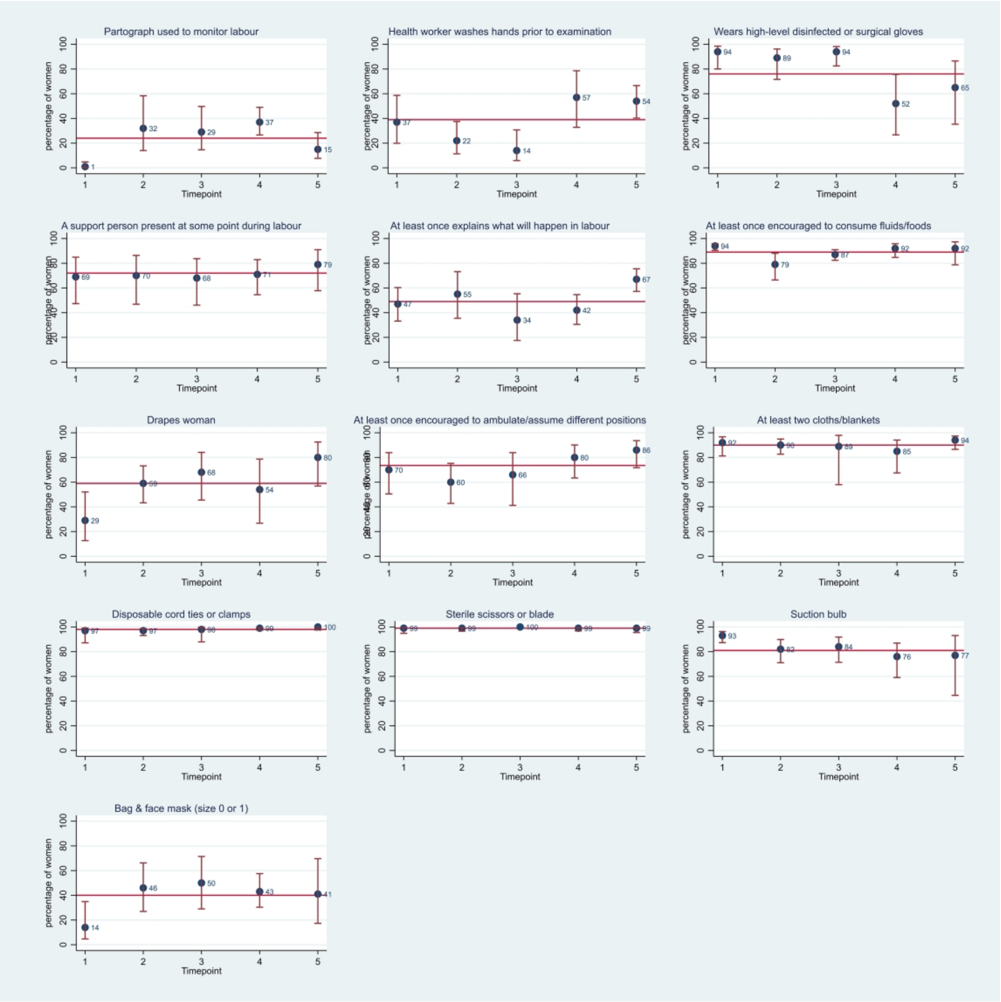

Figure 3 Examinations and procedures during first stage of labour
[FIGURE]
FIGURE NOTE: red line represents the average percent across all five timepoints

360x360mm (300 x 300 DPI)

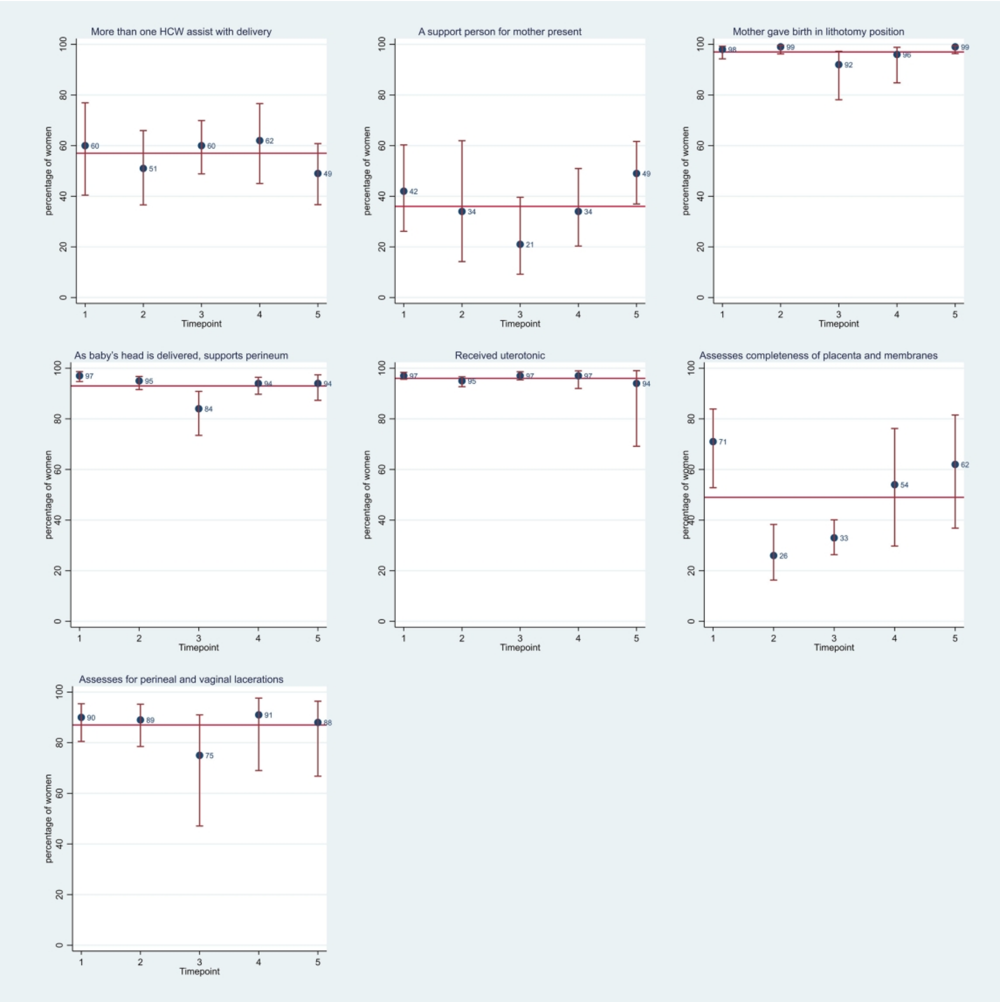

Figure 4 Care received during second and third stage of labour
[FIGURE]
FIGURE NOTE: red line represents the average percent across all five timepoints

240x240mm (300 x 300 DPI)

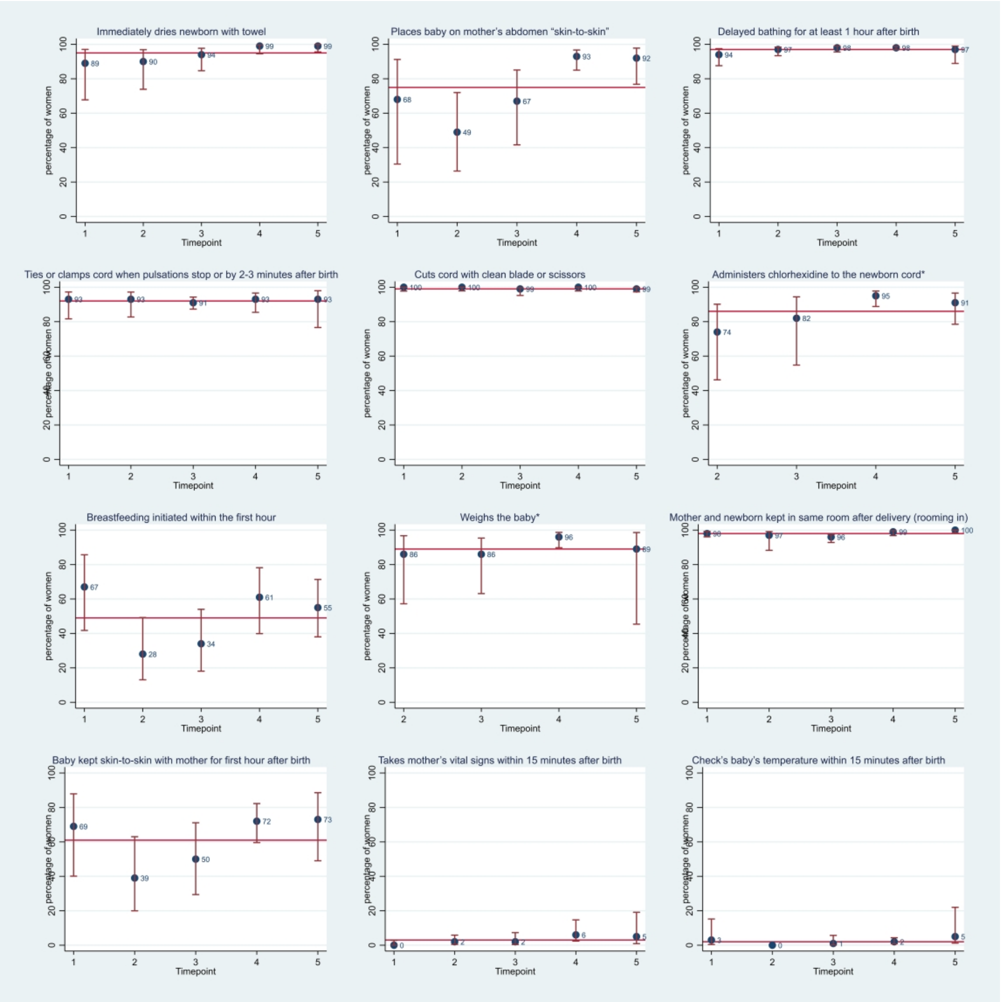

Figure 5 Immediate newborn and postpartum care

[FIGURE]

FIGURE NOTE: red line represents the average across all five timepoints. Administers chlorhexidine and weighs baby was not included in the checklist in the first observation period (timepoint 1).

Supplementary material: full table of results

	Observation period				
	1 (Aug 2016)	2 (Mar 2017)	3 (Aug 2017)	4 (Mar 2018)	5 (Aug 2018)
	Percentage of women (95%CI)				
**History taking & initial assessment**					
Encourages woman to have a support person	70.8% (50.0-85.5)	57.1% (31.1-79.7)	48.6% (28.0-69.6)	62.6% (45.0-77.4)	77.1% (67.8-84.4)
Asks if have any questions	34.8% (22.7-49.3)	30.0% (12.6-56.2)	16.6% (8.5-29.9)	25.7% (16.5-37.6)	53.4% (37.5-68.6)
Checks client card or asks client age, length of pregnancy and parity	73.2% (53.4-86.7)	82.3% (61.4-93.0)	74.4% (48.2-90.1)	85.3% (74.5-92.1)	85.0% (71.4-92.8)
Checks woman's HIV status	3.0% (1.4-6.3)	24.8% (12.8-42.6)	60.4% (40.9-77.0)	52.3% (32.4-71.6)	67.2% (56.1-76.6)
Asks whether experienced any complications during pregnancy	13.7% (7.9-22.8)	12.8% (7.1-22.1)	9.6% (4.0-20.9)	12.0% (7.2-19.3)	12.6% (6.6-22.7)
If had a previous pregnancy, whether experienced any complications during previous pregnancies	14.6% (9.5-21.8)	17.5% (8.1-34.0)	15.5% (10.3-22.6)	21.8% (14.7-31.0)	21.4% (13.9-31.6)
Health worker washes hands	25.3% (12.6-44.4)	13.4% (5.4-29.4)	11.5% (4.8-25.3)	50.9% (28.8-72.6)	37.2% (23.2-53.8)
Explains procedure to woman before proceeding	69.4% (55.6-80.3)	72.7% (52.3-86.6)	50.0% (27.6-72.4)	60.4% (42.9-75.6)	80.4% (73.3-85.9)
Performs vaginal examination	99.1% (96.6-99.8)	97.5% (94.2-998.9)	98.3% (95.8-99.3)	97.6% (94.2-99.0)	98.5% (97.0-99.3)
If performs vaginal exam, wears high-level disinfectant gloves	93.7% (82.8-97.9)	79.7% (48.6-94.2)	94.9% (84.3-98.5)	43.9% (21.3-69.3)	59.2% (35.9-79.0)
Takes temperature	0.6% (0.2-1.9)	1.1% (0.3-4.1)	3.4% (0.7-14.5)	8.3% (3.1-20.6)	4.7% (1.0-19.0)
Takes blood pressure	24.7% (11.2-46.0)	26.2% (11.0-50.5)	18.3% (9.2-33.0)	50.4% (33.1-67.6)	26.7% (11.7-50.0)
Checks foetal heart rate with fetoscope/Doppler/ultrasound	28.0% (14.0-48.0)	17.3% (7.2-36.0)	9.3% (3.4-23.0)	33.8% (21.0-49.4)	19.9% (11.0-33.5)
**First stage of labour: examination and procedures**					
Partograph used to monitor labour	0.6% (0.1-4.8)	32.3% (14.0-58.3)	29.1% (14.6-49.7)	37.1% (26.6-49.0)	15.4% (7.7-28.6)
Washes hands with soap & water or uses antiseptic prior to any examination of woman	37.2% (19.9-58.7)	21.7% (11.3-37.6)	14.3% (5.9-30.7)	57.2% (32.8-78.6)	53.7% (40.3-66.6)

For peer review only

Wears high-level disinfected or surgical gloves	94.2% (80.1-98.5)	88.9% (71.6-96.2)	94.2% (82.5-98.2)	51.6% (26.7-75.7)	65.2% (35.3-86.5)
Median number of vaginal examinations	2 (1-2)	1 (1-2)	2 (1-2)	1 (1-2)	1 (1-2)
Augments labour with oxytocin	14.5% (7.5-26.3)	12.6% (6.5-23.2)	24.9% (17.2-34.5)	18.0% (7.5-37.2)	15.4% (5.9-34.5)
Performs artificial rupture of membranes	7.6% (3.9-14.0)	9.6% (4.3-20.2)	9.0% (3.3-22.5)	3.5% (1.5-7.9)	1.8% (0.4-6.7)
A support person present at some point during labour	69.2% (47.3-84.9)	70.2% (46.8-86.3)	67.7% (46.1-83.7)	70.7% (54.5-82.9)	78.9% (57.8-91.0)
At least once explains what will happen in labour	46.5% (33.2-60.3)	55.1% (35.5-73.2)	33.9% (17.5-55.3)	42.1% (30.5-54.6)	67.0% (57.2-75.5)
At least encouraged to consume fluids/foods during labour	93.6% (90.4-95.8)	79.3% (66.5-88.1)	87.3% (82.4-91.0)	91.9% (84.7-95.8)	92.1% (78.7-97.3)
Drapes woman	28.5% (12.7-52.1)	59.1% (43.3-73.2)	67.7% (45.5-84.1)	53.7% (26.8-78.7)	80.2% (56.9-92.5)
At least once encourages woman to ambulate and assume different positions in labour	69.8% (50.5-83.9)	60.1% (42.8-75.2)	65.6% (41.2-83.9)	79.9% (63.4-90.1)	85.9% (71.7-93.6)
**First stage of labour: equipment and supplies laid out in preparation for delivery**					
At least two cloths/blankets	91.9% (81.2-96.7)	90.4% (82.7-94.9)	88.9% (58.0-97.9)	85.2% (67.5-94.1)	93.8% (86.6-97.3)
Disposable cord ties or clamps	96.5% (87.1-99.1)	97.0% (93.1-98.7)	98.4% (87.9-99.8)	99.3% (97.4-99.8)	99.6% (97.5-99.9)
Sterile scissors or blade	98.8% (94.9-99.8)	99.0% (96.6-99.7)	100%	98.9% (96.8-99.7)	98.7% (95.5-99.6)
Suction bulb	93.0% (87.3-96.3)	82.3% (71.0-89.9)	84.1% (71.4-91.8)	75.6% (59.1-87.0)	76.7% (44.6-93.0)
Bag & face mask (size 0 or 1)	14.0% (4.7-35.0)	46.0% (26.9-66.2)	50.3% (29.0-71.4)	43.5% (30.4-57.6)	41.0% (17.3-69.7)
**Second and third stage of labour**					
More than one HCW assist with delivery	60.1% (40.4-77.0)	51.4% (36.6-66.0)	59.8% (48.9-69.9)	62.1% (45.1-76.6)	48.7% (36.7-60.8)
A support person for mother present	42.3% (26.2-60.3)	34.2% (14.2-62.0)	20.5% (9.2-39.6)	34.0% (20.3-51.0)	49.3% (37.0-61.7)
Performs episiotomy	1.5% (0.4-5.4)	0.8% (0.2-4.4)	0.6% (0.1-2.4)	2.4% (1.0-5.8)	0.3% (0-2.6)
Mother gave birth in lithotomy position	97.9% (94.3-99.3)	98.9% (96.2-99.7)	91.9% (78.1-97.3)	95.6% (84.8-98.8)	98.5% (96.4-99.4)

For peer review only

As baby's head is delivered, supports perineum	97.3% (94.7-98.7)	94.7% (91.6-96.7)	84.0% (73.5-90.9)	93.9% (89.7-96.4)	94.1% (87.3-97.4)
Checks for another baby prior to giving the uterotonic	56.8% (31.7-78.9)	50.8% (33.6-67.9)	52.8% (37.9-67.2)	81.2% (59.5-92.7)	83.0% (67.9-91.9)
Received uterotonic	97.3% (95.6-98.4)	95.0% (92.7-96.6)	97.5% (95.4-98.6)	97.1% (92.0-99.0)	93.8% (69.2-99.0)
If uterotonic received, timing of administration					
At delivery	0.3% (0-2.4)	0.3% (0-2.7)	0.3% (0.1-1.3)	1.8% (0.6-5.0)	0
Within 1 minute of delivery	23.0% (9.4-46.4)	8.8% (2.7-25.0)	10.9% (5.8-19.5)	11.1% (4.9-23.1)	16.6% (9.0-28.6)
Within 3 minutes of delivery	28.5% (22.7-35.1)	30.7% (19.7-44.5)	26.1% (18.4-35.6)	37.0% (29.4-45.4)	51.3% (42.1-60.4)
More than 3 minutes	47.6% (31.6-64.1)	60.2% (41.1-76.7)	62.5% (54.0-70.3)	50.1% (37.9-62.4)	32.2% (25.3-39.9)
Applies traction to the cord while applying suprapubic counter traction	87.3% (80.8-91.8)	81.9% (61.9-92.7)	80.6% (70.5-87.9)	85.3% (67.3-94.3)	91.2% (71.7-97.7)
If skilled (doctor, nurse, midwife) birth assistant, applies traction to the cord while applying suprapubic counter traction	100%	95.4% (63.0-99.6)	88.7% (81.9-93.2)	82.4% (77.4-86.4)	90.5% (48.1-99.0)
If CHEW birth assistant, applies traction to the cord while applying suprapubic counter traction	87.6% (78.8-93.0)	80.9% (58.9-92.6)	81.9% (67.4-90.8)	85.7% (54.9-96.7)	88.9% (66.8-97.0)
If unskilled birth assistant, applies traction to the cord while applying suprapubic counter traction	86.1% (78.1-91.5)	77.8% (57.5-90.0)	77.0% (63.8-86.4)	85.2% (65.2-94.7)	93.0% (77.1-98.1)
Performs uterine massage immediately following the delivery of the placenta	73.7% (63.7-81.7)	78.6% (60.7-89.8)	63.8% (52.6-73.6)	79.0% (63.6-89.0)	77.4% (63.4-87.2)
Assesses completeness of placenta and membranes	70.7% (52.8-83.9)	25.8% (16.3-38.3)	32.9% (26.4-40.1)	53.8% (29.8-76.2)	61.6% (36.8-81.5)
Assesses for perineal and vaginal lacerations	90.2% (80.5-95.4)	89.4% (78.5-95.2)	75.0% (47.1-91.0)	90.5% (69.0-97.6)	88.0% (66.8-96.4)
**Immediate newborn and postpartum care**					
Immediately dries newborn with towel	89.3% (67.8-97.1)	90.5% (73.9-96.9)	93.9% (84.7-97.7)	98.7% (94.5-99.7)	99.4% (95.6-99.9)
Places baby on mother's abdomen "skin-to-skin"	68.1% (30.5-91.2)	49.0% (26.4-72.0)	66.9% (41.6-85.1)	92.8% (85.0-96.7)	92.3% (76.8-97.8)
Delayed bathing until at least 1 hour after birth	94.3% (87.6-97.5)	96.7% (93.4-98.4)	97.9% (95.6-99.0)	98.1% (96.9-99.0)	96.5% (88.9-99.0)

For peer review only

Ties or clamps cord when pulsations stop or by 2-3 minutes after birth	92.6% (81.6-97.3)	92.8% (82.7-97.2)	91.4% (87.3-94.3)	92.8% (85.4-96.6)	92.7% (76.6-98.0)
Cuts cord with clean blade or scissors	99.7% (97.8-100)	99.7% (97.9-100)	98.8% (95.2-99.7)	99.7% (97.8-100)	99.4% (97.3-99.9)
Administers chlorhexidine to the newborn cord	Not measured	73.7% (46.0-90.2)	81.9% (54.6-94.5)	95.0% (88.8-97.8)	91.1% (78.4-96.6)
Breastfeeding initiated within the first hour	67.5% (41.8-85.7)	27.6% (13.1-49.2)	33.7% (18.1-54.0)	60.6% (39.9-78.2)	55.3% (38.0-71.4)
Check's baby's temperature within 15 minutes after birth	2.7% (0.4-15.2)	0%	0.6% (0.1-5.7)	1.9% (0.8-4.3)	5.4% (1.2-22.0)
Takes mother's vital signs 15 minutes after birth	0.3% (0-2.8)	1.8% (0.5-5.9)	1.8% (0.4-7.3)	6.2% (2.4-14.7)	4.5% (0.9-19.1)
Weighs the baby	Not measured	86.3% (57.2-96.7)	85.6% (63.1-95.4)	96.3% (89.8-98.7)	88.5% (45.4-98.6)
Mother and newborn kept in same room after delivery (rooming in)	98.3% (96.1-99.3)	96.7% (88.3-99.1)	95.7% (92.8-97.5)	98.9% (96.8-99.7)	99.7% (98.4-98.8)
Baby kept skin-to-skin with mother for first hour after birth	68.8% (40.1-87.9)	39.5% (20.0-63.0)	50.3% (29.4-71.1)	72.3% (59.6-82.3)	73.2% (49.0-88.6)
Provides tetracycline ointment prophylaxis	0%	0.3% (0-2.9)	0.9% (0.2-4.6)	10.4% (4.4-22.4)	4.8% (1.5-13.9)
Administers Vitamin K to newborn	0%	0.3% (0-2.1)	0%	0%	0%
Administers chlorhexidine to the newborn cord	0%	73.7% (46.2-90.1)	81.9% (54.8-94.4)	95.0% (88.8-97.8)	91.1% (78.5-96.6)
If mother identified as HIV positive, administers ARV to newborn	100%	75.0% (20.5-97.2)	0%	0%	25.0% (2.3-82.5)
Administers antibiotics to mother	1.0% (0.3-3.6)	1.2% (0.5-2.7)	0%	1.8% (0.9-3.5)	6.0% (1.1-27.0)

For peer review only

Reporting checklist for cross sectional study.

Based on the STROBE cross sectional guidelines.

Instructions to authors

Complete this checklist by entering the page numbers from your manuscript where readers will find each of the items listed below.

Your article may not currently address all the items on the checklist. Please modify your text to include the missing information. If you are certain that an item does not apply, please write "n/a" and provide a short explanation.

Upload your completed checklist as an extra file when you submit to a journal.

In your methods section, say that you used the STROBE cross sectionalreporting guidelines, and cite them as:

von Elm E, Altman DG, Egger M, Pocock SJ, Gotzsche PC, Vandenbroucke JP. The Strengthening the Reporting of Observational Studies in Epidemiology (STROBE) Statement: guidelines for reporting observational studies.

		Reporting Item	Page Number
**Title and abstract**			
Title	#1a	Indicate the study’s design with a commonly used term in the title or the abstract	1

For peer review only

Abstract	#1b	Provide in the abstract an informative and balanced summary of what was done and what was found	2
**Introduction**			
Background / rationale	#2	Explain the scientific background and rationale for the investigation being reported	4
Objectives	#3	State specific objectives, including any prespecified hypotheses	4
**Methods**			
Study design	#4	Present key elements of study design early in the paper	4
Setting	#5	Describe the setting, locations, and relevant dates, including periods of recruitment, exposure, follow-up, and data collection	4
Eligibility criteria	#6a	Give the eligibility criteria, and the sources and methods of selection of participants.	5-7
	#7	Clearly define all outcomes, exposures, predictors, potential confounders, and effect modifiers. Give diagnostic criteria, if applicable	6-7
Data sources / measurement	#8	For each variable of interest give sources of data and details of methods of assessment (measurement). Describe comparability of assessment methods if there is more than one group. Give information separately for for exposed and unexposed groups if applicable.	5

For peer review only

Bias	#9	Describe any efforts to address potential sources of bias	n/a
Study size	#10	Explain how the study size was arrived at	5
Quantitative variables	#11	Explain how quantitative variables were handled in the analyses. If applicable, describe which groupings were chosen, and why	7
Statistical methods	#12a	Describe all statistical methods, including those used to control for confounding	7
Statistical methods	#12b	Describe any methods used to examine subgroups and interactions	n/a
Statistical methods	#12c	Explain how missing data were addressed	n/a
Statistical methods	#12d	If applicable, describe analytical methods taking account of sampling strategy	7
Statistical methods	#12e	Describe any sensitivity analyses	n/a
**Results**			
Participants	#13a	Report numbers of individuals at each stage of study—eg numbers potentially eligible, examined for eligibility, confirmed eligible, included in the study, completing follow-up, and analysed. Give information separately for for exposed and unexposed groups if applicable.	8-10
Participants	#13b	Give reasons for non-participation at each stage	5

For peer review only

Participants	#13c	Consider use of a flow diagram	8
Descriptive data	#14a	Give characteristics of study participants (eg demographic, clinical, social) and information on exposures and potential confounders. Give information separately for exposed and unexposed groups if applicable.	8
Descriptive data	#14b	Indicate number of participants with missing data for each variable of interest	9-10
Outcome data	#15	Report numbers of outcome events or summary measures. Give information separately for exposed and unexposed groups if applicable.	10-12
Main results	#16a	Give unadjusted estimates and, if applicable, confounder-adjusted estimates and their precision (eg, 95% confidence interval). Make clear which confounders were adjusted for and why they were included	10-12
Main results	#16b	Report category boundaries when continuous variables were categorized	n/a
Main results	#16c	If relevant, consider translating estimates of relative risk into absolute risk for a meaningful time period	n/a
Other analyses	#17	Report other analyses done—e.g., analyses of subgroups and interactions, and sensitivity analyses	12
**Discussion**			
Key results	#18	Summarise key results with reference to study objectives	12-13

For peer review only

Limitations	#19	Discuss limitations of the study, taking into account sources of potential bias or imprecision. Discuss both direction and magnitude of any potential bias.	14
Interpretation	#20	Give a cautious overall interpretation considering objectives, limitations, multiplicity of analyses, results from similar studies, and other relevant evidence.	14-15
Generalisability	#21	Discuss the generalisability (external validity) of the study results	14
**Other Information**			
Funding	#22	Give the source of funding and the role of the funders for the present study and, if applicable, for the original study on which the present article is based	16

None The STROBE checklist is distributed under the terms of the Creative Commons Attribution License CC-BY. This checklist can be completed online using https://www.goodreports.org/, a tool made by the EQUATOR Network in collaboration with Penelope.ai

For peer review only

BMJ Open

The provision of essential evidence-based interventions during facility-based childbirth: cross sectional observations of births in northeast Nigeria

Journal:	*BMJ Open*
Manuscript ID	bmjopen-2020-037625.R2
Article Type:	Original research
Date Submitted by the Author:	12-Sep-2020
Complete List of Authors:	Exley, Josephine; London School of Hygiene and Tropical Medicine Faculty of Infectious and Tropical Diseases, Department of Disease Control<br>Hanson, Claudia; Karolinska Institute, Public Health Sciences; London School of Hygiene and Tropical Medicine Faculty of Infectious and Tropical Diseases, Dept of Disease Control<br>Umar, Nasir; London School of Hygiene and Tropical Medicine Faculty of Infectious and Tropical Diseases, Disease Control<br>Willey, Barbara; London School of Hygiene and Tropical Medicine, Disease Control Department; London School of Hygiene and Tropical Medicine, Dept of Global Health and Development<br>Shuaibu, Abdulrahman; Gombe State Primary Health Care Development Agency<br>Marchant, Tanya; London Sch Hyg , Disease Control
**Primary Subject Heading**:	Health services research
Secondary Subject Heading:	Evidence based practice, Obstetrics and gynaecology
Keywords:	Maternal medicine < OBSTETRICS, Quality in health care < HEALTH SERVICES ADMINISTRATION & MANAGEMENT, PRIMARY CARE

SCHOLARONE™
Manuscripts

I, the Submitting Author has the right to grant and does grant on behalf of all authors of the Work (as defined in the below author licence), an exclusive licence and/or a non-exclusive licence for contributions from authors who are: i) UK Crown employees; ii) where BMJ has agreed a CC-BY licence shall apply, and/or iii) in accordance with the terms applicable for US Federal Government officers or employees acting as part of their official duties; on a worldwide, perpetual, irrevocable, royalty-free basis to BMJ Publishing Group Ltd ("BMJ") its licensees and where the relevant Journal is co-owned by BMJ to the co-owners of the Journal, to publish the Work in this journal and any other BMJ products and to exploit all rights, as set out in our licence.

The Submitting Author accepts and understands that any supply made under these terms is made by BMJ to the Submitting Author unless you are acting as an employee on behalf of your employer or a postgraduate student of an affiliated institution which is paying any applicable article publishing charge ("APC") for Open Access articles. Where the Submitting Author wishes to make the Work available on an Open Access basis (and intends to pay the relevant APC), the terms of reuse of such Open Access shall be governed by a Creative Commons licence – details of these licences and which Creative Commons licence will apply to this Work are set out in our licence referred to above.

Other than as permitted in any relevant BMJ Author's Self Archiving Policies, I confirm this Work has not been accepted for publication elsewhere, is not being considered for publication elsewhere and does not duplicate material already published. I confirm all authors consent to publication of this Work and authorise the granting of this licence.

For peer review only

The provision of essential evidence-based interventions during facility-based childbirth: cross sectional observations of births in northeast Nigeria

Josephine Exley[1], Claudia Hanson[1,2], Nasir Umar[1], Barbara Willey[3], Abdulrahman Shuaibu[4], Tanya Marchant[1*]

[1] Department of Disease Control, London School of Hygiene & Tropical Medicine, UK

[2] Department of Public Health Sciences–Global Health, Karolinska Institutet, Stockholm, Sweden

[3] Department of Infectious Disease Epidemiology, London School of Hygiene & Tropical Medicine, UK

[4] The Executive Secretary, Gombe State Primary Health Care Development Agency, Nigeria

Correspondence to:
Josephine Exley
LSHTM
Keppel Street
London
WC1E 7HT
United Kingdom
Josephine.Exley@lshtm.ac.uk

Word Count: 4,764

For peer review only

For peer review only - http://bmjopen.bmj.com/site/about/guidelines.xhtml

Abstract

Objectives:

To measure the provision of evidence-based preventive and promotive interventions to women, and subsequently their newborns, during childbirth in a high-mortality setting.

Design and participants:

Cross-sectional observations of care provided to women, and their newborns during the intra- and immediate postpartum period using a standardised checklist capturing healthcare worker behaviours regarding lifesaving and respectful care.

Setting:

Ten primary healthcare facilities in Gombe State, northeast Nigeria. The northeast region of Nigeria has some of the highest maternal and newborn death rates globally.

Main outcome measures:

Data on 50 measures of internationally recommended evidence-based interventions and good practice.

Results:

1,875 women were admitted to a health facility during the observation period; of these, 1,804 gave birth in the facility and did not experience an adverse event or death. Many clinical interventions around the time of birth were routinely implemented, including provision of uterotonic (96% [95%CI 93-98]), whereas risk-assessment measures, such as history-taking or checking vital signs were rarely completed: just 2% (95%CI 2-7) of women had temperature taken and 12% (95%CI 9-16) were asked about complications during pregnancy.

Conclusions:

The majority of women did not receive the recommended routine processes of childbirth care they and their newborns needed to benefit from their choice to deliver in a health facility. In particular, few benefited from even basic risk-assessments, leading to missed opportunities to identify risks. To continue with the recommendation of childbirth care in primary healthcare in high mortality settings like Gombe it is crucial that birth attendant capacity, capability and prioritisation processes are addressed.

For peer review only - http://bmjopen.bmj.com/site/about/guidelines.xhtml

Strengths and limitations of this study

- This study has a large sample size; over 1,850 women were observed across five rounds of data collection.
- The relative consistency overtime and low levels of implementation of many measures suggest that any impact of being observed was minimal.
- Observers received training, used a structured checklist and were overseen by a clinical supervisor to improve the reliability of observations.
- The study protocol prioritised observation of events closest to birth, as such 40% of women were not observed during the first stage of labour.
- This study was completed in the 10 primary healthcare facilities with the highest volume of births in Gombe State and therefore results are not representative of all facilities.

For peer review only

Introduction

Global efforts to reduce preventable maternal and newborn mortality have focused on skilled attendance at birth. This has resulted in marked increases in coverage of births with a skilled birth attendant, mainly operationalised through childbirth in facilities. However, these increases have not been accompanied by the anticipated improvements in maternal and newborn outcomes in many low and middle income countries [1-4], prompting a closer examination of the quality of care provided [5-7]. A growing body of evidence from low income settings highlights low provider skills and limited facility capability to provide good-quality care at birth [1 8-10]. A recent review found large declines in the proportion of individuals estimated to have skilled birth attendance when some measure of quality was taken into account [11].

Good quality of care includes the timely and appropriate use of evidence-based clinical and non-clinical interventions that are acceptable to women [12-14]. The World Health Organisation (WHO) has developed evidence-based guidelines around intrapartum care, which have the potential to support healthcare providers to identify gaps in the quality of care and improve the provision and experience of care [15]. A continuing challenge, however, is that detailed evidence on the extent to which the recommended interventions are practised during routine childbirth in health facilities in low-income countries is scarce [1 16]. Available studies have predominantly focused on readiness for quality such as availability of drugs, supplies and other inputs, while neglecting processes of care [16]. In part, this is because these data are made available through surveys similar to the Service Provision Assessments [17], or through routine health information systems - information sources that include very little process quality data. Household surveys, including the Demographic and Health Survey and the Multiple Indicator Cluster Survey, do not collect extensive data on the content of care during childbirth, in part because evidence suggests women's self-reports have low validity [18 19]. This lack of data on coverage of evidence-based care during childbirth impedes decision making around possible solutions. Although not a large-scale measurement solution, observations provide useful insight in to the behaviours of healthcare workers and could support strategies to improve care.

In this study of birth observations, we aimed to examine the maternal and newborn outcomes experienced by all women admitted for childbirth and postpartum haemorrhage in a sample of primary healthcare facilities in Gombe State, Nigeria. For women who had an uncomplicated labour, we evaluated the provision of evidence-based care provided to women, and subsequently their newborns, from initial assessment up to one hour postpartum.

Methods

We conducted direct observations of childbirth care in 10 primary healthcare facilities, in Gombe State, Nigeria, approximately every six months over a two-year period between June 2016 and August 2018.

Study setting

Gombe State is one of six states in northeast Nigeria, it has an area of 20,265km^2 and a population of 2,857,042 [20]. Over 80% of the population live in rural areas and are reliant on subsistence farming as their primary source of income [21]. The northeast region of Nigeria has some of the highest maternal and newborn death rates globally, estimated at 1,549 per 100,000 live births in 2015 and 33 per 1,000 live births in 2017, respectively [22 23].

Access to maternal healthcare services is relatively low in Gombe State. In 2018, 46% of women in the State reported at least one antenatal care visit from a doctor, nurse, midwife or

For peer review only - http://bmjopen.bmj.com/site/about/guidelines.xhtml

nurse/midwife and 28% delivered in a health facility [24]. Over 70% of facility deliveries, in 2018, took place in a rural primary healthcare facility [25]. Recent work in Gombe on the drivers of attending a facility for childbirth found that health system conditions including availability of staff, drugs and supply, and a clean environment had the biggest influence on respondents' decision around where to give birth [26].

Healthcare is predominantly delivered via a network of rural primary healthcare clinics run by the Gombe State Primary Healthcare Development Agency (GSPHCDA). In 2017, 460 primary healthcare clinics and 26 referral facilities provided childbirth services [27]. In primary healthcare facilities care is typically delivered by lower cadres of healthcare workers, for example, community health extension workers (CHEWS), junior CHEWS and health officers [28 29]. In response to the shortage and uneven distribution of healthcare workers, under its task-shifting and task-sharing policy for essential healthcare services, Nigeria classifies CHEWs as skilled birth attendants [30].

Primary healthcare facilities in Gombe are poorly resourced, often lacking essential supplies and commodities to provide basic maternal and newborn health care [31-33]. Led by the Gombe State Primary Healthcare Development Agency, since 2016 intense NGO activity has been ongoing in 57 primary healthcare facilities across Gombe State, aimed at increasing the quality of care [34 35]. Interventions include training of CHEWs in all aspects of skilled birth attendance and basic emergency obstetric care, and improving the supply of essential maternal and newborn health commodities [36]. These facilities provide basic emergency obstetric and newborn care. Emergency care and complicated cases from these health facilities are referred to referral facilities. None of the 57 primary healthcare facilities have a medical doctor, 4% have at least one nurse and 19% have at least one midwife.[37]

Sampling methods have been described in detail elsewhere [19 32]. Briefly, in November 2015, 10 primary healthcare facilities were selected from the 57 facilities for an in-depth assessment of quality of care. To achieve a sufficiently large number of observations and minimise the duration of data collection, the 10 primary healthcare facilities with the highest number of births in the preceding six months, as recorded in the maternity register, were purposively selected. The mean number of births per month in the 10 primary healthcare facilities was 15.7 (standard deviation [sd] 12.0), compared to 4.3 (sd 6.3) births per facility per month across Gombe State as a whole [19].

Data collection

Five rounds of data collection took place over the two-year study period. Each round lasted three weeks, during which observers aimed to collect data from a total of around 350 women. Two trained female observers (local midwives, not employed by the facility) and one clinical supervisor were assigned to each facility. Observers worked in 8- or 12-hour shifts to provide near continual data collection during the period. Depending on the observation team's work schedule, the first point of contact for any observation may have been during initial assessment of a newly admitted pregnant woman or at a later stage of labour. Observers aimed to observe all women who were admitted irrespective of the cadre of the attending healthcare worker, but they prioritised observing women during the second and third stage of labour and immediately postpartum rather than observing women earlier in the process. Observers stayed continuously with women from the first point of contact until the first hour after birth. The healthcare worker observed may have been different at different timepoints in the same facility. The clinical supervisor was always available onsite but not present in the delivery room.

For peer review only - http://bmjopen.bmj.com/site/about/guidelines.xhtml

A structured clinical observation checklist, administered on a Lenovo A3300 tablet using CSPro version 7.0 (United States Census Bureau and ICF Macro, Suitland, MD, USA), was used to record the processes of care and birth attendant-client interactions and client characteristics. The content of the checklist was developed from the USAID-funded Maternal and Child Health Integrated Program's tool for observing vaginal births and the following complications: postpartum haemorrhage, pre-eclampsia/eclampsia and newborn asphyxia [38]. The checklist was piloted and modified to the Gombe context.

All women attending the facility in active labour or experiencing postpartum haemorrhage were invited to participate at the time of admission. All potential participants were provided with a study information sheet and a consent form in English and Hausa. Taking care to include any support persons accompanying potential participants, the observer read the information sheet, explained the purpose of the study, the risks and benefits of participating and answered questions before seeking written consent from the woman and verbal consent from the healthcare worker attending. Women who were not able to write their name were asked to provide a thumb print on the consent form. Participation was voluntary and participants were free to withdraw at any time.

Before each round of data collection, observers underwent four days of training on how to conduct unobtrusive observations, the safety and confidentiality protocols and how to ensure consistency of rating between observers. Throughout the observation period, clinic supervisors conducted spot checks of observers and data to provide ongoing quality assurance.

Observers were required to prioritise the safety of the mother and newborn; protocols were established on the actions to take during any life-threatening events. This included immediately stopping the observation activity and calling for the clinical supervisor who could advise the attending healthcare worker. A formal report detailing any actions and decisions made, was made available to the Executive Secretary of the GSPHCDA. Where data collection was stopped, observations were excluded from the study.

Defining provision of evidence-based care

For this analysis, the content of the clinical observation checklist was mapped against current recommendations for high quality mother and newborn care [13 15 39-42]. Fifty measures were identified (Box 1), grouped into four organising categories based on the stage of childbirth: (1) initial assessment; (2) first stage of labour; (3) second and third stage of labour; (4) immediate newborn and postpartum care.

Box 1 Measures of evidence based childbirth care included in this analysis

History taking and initial assessment
• Checks client card or asks client her age, length of pregnancy, and parity
• Asks whether woman has experienced any complications during current pregnancy
• If woman has had any previous pregnancies, asks about complications during previous pregnancies
• Checks client card or asks client her HIV status
• Washes his/her hands with soap and water or uses disinfectant before initial any examination
• Takes temperature
• Takes blood pressure
• Checks foetal heart rate with fetoscope/doppler/ultrasound
• Performs vaginal examination

For peer review only - http://bmjopen.bmj.com/site/about/guidelines.xhtml

For peer review only

- Encourages the women to have a support person present during labour and birth
- Explains procedures to woman (support person) before proceeding
- Asks women (and support person) if she has any questions

First stage of labour

- Partograph used to monitor labour
- Washes his/her hands with soap and water or uses antiseptic prior to any examination of woman
- Wears high-level disinfected or sterile surgical gloves
- A support person (or companion) for mother is present at some point during labour
- At least once, explains what will happen in labour to woman (support person)
- At least once, encourages woman to consume fluids/food during labour
- Drapes woman (one drape under buttocks, one over abdomen)
- At least once, encourages/assists woman to ambulate and assume different positions during labour
- Following items of equipment laid out in preparation for birth:
  - Newborn face mask size 0 or 1 and self-inflating ventilation bag (250 or 500 mL)
  - Suction bulb
  - Disposable cord ties or clamps
  - Sterile scissors or blade
  - At least two cloths/blankets (one to dry; one to cover)

Second & third stage of labour

- Assisted by more than one healthcare worker at one point during labour
- Mother gave birth in lithotomy position (on back)
- As baby's head is delivered, supports perineum
- Administers uterotonic
- Timing of uterotonic
- If care provided by a skilled birth attendant, applies traction to the cord while applying suprapubic counter traction
- Assesses completeness of the placenta and membranes
- Assesses for perineal and vaginal lacerations
- A support person (companion) for mother is present at birth

For peer review only - http://bmjopen.bmj.com/site/about/guidelines.xhtml

Immediate newborn and postpartum care
• Drying baby immediately after birth with towel
• Baby placed skin-to-skin with mother
• Bathing delayed for at least one hour
• Ties or clamps cord when pulsations stop, or by 2-3 minutes after birth (not immediately after birth)
• Cuts cord with clean blade or clean scissors
• Breastfeeding initiated within first hour
• Takes mother's vital signs within the first 15 minutes after birth
• Administers antibiotics to mother postpartum
• Checks baby's temperature within the first 15 minutes after birth
• Baby weighed
• Mother and newborn kept in same room after birth (rooming-in)
• Baby kept skin-to-skin for first hour
• Administers Vitamin K to newborn
• Provides tetracycline eye ointment prophylaxis
• Administers chlorhexidine to the newborn cord
• If the mother is HIV positive, administers ARVs to newborn

Inclusion criteria

Data from the five data collection periods were combined into a single dataset. Observations were excluded from the dataset if the woman's outcome was not recorded. For all women observed, we mapped the different pathways from admission to the facility (childbirth or postpartum haemorrhage event) to their outcome. For women who experienced an uncomplicated labour the outcome of their baby was also mapped. An uncomplicated labour was defined as a woman who was sent to the ward for recuperation or discharged home after birth and who did not experience an adverse event to her own health (referral, postpartum haemorrhage or pre-eclampsia/eclampsia) or death.

For the analysis of the provision of essential evidence based care, our population of interest was women with an uncomplicated labour and detailed information on their care and that of their newborn are included here. Women who were admitted but experienced an adverse event or death were excluded from the analysis because of their individual medical needs. For measures related to newborn care the analysis was further restricted to newborns recorded as being alive and who did not require resuscitation care or were not referred to another facility.

Analysis

For each measure, percent frequencies and 95% confidence intervals were calculated, adjusted for clustering by primary healthcare facility and stratified by time point using the svyset and svy commands in STATA version 15.1 (StataCorp, 2017, College Station, TX). Results are presented graphically by time point to highlight any variability and the average across all five time points is presented in the text.

Ethical approval

Ethical approval for this study were obtained from the London School of Hygiene & Tropical Medicine (reference 14091) and the Health Research Ethics Committees for Nigeria (reference NHREC/01/01/2007) and Gombe State (reference ADM/S/658/Vol. II/66).

For peer review only

For peer review only - http://bmjopen.bmj.com/site/about/guidelines.xhtml

Patient and Public Involvement

Patients and the public were not involved in the design, conduct, reporting or dissemination plans of our research. Observations were recorded in English and pre-testing completed in health facilities by staff.

Results

In total 1,875 women were admitted to a facility during the five observations periods. The median age of the women was 24 years (interquartile range [IQR] 20–29) and for 19% it was their first birth: median parity 2 (IQR 1–5). The median gestational age of women on admission was 39 weeks (IQR 38–39); 6% of women had a gestational age of less than 37 weeks and for 21% of women gestational age was not recorded on their client card and/or they did not know. At the start of the observation period, 11% of women were attended by a skilled birth attendant (doctor, midwife, nurse), 39% by a CHEW or junior CHEW and 50% by an 'other' birth attendant. 'Other' included environmental health officers/technicians/assistants (43%), health attendant/assistant (43%), traditional birth attendants (4%), community health officer (1%) and other (9%).

We first present the outcomes for all 1,875 women who were admitted to the facility, followed by the coverage of the provision of evidence based care measures, outlined in Box 1, for the 1,804 women who had an uncomplicated labour and the 1,635 babies born to these women who did not experience an adverse event or death. The full table of results disaggregated by time-point is presented in supplementary material.

Outcomes

Figure 1 presents the outcomes for the 1,875 women admitted to a facility during the five observation periods. Nine women were admitted with postpartum haemorrhage and 1,866 were admitted for childbirth. Fourteen women admitted for childbirth were referred to another facility during labour. The main reason for referral was prolonged labour; three women were referred with severe pre-eclampsia and one had eclampsia. For the four women diagnosed with severe pre-eclampsia/eclampsia during labour, insufficient assessment and monitoring of the woman was noted by observers: "*there were delays in needed treatment. No observations undertaken during the first hour of admission to detect disease condition and take appropriate action",* whilst another observed *"woman was admitted for normal labour but only vaginal examination was observed without physical nor vital signs or urine test".*

Of the 1,852 women who gave birth in the facility, all delivered vaginally with one woman recorded as requiring an assisted delivery; caesarean sections are not available in primary healthcare facilities in this setting. Over half of births occurred on a weekday (56% [1,031/1,852]) between 7am and 7 pm (58% [1,067/1,852]). Two per cent (34/1,852) of deliveries were multiple births.

Postpartum, 45 women experienced an adverse event and three women died: the mortality rate was 1.6 per 1,000 women. Five women were referred intrapartum due to complications associated with multiple births and six women were referred postpartum. Of the seven women referred postpartum, two women with eclampsia were referred because their condition could not be stabilised. Both women experienced convulsions and lost consciousness. They both received magnesium sulphate before referral: one woman's baby was born alive and referred with the mother, the other was a fresh stillbirth. Three women were referred following a

For peer review only - http://bmjopen.bmj.com/site/about/guidelines.xhtml

postpartum haemorrhage event; all received oxytocin in the facility before referral and none had severe postpartum haemorrhage (abnormal bleeding of more than 1000 mL): one woman's condition was reported not to be stable and the baby required resuscitation before referral. The other two women were reported to be stable and their baby was referred with them. The remaining woman referred postpartum was referred because "*mother had a retain placenta, mentor and birth attendant tried everything but it failed*". The newborn was referred with their mother.

For the babies born to women who had an uncomplicated labour, 96 received resuscitation care of whom 19% subsequently died. Overall, 79 babies died: the perinatal mortality rate was 44.1 per 1,000 newborns (Figure 1). For babies born to women who experienced an adverse an event or died, 19% (9/48) were referred and four died: the perinatal morality rate among this group was 83.3 per 1,000 newborns.

Provision of evidence-based care

All 1,804 women who had an uncomplicated labour were observed during the second and third stage of labour. Observers were required to prioritise the second and third stage of labour so the number of women and newborns observed during other stages of childbirth varied (Table 1).

Table 1 Proportion of all women admitted for childbirth who were observed, disaggregated by stage of care

Stage of care	Percentage observed	
	Women	Newborns
Initial assessment and history taking	99.8% (1,801/1,804)	n/a
First stage of labour	59.3% (1,069/1,804)	
Second & third stage of labour	100% (1,804/1,804)	
Immediate newborn & postpartum care	94.1% (1,697/1,804)	98.9% (1,617/1,635)

History taking and Initial assessment

In total, 1,801 women were observed during the initial assessment and history taking. Birth attendants were routinely observed to check the women's record for or, if not available, asked women's age, length of pregnancy and parity; 80% (95%CI 73-86), see Figure 2. However, other aspects of history taking including asking about complications during both current and, if relevant, previous pregnancies were very low: 12% (95%CI 9-16) and 18% (95%CI 15-23), respectively. The most common complications that birth attendants asked about for the current pregnancy were fever (37% [81/218]), vaginal bleeding (32% [70/218]) and severe abdominal pain (25% [54/218]).

The majority of women were encouraged to have a support person present during labour and birth (63% [95%CI 53–72]). While around two thirds of birth attendants explained procedures to woman (and their support person) before proceeding (66% [95%CI 57–74]), less than a third of women (and their support person) were asked if they had any questions (32% [95%CI 25–40]).

Vaginal examination was almost universal (98% [95%CI 97-99]) but the proportion of women who had their temperature and blood pressure measured, and the foetal heart rate checked was low: 4% (95%CI 2-7), 30% (95%CI 23-39) and 22% (95%CI 16–29), respectively (Figure 2). Few birth attendants washed their hands with soap and water or antiseptic before examining women (28% [95%CI 21-37]).

For peer review only - http://bmjopen.bmj.com/site/about/guidelines.xhtml

First stage of labour

In total, 1,069 women were observed during first stage of labour. Partograph was used to monitor labour in just 24% (95%CI 18-32) of cases (Figure 3). Data were not collected on whether there was a delay in the progress of labour but 17% (95%CI 12-24) of women had their labour augmented with oxytocin and 6% (95%CI 4-9) of women's membranes were artificially ruptured.

The median number of vaginal examinations undertaken during the first stage of labour was one (IQR 1-2). Birth attendants were observed to wash their hands with soap & water or use antiseptic 39% (95%CI 30-49) of the time prior to any examination of woman, although a greater proportion were observed to wear high-level disinfectant or surgical gloves when performing the vaginal exam 76% (95%CI 64-85).

Seventy two per cent (95%CI 63–79) of women had a support person present at some point during labour (Figure 3). Just under half of birth attendants were observed to explain to the woman (and their support person) what will happen during labour (49% [95%CI 42–56]). The majority of birth attendants were observed to encourage the woman to consume fluids/food at least once during labour (89% [95%CI 85-92]) and to encourage or help woman to ambulate and assume different positions (73% [95%CI 66–80]). During more than half of births observed, birth attendants were observed to drape women with a cloth, one under the buttocks and one over the abdomen (59% [95%CI 48–69]).

Data were collected on whether clean cloths/blankets, tie or cord clamp, sterile blade to cut cord, suction device and bag and mask (either size 0 or 1) were laid out in preparation for birth. Cord clamps, sterile blade and at least two cloths/blankets were available for over 90% of women (Figure 3). However, preparation of a bag and mask was substantially lower at 40% (95%CI 31-50).

Second and third stage of labour

All 1,804 women were observed during the second and third stage of labour. Just over half of women observed were assisted at some point during the second and third stage of labour by more than one healthcare worker (57% ([95%CI 50-63]) and 36% (95%CI 27-45) had a support person present (Figure 4). Women universally gave birth in the lithotomy position (97% [95% CI 93-98]). The use of episiotomy was very low (1% [95%CI 1-2]). Other inappropriate practices were also low: 3% (95%CI 1-7) fundal pressure, 1% (95%CI 0-2) excessive stretching of perineum and less than 1% had lavage of uterus.

The use of prophylactic uterotonic drugs immediately after birth was universal (96% [95%CI 93-98]), and 65% (95%CI 56-73) of birth attendants checked for the presence of a second baby before administering a uterotonic. Of women that received a uterotonic, 58% received oxytocin (999/1,735) and 42% misoprostol (733/1,735). Fourteen per cent (95%CI 9-20) of deliveries where uterotonics were administered were given within 1 minute of birth and for 52% (95%CI 44-57) of women a uterotonic was administered more than 3 minutes after birth. Immediately following the delivery/expulsion of the placenta, 75% (95%CI 69-80) of birth attendants were observed to perform uterine massage.

The use of controlled cord traction was consistently undertaken by all cadres of birth attendants: skilled 91% (95%CI 83-96), CHEW 85% (95%CI 76-92) and 'other' birth attendant 84% (95%CI 77-89).

For peer review only - http://bmjopen.bmj.com/site/about/guidelines.xhtml

Following the birth of the placenta, 49% (95%CI 39-58) of birth attendants were observed to assess the completeness of placenta and membranes, a considerably higher proportion (87% [95%CI 79-92]) assessed for perineal and vaginal lacerations.

Immediate newborn and postpartum care

During the immediate postpartum period, 1,617 newborns and 1,697 women were observed. The majority of newborns received all three elements of thermal care (immediate drying, skin-to-skin and not bathing in the first hour) and clean cord care (ties or clamps cord when pulsation stop or within 2-3 minutes after birth and cuts cord with clean scissors or blade): 71% (95%CI 60-80) and 92% (95%CI 89-95), respectively (Figure 5). Eighty-six per cent (95%CI 75-93) of newborns had chlorohexidine applied to their cord within the first hour of birth. However, breastfeeding was initiated within the first hour after birth in just under half of newborns: 49% (95%CI 39-59).

Eighty-nine per cent (95%CI 79-95) of newborns were weighed. Of these infants, 95% (1,122/1,182) weighed more than 2.5kg (Figure 5). Mothers and newborns were universally kept in the same room after birth (rooming in), but only 61% (95%CI 51-71) were kept skin-to-skin during the first hour.

For the fifteen babies born to mothers known to have HIV, 33% (95%CI 14-60) were observed to receive antiretroviral therapy. The proportion of newborns on postnatal care wards who received a vitamin K injection, tetracycline eye ointment and had their temperature checked within 15 minutes after birth was close to zero throughout the study period (Figure 5): 0.1% (95%CI 0-0.4), 4% (95%CI 2-7) and 2% (95%CI 1-5), respectively.

Just 3% (95%CI 2-6) of women had their vital signs checked 15 minutes after birth and 2% (95%CI 1-5) of women received antibiotics.

Discussion

Our observations in ten primary healthcare facilities in northeast Nigeria indicated that, while some essential processes of childbirth care were performed for almost all women and newborns, the proportion of women who consistently received evidence-based care during uncomplicated labour was low. Three percent of women (48/1,852) who gave birth at the facility experienced major risks and complications while at the facility, including six severe pre-eclampsia/eclampsia and six twin deliveries. Three women died. There were large numbers of perinatal deaths and newborn referrals among this group of women. Further, among women who we defined as having an uncomplicated labour 79 perinatal deaths were recorded, of which almost three-quarters were intrapartum stillbirths.

Our study highlights substantial variation in the implementation of recommended evidence-based interventions both within and across the different stages of childbirth: during all four stages, fewer than half of measures reached more than 80% of women. Implementation was highest for clinical interventions at the time of birth that have received international attention, for example, the provision of prophylactic uterotonic and newborn thermal and clean cord care. Implementation was lowest for measures designed as risk assessments, for example, history-taking or checking vital signs. All measures related to risk assessment during the initial assessment (asks about complications in current and previous pregnancies, takes temperature and blood pressure, checks foetal heart rate) were completed for fewer than 30% of women observed.

For peer review only - http://bmjopen.bmj.com/site/about/guidelines.xhtml

The evidence-based care measures included in this study were based on WHO guidelines for essential intrapartum care [15]: their implementation should be covered in basic pre-service training for midwives, nurses and CHEWs in this setting; and they require only basic supplies of equipment. However, half of all observed births were attended by 'other' staff who would be defined as unskilled birth attendants both globally and in the Nigerian context: not doctors, nurses or midwives, nor CHEWs who have received additional training under Nigeria's task-shifting policy. Even so, far fewer than half of the women received many measures, raising questions even about the behaviours of birth attendants who have received training [43]. Further, although some variation was observed over time, in general, coverage of individual measures remained relatively constant throughout the observation period, including for a number of practices that are no longer recommended by WHO, for example, uterine massage [44]. Taken together, this study suggests that there may be limited opportunities for birth attendants to keep their knowledge up-to-date and enhance their skills once in post. Implementation research is needed to identify mechanisms to continuously support and improve healthcare worker practices, such as the use of checklists and in-service supervision and coaching, which has shown potential in other settings to improve uptake of and adherence to essential birth practices [39 45].

A potential alternative reason for the variation in care across the continuum is that some birth attendants were working alone for extended periods. In these circumstances, birth attendants may prioritise interventions during the third stage of labour and immediate newborn care. Higher workload, as a result of staff shortages, has been found to limit healthcare workers time for history-taking, thorough assessment of women, and ability to provide timely care [37 46]. Further, when working alone, birth attendants struggle to implement multiple recommended interventions simultaneously, for example, administering uterotonic and providing essential newborn care. While administering uterotonic was universal, less than half of women received a uterotonic within three minutes of birth. The importance of the exact timing of the application of uterotonic is not well-established, and as such, it is not clear whether this should be prioritised over immediate newborn care interventions [47 48] and clearer guidelines may be needed if it is important to prioritise certain components of care that cannot be done simultaneously by a single healthcare worker [49].

Poor assessment of women and newborns before and after birth has previously been documented in India and other African settings [50-52]. Observations of WHO-recommended practices for screening of pre-eclampsia/eclampsia in six sub-Saharan African countries, similarly found that a low proportion of women admitted to labour and delivery services were asked about danger signs, but substantially more women (77%) had their blood pressure checked on admission [51]. Low implementation of evidence-based care measures during the initial assessment and postpartum period indicate potential missed opportunities to identify and manage complications, as evident in the referral cases. Referrals post- and intrapartum included women with high-risk pregnancies such as severe pre-eclampsia/eclampsia, breech position and twins who should have been referred to a higher level of care [53]. This study has identified proper risk assessment at the time of birth as a priority, not least because, in this setting, only 37% of women attended at least four antenatal care visits and 10% of women and 7% of newborns received a postnatal check within 2-days of birth in 2015 [54].

Poor quality of care has been acknowledged as a critical roadblock in Nigeria's attempt to reach universal health coverage. The Federal government has committed to strengthening Nigeria's health system, particularly primary healthcare, and specifically to accelerate the

For peer review only - http://bmjopen.bmj.com/site/about/guidelines.xhtml

reduction of maternal and neonatal mortality by expanding access to, and quality of, maternal and child health services [55]. The implementation of the national strategy is supported by Nigeria's participation in the Network for Improving Quality of Care for Maternal, Newborn and Child Health (Quality of Care Network) [56]. The processes of care prioritised in these strategies focus on clinical interventions at the time of birth, including use of uterotonic drugs, skin-to-skin and chlorhexidine for umbilical cord care – interventions that were found to be routinely well implemented in this low resource primary healthcare setting. This study's findings suggest if these policies are to have an impact they need to extend their focus to also include basic risk-assessment. Further, while they aim to support healthcare workers at the health facilities through quality improvement cycles, clinical mentoring and peer-to-peer learning they may also need to consider the quality of pre-service training.

Strengths and Limitations

This study provides unique insight into the provision of evidence-based practices during childbirth and highlights clear areas for action in this setting, actions that are also likely to be relevant elsewhere. A particular strength was the relatively large sample size from multiple time points although, due to the study protocol to prioritise events closest to birth, over 40% of women were not observed during the first stage of labour. The missing observations are not anticipated to impact on findings as non-observation was random, with women who were not observed during the first stage of labour unlikely to differ systematically from those who were. Being observed may have impacted on birth attendants' behaviour; evidence suggests that being observed can positively improve behaviours although any change is likely to be short lived [57 58]. The sample size was estimated to be sufficiently large to reduce the impact of any potential Hawthorne effect and the relative consistency overtime and the low levels of implementation of many measures suggest that any effect of being observed was minimal.

This study was completed in the 10 primary healthcare facilities with the highest volume of births in Gombe State and is not therefore representative of all primary healthcare facilities. It is anticipated that these facilities represent the 'best' care available at the primary level and therefore findings are likely to overestimate the provision of evidence-based care available to the wider population. These findings support the growing body of evidence that giving birth in a primary healthcare facility might not be sufficient to ensure the effective care of women and newborns [1 4 59], and raise questions about the safety and quality of rural primary healthcare facilities that have low-volume of deliveries [16 60].

Conclusions

The recommendation for women to deliver in health facilities is designed to improve birth outcomes. This study of clinical observations of labour and the immediate postpartum period in primary facilities in Gombe State has revealed that, while some processes of clinical care were well adhered to, most women delivering in primary healthcare facilities do not receive the complete repertoire of childbirth care that they and their newborns needed to benefit from their choice to deliver in a health facility. In particular, few women or newborns benefited from even basic risk assessments, leading to missed opportunities to identify risks and consequently late referrals and deaths. To continue with the recommendation of childbirth care in primary healthcare in high mortality settings like Gombe it is crucial that birth attendant capacity, capability and prioritisation processes are purposively addressed.

For peer review only

For peer review only - http://bmjopen.bmj.com/site/about/guidelines.xhtml

Supporting information

Acknowledgements
The authors would like to thank Data Research and Mapping Consult for coordinating the data collection, the Gombe State Primary Health Care Development Agency, Gombe State Ministry of Health and our partners Society for Family Health and Pact Nigeria for their support in carrying out this study. We would like to thank all the participants who contributed to our study.

Ethical approval and consent to participate
This research was conducted with approval from the Federal Ministry of Health Abuja, Nigeria, the State Ministry of Health Gombe State, Nigeria and the London School of Hygiene & Tropical Medicine (reference 12181).

Funding
This work is part of the IDEAS (Informed Decisions for Actions to improve maternal and newborn health) project. IDEAS is funded through a grant from the Bill & Melinda Gates Foundation to the London School of Hygiene & Tropical Medicine. Gates Global Health Grant Number: OPP1149259/INV-007644. The funder had no role in study design, data collection and analysis, decision to publish or preparation of the manuscript.

Competing interests
We have read the BMJ policy and the authors declare that they have no competing interests.

Authors' contributions
JE conducted the analysis and drafted the manuscript. CH supported the refinement of our definition of evidence-based care and reviewed the manuscript for intellectual content. NU, BW and AS all critically reviewed the manuscript for intellectual content. TM conceived the study and drafted the manuscript. All authors have read and approved the final version of the manuscript.

Data sharing
The data are available from the principal investigator of the IDEAS project and co-author for this manuscript, Dr Tanya Marchant ORCID id 0000-0002-4228-4334. Reuse permitted on request.

Patient consent and confidentiality
Participation in the study was voluntary and participants were free to withdraw from the study at any time without giving a reason. Researchers explained the purpose of the study and answered any questions participants had. The free and informed consent of all women and birth attendants observed was obtained before participating.

Patient and public involvement
Patients or the public were not involved in the design, conduct, reporting or dissemination plans of our research. Observations were recorded in English and pre-testing completed in health facilities by staff.

For peer review only

For peer review only - http://bmjopen.bmj.com/site/about/guidelines.xhtml

References

1. Campbell OM, Calvert C, Testa A, et al. The scale, scope, coverage, and capability of childbirth care. *Lancet (London, England)* 2016;388(10056):2193-208. doi: 10.1016/s0140-6736(16)31528-8 [published Online First: 2016/09/20]
2. Gabrysch S, Nesbitt RC, Schoeps A, et al. Does facility birth reduce maternal and perinatal mortality in Brong Ahafo, Ghana? A secondary analysis using data on 119 244 pregnancies from two cluster-randomised controlled trials. *The Lancet Global health* 2019;7(8):e1074-e87. doi: 10.1016/s2214-109x(19)30165-2 [published Online First: 2019/07/16]
3. Randive B, Diwan V, De Costa A. India's Conditional Cash Transfer Programme (the JSY) to Promote Institutional Birth: Is There an Association between Institutional Birth Proportion and Maternal Mortality? *PloS one* 2013;8(6):e67452. doi: 10.1371/journal.pone.0067452 [published Online First: 2013/07/05]
4. Scott S, Ronsmans C. The relationship between birth with a health professional and maternal mortality in observational studies: a review of the literature. *Tropical medicine & international health : TM & IH* 2009;14(12):1523-33. doi: 10.1111/j.1365-3156.2009.02402.x [published Online First: 2009/10/02]
5. Kruk ME, Gage AD, Joseph NT, et al. Mortality due to low-quality health systems in the universal health coverage era: a systematic analysis of amenable deaths in 137 countries. *Lancet (London, England)* 2018;392(10160):2203-12. doi: 10.1016/s0140-6736(18)31668-4 [published Online First: 2018/09/10]
6. Kruk ME, Gage AD, Arsenault C, et al. High-quality health systems in the Sustainable Development Goals era: time for a revolution. *The Lancet Global Health* 2018;6(11):e1196-e252. doi: 10.1016/S2214-109X(18)30386-3
7. World Health Organization. Strategies towards ending preventable maternal mortality (EPMM). Geneva: World Health Organization 2015:44 p. Executive summary published as WHO/RHR/15.03.
8. Merali HS, Lipsitz S, Hevelone N, et al. Audit-identified avoidable factors in maternal and perinatal deaths in low resource settings: a systematic review. *BMC pregnancy and childbirth* 2014;14:280. doi: 10.1186/1471-2393-14-280 [published Online First: 2014/08/19]
9. Hobbs AJ, Moller AB, Kachikis A, et al. Scoping review to identify and map the health personnel considered skilled birth attendants in low-and-middle income countries from 2000-2015. *PloS one* 2019;14(2):e0211576. doi: 10.1371/journal.pone.0211576 [published Online First: 2019/02/02]
10. Radovich E, Benova L, Penn-Kekana L, et al. 'Who assisted with the delivery of (NAME)?' Issues in estimating skilled birth attendant coverage through population-based surveys and implications for improving global tracking. *BMJ global health* 2019;4(2):e001367. doi: 10.1136/bmjgh-2018-001367 [published Online First: 2019/05/30]
11. Amouzou A, Leslie HH, Ram M, et al. Advances in the measurement of coverage for RMNCH and nutrition: from contact to effective coverage. *BMJ global health* 2019;4(Suppl 4):e001297. doi: 10.1136/bmjgh-2018-001297 [published Online First: 2019/07/13]
12. Tuncalp, Were WM, MacLennan C, et al. Quality of care for pregnant women and newborns-the WHO vision. *BJOG : an international journal of obstetrics and gynaecology* 2015;122(8):1045-9. doi: 10.1111/1471-0528.13451 [published Online First: 2015/05/02]
13. Miller S, Abalos E, Chamillard M, et al. Beyond too little, too late and too much, too soon: a pathway towards evidence-based, respectful maternity care worldwide. *Lancet (London, England)* 2016;388(10056):2176-92. doi: 10.1016/s0140-6736(16)31472-6 [published Online First: 2016/09/20]
14. Oladapo O, Tunçalp Ö, Bonet M, et al. WHO model of intrapartum care for a positive childbirth experience: transforming care of women and babies for improved health and

For peer review only - http://bmjopen.bmj.com/site/about/guidelines.xhtml

wellbeing. *BJOG: An International Journal of Obstetrics & Gynaecology* 2018;125(8):918-22. doi: 10.1111/1471-0528.15237
15. World Health Organization. WHO recommendations: intrapartum care for a positive childbirth experience. Geneva: World Health Organization, 2018.
16. Kruk ME, Leslie HH, Verguet S, et al. Quality of basic maternal care functions in health facilities of five African countries: an analysis of national health system surveys. *The Lancet Global health* 2016;4(11):e845-e55. doi: 10.1016/s2214-109x(16)30180-2 [published Online First: 2016/10/22]
17. The DHS Program. Service Provision Assessment (SPA) Overview.online: https://dhsprogram.com/What-We-Do/Survey-Types/SPA.cfm [accessed on 16 January 2020]
18. Moxon SG, Ruysen H, Kerber KJ, et al. Count every newborn; a measurement improvement roadmap for coverage data. *BMC pregnancy and childbirth* 2015;15 Suppl 2:S8. doi: 10.1186/1471-2393-15-s2-s8 [published Online First: 2015/09/24]
19. Bhattacharya AA, Allen E, Umar N, et al. Monitoring childbirth care in primary health facilities: a validity study in Gombe State, northeastern Nigeria. *Journal of global health* 2019;9(2):020411. doi: 10.7189/jogh.09.020411 [published Online First: 2019/07/31]
20. Gombe State Government. History of Gombe State, Nigeria. 2018;online: http://gombestate.gov.ng/history-2/ [accessed on 9 September 2019]
21. Oruonye E, Abubakar H, Ahmed M, et al. Hiv/Aids interventions in Gombe state Nigeria; challenges of sustaining the gains. *International Journal of Asian Social Science* 2017;7(6):448-57.
22. Izugbara CO, Wekesah FM, Adedini SA. Maternal Health in Nigeria. A Situation Update. Nairobi, Kenya: African Population and Health Research Centre, 2016.
23. National Bureau of Statistics and United Nations Children's Fund. Multiple Indicator Cluster Survey 2016–17, Survey Findings Report. Abuja: Nigeria National Bureau of Statistics and United Nations Children's Fund, 2017.
24. National Population Commission and ICF. Nigeria Demographic and Health Survey 2018. Key Indicators Report. Abuja, Nigeria, and Rockville, Maryland, USA, 2019.
25. Nigeria Health Management Information System. Gombe State facility deliveries, by facility type, January-December 2018. *Nigeria District Health Information System, version 2* 2019
26. Umar N, Quaife M, Exley J, et al. Toward improving respectful maternity care: a discrete choice experiment with rural women in northeast Nigeria. *BMJ global health* 2020;5(3):e002135. doi: 10.1136/bmjgh-2019-002135 [published Online First: 2020/03/24]
27. Bhattacharya AA, Umar N, Audu A, et al. Quality of routine facility data for monitoring priority maternal and newborn indicators in DHIS2: A case study from Gombe State, Nigeria. *PloS one* 2019;14(1):e0211265. doi: 10.1371/journal.pone.0211265 [published Online First: 2019/01/27]
28. Gombe State Ministry of Health. Gombe state framework for the implimentation of expanded access to family planning services. 2012;online: https://www.fhi360.org/sites/default/files/media/documents/nigeria-gombe-state-framework.pdf [accessed on 19 June 2020]
29. Gombe State Ministry of Health. Gombe State government strategic health development plan. 2010;online: https://drive.google.com/file/d/0B1DAmtM1BcbMeVBRUjNEakViXzQ/view [accessed on 19 June 2020]
30. Federal Ministry of Health. Task-shifting and task-sharing policy for essential health care services in Nigeria. Abuja, Nigeria, 2014.
31. Exley JL, Umar N, Moxon S, et al. Newborn resuscitation in Gombe State, northeastern Nigeria. *Journal of global health* 2018;8(2):020420. doi: 10.7189/jogh.08.020420 [published Online First: 2018/11/10]
32. Marchant T. Change in maternal and newborn health care. Interactions between families and frontline workers - their frequency, quality and equity - and coverage of

For peer review only - http://bmjopen.bmj.com/site/about/guidelines.xhtml

interventions of mothers and newborns. Report from Gombe State, Nigeria, 2012-2015. online: https://ideas.lshtm.ac.uk/wp-content/uploads/2017/08/Marchant_IDEAS_Gombe_State_Follow-upSurvey_2016.pdf [accessed on 9 September 2019]: IDEAS, London School of Hygiene & Tropical Medicine, 2016.
33. E4A-MamaYe. Gome PHC stockout of essential life-saving commodities scorecards. Jan - June 2018. 2018;online: https://mamaye.org/resources/scorecards/gombe-phc-stockout-essential-life-saving-commodities-scorecard [accessed on 20 June 2020]
34. IDEAS team. Informed Decisions for Actions in Maternal and Newborn Health 2010-17 Report: What works, why and how in maternal and newborn health. online: https://ideas.lshtm.ac.uk/wp-content/uploads/2017/09/LSHTM_IDEAS_PhaseIReport_Web-reduced.pdf [accessed on 9 August 2019]: IDEAS, London School of Hygiene & Tropical Medicine, 2017.
35. Makowiecka K, Marchant T, Betemariam W, et al. Characterising innovations in maternal and newborn health based on a common theory of change: lessons from developing and applying a characterisation framework in Nigeria, Ethiopia and India. *BMJ global health* 2019;4(4):e001405. doi: 10.1136/bmjgh-2019-001405 [published Online First: 2019/08/14]
36. Makowiecka K. The Pathway to Improved Maternal and Newborn Health Outcomes. Use of data for maternal and newborn health in Gombe State, Nigeria. online: https://ideas.lshtm.ac.uk/wp-content/uploads/2017/08/Makowiecka_IDEAS_Pathway_Improved_Maternal_Newborn_Health_Gombe_Nigeria_2016.pdf [accessed on 9 September 2019]: IDEAS, London School of Hygiene & Tropical Medicine, 2016.
37. Shobo OG, Umar N, Gana A, et al. Factors influencing the early initiation of breast feeding in public primary healthcare facilities in Northeast Nigeria: a mixed-method study. *BMJ open* 2020;10(4):e032835. doi: 10.1136/bmjopen-2019-032835
38. USAID. Maternal and Newborn Quality of Care Surveys. *Maternal and Child Health Integrated Program* 2013;online: https://www.mchip.net/qocsurveys/ [accessed on 10 September 2019]
39. Marx Delaney M, Maji P, Kalita T, et al. Improving Adherence to Essential Birth Practices Using the WHO Safe Childbirth Checklist With Peer Coaching: Experience From 60 Public Health Facilities in Uttar Pradesh, India. *Global Health: Science and Practice* 2017;5(2):217-31. doi: 10.9745/GHSP-D-16-00410
40. World Health Organization. Standards for improving quality of maternal and newborn care in health facilities. Geneva: World Health Organization, 2016.
41. World Health Organization. Consultation on improving measurement of the quality of maternal, newborn and child care in health facilities. Geneva: World Health Organization, 2013.
42. World Health Organization, United Nations Population Fund, UNICEF. Pregnancy, childbirth, postpartum and newborn care. A guide for essential practice (3rd edition). Geneva: World Health Organization, 2015.
43. Harvey SA, Blandon YC, McCaw-Binns A, et al. Are skilled birth attendants really skilled? A measurement method, some disturbing results and a potential way forward. *Bulletin of the World Health Organization* 2007;85(10):783-90. doi: 10.2471/blt.06.038455 [published Online First: 2007/11/27]
44. World Health Organization. WHO recommendations for the prevention and treatment of postpartum haemorrhage. Geneva: World Health Organization, 2012.
45. Semrau KEA, Hirschhorn LR, Marx Delaney M, et al. Outcomes of a Coaching-Based WHO Safe Childbirth Checklist Program in India. *The New England journal of medicine* 2017;377(24):2313-24. doi: 10.1056/NEJMoa1701075 [published Online First: 2017/12/14]
46. Munabi-Babigumira S, Glenton C, Lewin S, et al. Factors that influence the provision of intrapartum and postnatal care by skilled birth attendants in low- and middle-income

For peer review only - http://bmjopen.bmj.com/site/about/guidelines.xhtml

countries: a qualitative evidence synthesis. *Cochrane Database of Systematic Reviews* 2017(11) doi: 10.1002/14651858.CD011558.pub2
47. Vogel JP, Williams M, Gallos I, et al. WHO recommendations on uterotonics for postpartum haemorrhage prevention: what works, and which one? *BMJ global health* 2019;4(2):e001466. doi: 10.1136/bmjgh-2019-001466
48. Bartlett L, Cantor D, Lynam P, et al. Facility-based active management of the third stage of labour: assessment of quality in six countries in sub-Saharan Africa. *Bulletin of the World Health Organization* 2015;93(11):759-67. doi: 10.2471/blt.14.142604 [published Online First: 2015/11/10]
49. Moller AB, Newby H, Hanson C, et al. Measures matter: A scoping review of maternal and newborn indicators. *PloS one* 2018;13(10):e0204763. doi: 10.1371/journal.pone.0204763 [published Online First: 2018/10/10]
50. de Graft-Johnson J, Vesel L, Rosen HE, et al. Cross-sectional observational assessment of quality of newborn care immediately after birth in health facilities across six sub-Saharan African countries. *BMJ open* 2017;7(3):e014680. doi: 10.1136/bmjopen-2016-014680 [published Online First: 2017/03/30]
51. Rawlins B, Plotkin M, Rakotovao JP, et al. Screening and management of pre-eclampsia and eclampsia in antenatal and labor and delivery services: findings from cross-sectional observation studies in six sub-Saharan African countries. *BMC pregnancy and childbirth* 2018;18(1):346. doi: 10.1186/s12884-018-1972-1
52. Saxena M, Srivastava A, Dwivedi P, et al. Is quality of care during childbirth consistent from admission to discharge? A qualitative study of delivery care in Uttar Pradesh, India. *PloS one* 2018;13(9):e0204607. doi: 10.1371/journal.pone.0204607 [published Online First: 2018/09/28]
53. Hanson C, Munjanja S, Binagwaho A, et al. National policies and care provision in pregnancy and childbirth for twins in Eastern and Southern Africa: A mixed-methods multi-country study. *PLoS medicine* 2019;16(2):e1002749. doi: 10.1371/journal.pmed.1002749 [published Online First: 2019/02/20]
54. Marchant T, Beaumont E, Makowiecka K, et al. Coverage and equity of maternal and newborn health care in rural Nigeria, Ethiopia and India. *CMAJ : Canadian Medical Association journal = journal de l'Association medicale canadienne* 2019;191(43):E1179-e88. doi: 10.1503/cmaj.190219 [published Online First: 2019/10/30]
55. Federal Government of Nigeria. Second National Strategic Health Development Plan 2018-2022. 2019;online: http://www.health.gov.ng/doc/NSHDP%20II%20Final.pdf [accessed on 3 February 2020]
56. Network for Improving Quality of Care for Maternal NaCH. Quality of Care in Nigeria. 2019;online: http://www.qualityofcarenetwork.org/country-data/nigeria [accessed on 3 February 2020]
57. Leonard K, Masatu MC. Outpatient process quality evaluation and the Hawthorne Effect. *Social Science & Medicine* 2006;63(9):2330-40. doi: https://doi.org/10.1016/j.socscimed.2006.06.003
58. Leonard KL. Is patient satisfaction sensitive to changes in the quality of care? An exploitation of the Hawthorne effect. *Journal of Health Economics* 2008;27(2):444-59. doi: https://doi.org/10.1016/j.jhealeco.2007.07.004
59. Sharma G, Powell-Jackson T, Haldar K, et al. Quality of routine essential care during childbirth: clinical observations of uncomplicated births in Uttar Pradesh, India. *Bulletin of the World Health Organization* 2017;95(6):419-29. doi: 10.2471/blt.16.179291 [published Online First: 2017/06/13]
60. Hanson C, Schellenberg J. Redesigning maternal health services: is centralisation the answer in low-resource settings? *BMJ global health* 2019;4(3):e001488. doi: 10.1136/bmjgh-2019-001488 [published Online First: 2019/07/03]

For peer review only - http://bmjopen.bmj.com/site/about/guidelines.xhtml

Figure legends

Figure 1 Women and newborn's pathway from admission

Figure 2 History taking and initial examination

[FIGURE]

FIGURE NOTE: red line represents the average percent across all five time points. For problems experienced during previous pregnancies 10% of observers recorded that they did not know if the birth attendant asked the women. For HIV status 30% of observers recorded that they did not know if it had been checked

Figure 3 Examinations and procedures during first stage of labour

[FIGURE]

FIGURE NOTE: red line represents the average percent across all five time points

Figure 4 Care received during second and third stage of labour

[FIGURE]

FIGURE NOTE: red line represents the average percent across all five time points

Figure 5 Immediate newborn and postpartum care

[FIGURE]

FIGURE NOTE: red line represents the average across all five time points. Administers chlorhexidine and weighs baby was not included in the checklist in the first observation period (time point 1).

For peer review only - http://bmjopen.bmj.com/site/about/guidelines.xhtml

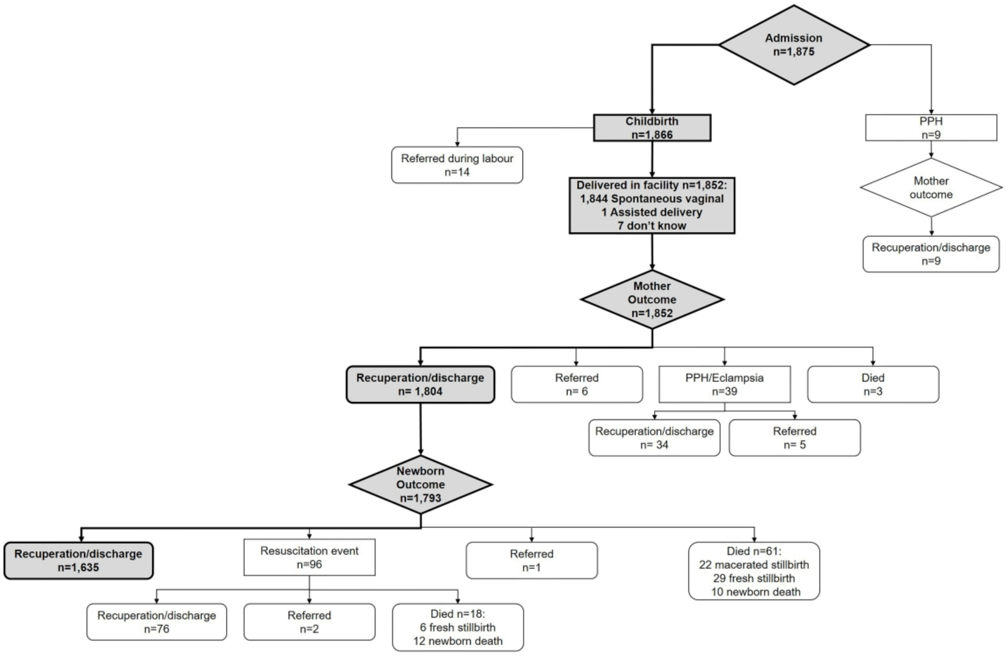

Figure 1 Women and newborn's pathway from admission

437x284mm (300 x 300 DPI)

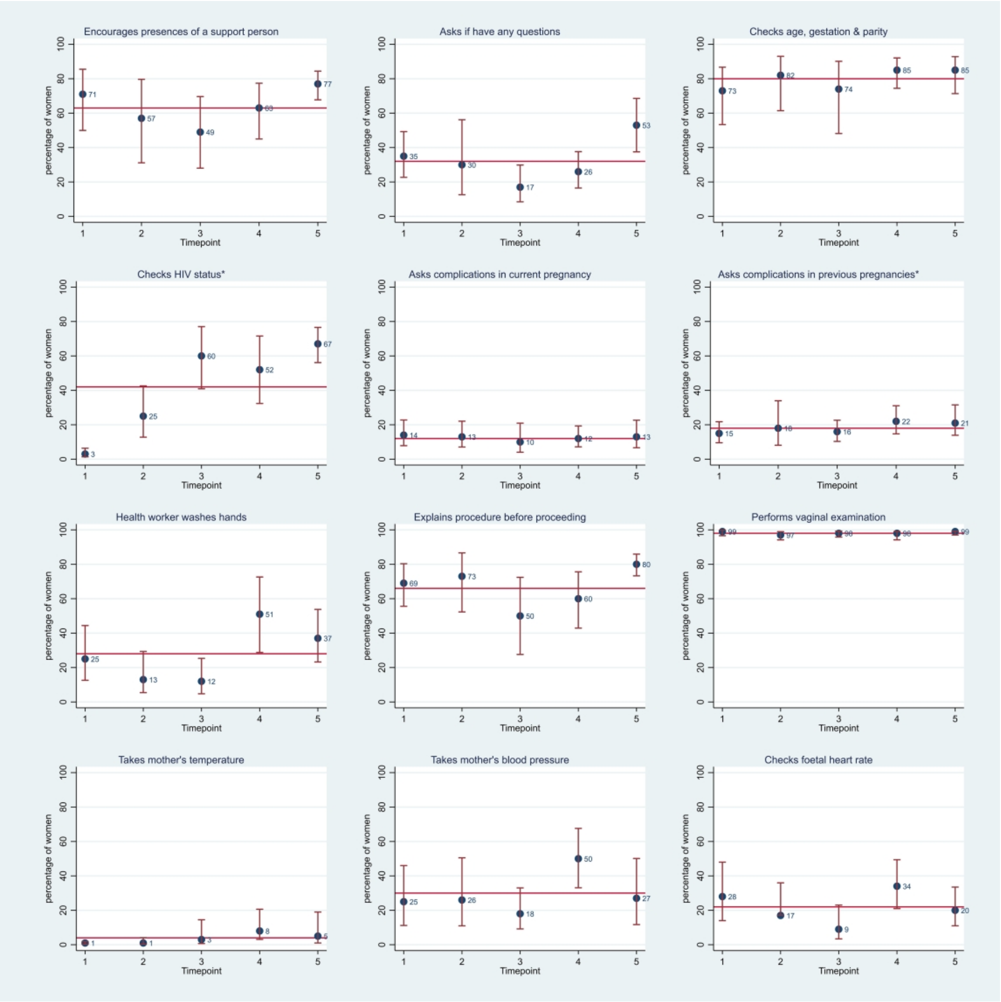

Figure 2 History taking and initial examination
[FIGURE]
FIGURE NOTE: red line represents the average percent across all five timepoints. For problems experienced during previous pregnancies 10% of observers recorded that they did not know if the birth attendant asked the women. For HIV status 30% of observers recorded that they did not know if it had been checked

360x360mm (300 x 300 DPI)

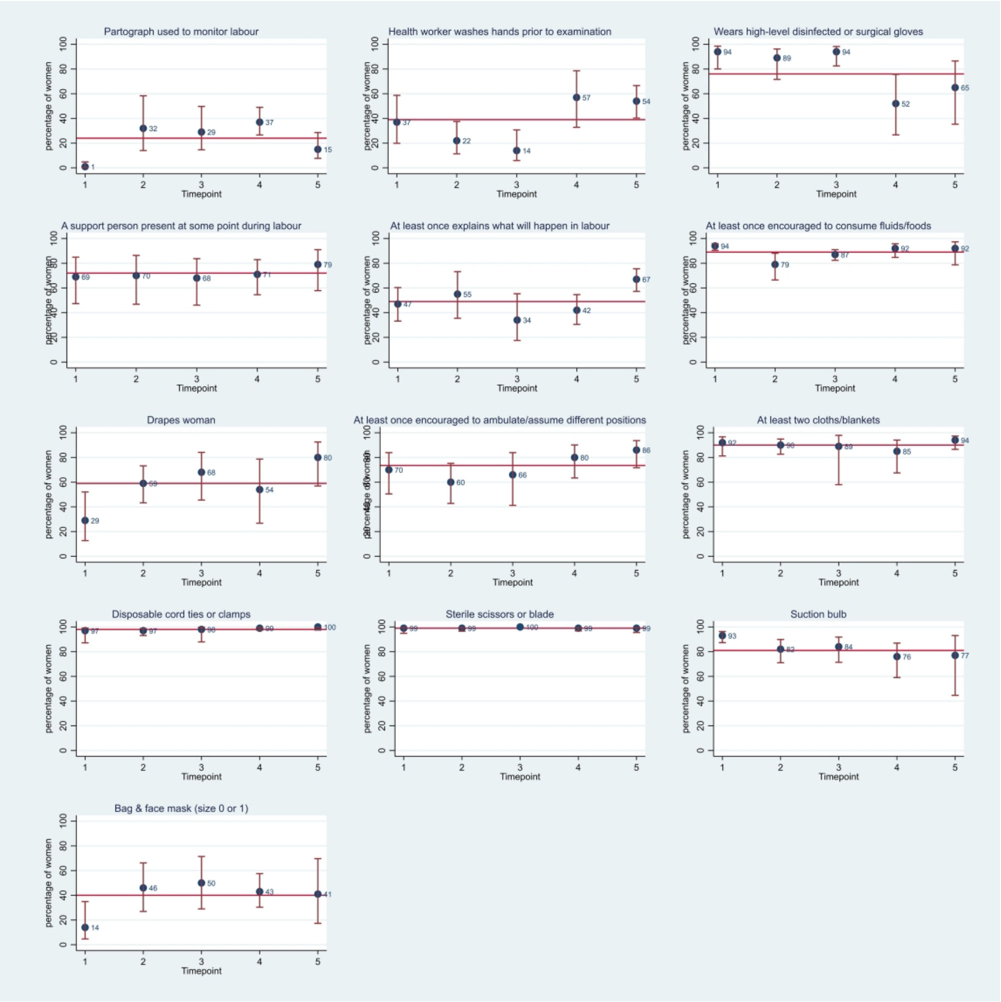

Figure 3 Examinations and procedures during first stage of labour

[FIGURE]

FIGURE NOTE: red line represents the average percent across all five timepoints

360x360mm (300 x 300 DPI)

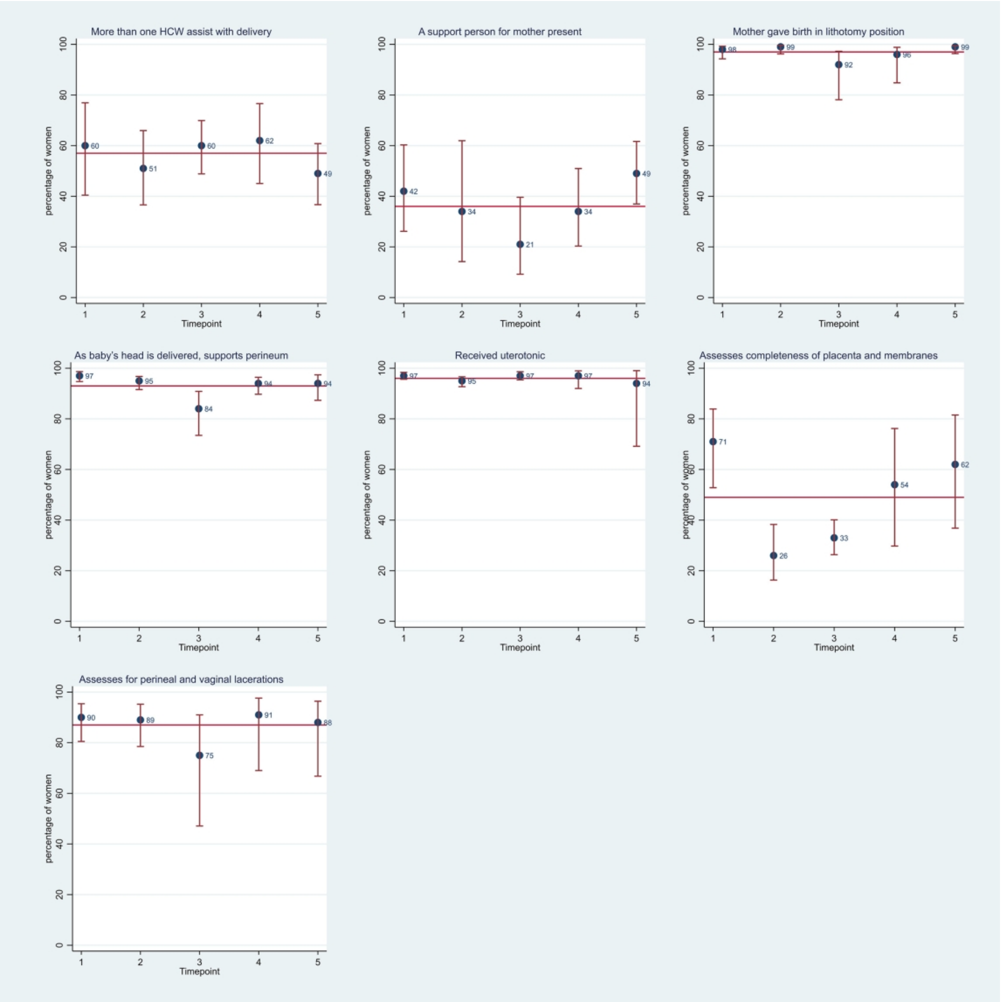

Figure 4 Care received during second and third stage of labour
[FIGURE]
FIGURE NOTE: red line represents the average percent across all five timepoints

240x240mm (300 x 300 DPI)

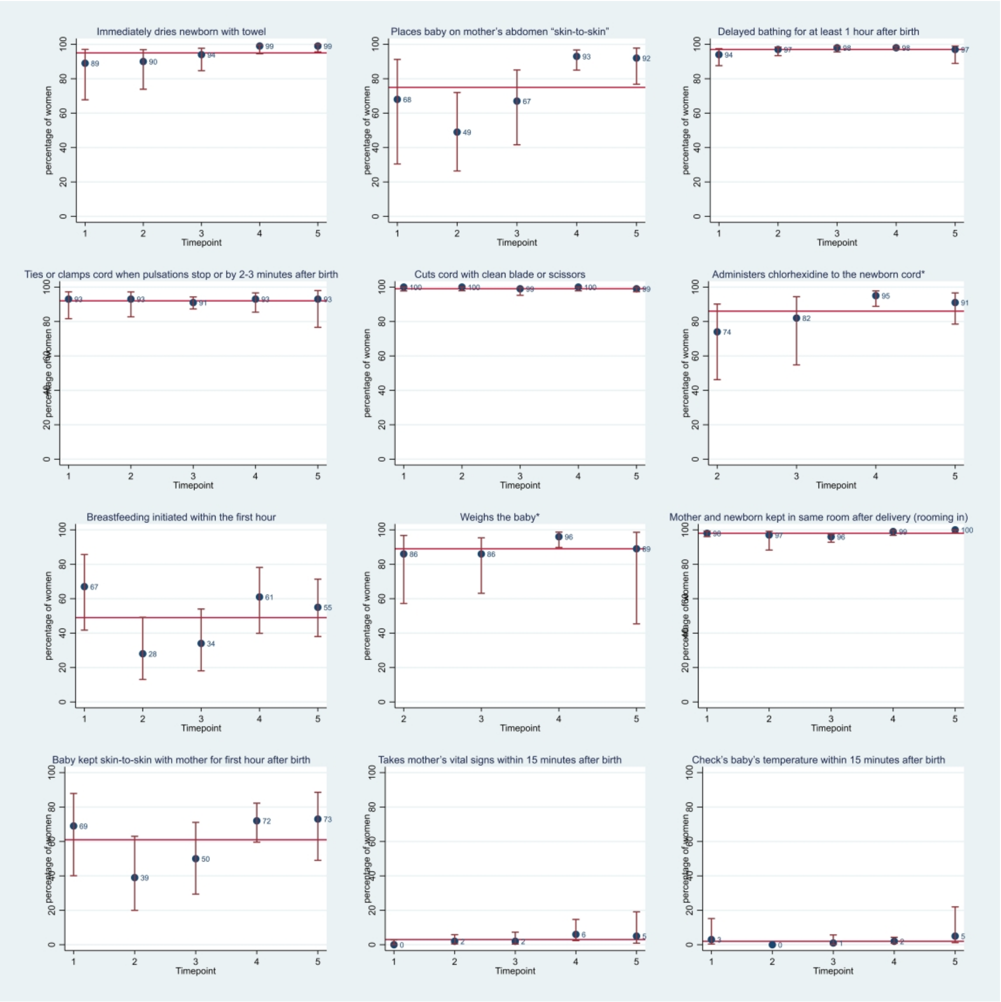

Figure 5 Immediate newborn and postpartum care
[FIGURE]
FIGURE NOTE: red line represents the average across all five timepoints. Administers chlorhexidine and weighs baby was not included in the checklist in the first observation period (timepoint 1).

Supplementary material: full table of results

	Observation period				
	1 (Aug 2016)	2 (Mar 2017)	3 (Aug 2017)	4 (Mar 2018)	5 (Aug 2018)
	Percentage of women (95%CI)				
**History taking & initial assessment**					
Encourages woman to have a support person	70.8% (50.0-85.5)	57.1% (31.1-79.7)	48.6% (28.0-69.6)	62.6% (45.0-77.4)	77.1% (67.8-84.4)
Asks if have any questions	34.8% (22.7-49.3)	30.0% (12.6-56.2)	16.6% (8.5-29.9)	25.7% (16.5-37.6)	53.4% (37.5-68.6)
Checks client card or asks client age, length of pregnancy and parity	73.2% (53.4-86.7)	82.3% (61.4-93.0)	74.4% (48.2-90.1)	85.3% (74.5-92.1)	85.0% (71.4-92.8)
Checks woman's HIV status	3.0% (1.4-6.3)	24.8% (12.8-42.6)	60.4% (40.9-77.0)	52.3% (32.4-71.6)	67.2% (56.1-76.6)
Asks whether experienced any complications during pregnancy	13.7% (7.9-22.8)	12.8% (7.1-22.1)	9.6% (4.0-20.9)	12.0% (7.2-19.3)	12.6% (6.6-22.7)
If had a previous pregnancy, whether experienced any complications during previous pregnancies	14.6% (9.5-21.8)	17.5% (8.1-34.0)	15.5% (10.3-22.6)	21.8% (14.7-31.0)	21.4% (13.9-31.6)
Health worker washes hands	25.3% (12.6-44.4)	13.4% (5.4-29.4)	11.5% (4.8-25.3)	50.9% (28.8-72.6)	37.2% (23.2-53.8)
Explains procedure to woman before proceeding	69.4% (55.6-80.3)	72.7% (52.3-86.6)	50.0% (27.6-72.4)	60.4% (42.9-75.6)	80.4% (73.3-85.9)
Performs vaginal examination	99.1% (96.6-99.8)	97.5% (94.2-998.9)	98.3% (95.8-99.3)	97.6% (94.2-99.0)	98.5% (97.0-99.3)
If performs vaginal exam, wears high-level disinfectant gloves	93.7% (82.8-97.9)	79.7% (48.6-94.2)	94.9% (84.3-98.5)	43.9% (21.3-69.3)	59.2% (35.9-79.0)
Takes temperature	0.6% (0.2-1.9)	1.1% (0.3-4.1)	3.4% (0.7-14.5)	8.3% (3.1-20.6)	4.7% (1.0-19.0)
Takes blood pressure	24.7% (11.2-46.0)	26.2% (11.0-50.5)	18.3% (9.2-33.0)	50.4% (33.1-67.6)	26.7% (11.7-50.0)
Checks foetal heart rate with fetoscope/Doppler/ultrasound	28.0% (14.0-48.0)	17.3% (7.2-36.0)	9.3% (3.4-23.0)	33.8% (21.0-49.4)	19.9% (11.0-33.5)
**First stage of labour: examination and procedures**					
Partograph used to monitor labour	0.6% (0.1-4.8)	32.3% (14.0-58.3)	29.1% (14.6-49.7)	37.1% (26.6-49.0)	15.4% (7.7-28.6)
Washes hands with soap & water or uses antiseptic prior to any examination of woman	37.2% (19.9-58.7)	21.7% (11.3-37.6)	14.3% (5.9-30.7)	57.2% (32.8-78.6)	53.7% (40.3-66.6)

For peer review only

Wears high-level disinfected or surgical gloves	94.2% (80.1-98.5)	88.9% (71.6-96.2)	94.2% (82.5-98.2)	51.6% (26.7-75.7)	65.2% (35.3-86.5)
Median number of vaginal examinations	2 (1-2)	1 (1-2)	2 (1-2)	1 (1-2)	1 (1-2)
Augments labour with oxytocin	14.5% (7.5-26.3)	12.6% (6.5-23.2)	24.9% (17.2-34.5)	18.0% (7.5-37.2)	15.4% (5.9-34.5)
Performs artificial rupture of membranes	7.6% (3.9-14.0)	9.6% (4.3-20.2)	9.0% (3.3-22.5)	3.5% (1.5-7.9)	1.8% (0.4-6.7)
A support person present at some point during labour	69.2% (47.3-84.9)	70.2% (46.8-86.3)	67.7% (46.1-83.7)	70.7% (54.5-82.9)	78.9% (57.8-91.0)
At least once explains what will happen in labour	46.5% (33.2-60.3)	55.1% (35.5-73.2)	33.9% (17.5-55.3)	42.1% (30.5-54.6)	67.0% (57.2-75.5)
At least encouraged to consume fluids/foods during labour	93.6% (90.4-95.8)	79.3% (66.5-88.1)	87.3% (82.4-91.0)	91.9% (84.7-95.8)	92.1% (78.7-97.3)
Drapes woman	28.5% (12.7-52.1)	59.1% (43.3-73.2)	67.7% (45.5-84.1)	53.7% (26.8-78.7)	80.2% (56.9-92.5)
At least once encourages woman to ambulate and assume different positions in labour	69.8% (50.5-83.9)	60.1% (42.8-75.2)	65.6% (41.2-83.9)	79.9% (63.4-90.1)	85.9% (71.7-93.6)
**First stage of labour: equipment and supplies laid out in preparation for delivery**					
At least two cloths/blankets	91.9% (81.2-96.7)	90.4% (82.7-94.9)	88.9% (58.0-97.9)	85.2% (67.5-94.1)	93.8% (86.6-97.3)
Disposable cord ties or clamps	96.5% (87.1-99.1)	97.0% (93.1-98.7)	98.4% (87.9-99.8)	99.3% (97.4-99.8)	99.6% (97.5-99.9)
Sterile scissors or blade	98.8% (94.9-99.8)	99.0% (96.6-99.7)	100%	98.9% (96.8-99.7)	98.7% (95.5-99.6)
Suction bulb	93.0% (87.3-96.3)	82.3% (71.0-89.9)	84.1% (71.4-91.8)	75.6% (59.1-87.0)	76.7% (44.6-93.0)
Bag & face mask (size 0 or 1)	14.0% (4.7-35.0)	46.0% (26.9-66.2)	50.3% (29.0-71.4)	43.5% (30.4-57.6)	41.0% (17.3-69.7)
**Second and third stage of labour**					
More than one HCW assist with delivery	60.1% (40.4-77.0)	51.4% (36.6-66.0)	59.8% (48.9-69.9)	62.1% (45.1-76.6)	48.7% (36.7-60.8)
A support person for mother present	42.3% (26.2-60.3)	34.2% (14.2-62.0)	20.5% (9.2-39.6)	34.0% (20.3-51.0)	49.3% (37.0-61.7)
Performs episiotomy	1.5% (0.4-5.4)	0.8% (0.2-4.4)	0.6% (0.1-2.4)	2.4% (1.0-5.8)	0.3% (0-2.6)
Mother gave birth in lithotomy position	97.9% (94.3-99.3)	98.9% (96.2-99.7)	91.9% (78.1-97.3)	95.6% (84.8-98.8)	98.5% (96.4-99.4)

For peer review only

As baby's head is delivered, supports perineum	97.3% (94.7-98.7)	94.7% (91.6-96.7)	84.0% (73.5-90.9)	93.9% (89.7-96.4)	94.1% (87.3-97.4)
Checks for another baby prior to giving the uterotonic	56.8% (31.7-78.9)	50.8% (33.6-67.9)	52.8% (37.9-67.2)	81.2% (59.5-92.7)	83.0% (67.9-91.9)
Received uterotonic	97.3% (95.6-98.4)	95.0% (92.7-96.6)	97.5% (95.4-98.6)	97.1% (92.0-99.0)	93.8% (69.2-99.0)
If uterotonic received, timing of administration					
At delivery	0.3% (0-2.4)	0.3% (0-2.7)	0.3% (0.1-1.3)	1.8% (0.6-5.0)	0
Within 1 minute of delivery	23.0% (9.4-46.4)	8.8% (2.7-25.0)	10.9% (5.8-19.5)	11.1% (4.9-23.1)	16.6% (9.0-28.6)
Within 3 minutes of delivery	28.5% (22.7-35.1)	30.7% (19.7-44.5)	26.1% (18.4-35.6)	37.0% (29.4-45.4)	51.3% (42.1-60.4)
More than 3 minutes	47.6% (31.6-64.1)	60.2% (41.1-76.7)	62.5% (54.0-70.3)	50.1% (37.9-62.4)	32.2% (25.3-39.9)
Applies traction to the cord while applying suprapubic counter traction	87.3% (80.8-91.8)	81.9% (61.9-92.7)	80.6% (70.5-87.9)	85.3% (67.3-94.3)	91.2% (71.7-97.7)
If skilled (doctor, nurse, midwife) birth assistant, applies traction to the cord while applying suprapubic counter traction	100%	95.4% (63.0-99.6)	88.7% (81.9-93.2)	82.4% (77.4-86.4)	90.5% (48.1-99.0)
If CHEW birth assistant, applies traction to the cord while applying suprapubic counter traction	87.6% (78.8-93.0)	80.9% (58.9-92.6)	81.9% (67.4-90.8)	85.7% (54.9-96.7)	88.9% (66.8-97.0)
If unskilled birth assistant, applies traction to the cord while applying suprapubic counter traction	86.1% (78.1-91.5)	77.8% (57.5-90.0)	77.0% (63.8-86.4)	85.2% (65.2-94.7)	93.0% (77.1-98.1)
Performs uterine massage immediately following the delivery of the placenta	73.7% (63.7-81.7)	78.6% (60.7-89.8)	63.8% (52.6-73.6)	79.0% (63.6-89.0)	77.4% (63.4-87.2)
Assesses completeness of placenta and membranes	70.7% (52.8-83.9)	25.8% (16.3-38.3)	32.9% (26.4-40.1)	53.8% (29.8-76.2)	61.6% (36.8-81.5)
Assesses for perineal and vaginal lacerations	90.2% (80.5-95.4)	89.4% (78.5-95.2)	75.0% (47.1-91.0)	90.5% (69.0-97.6)	88.0% (66.8-96.4)
**Immediate newborn and postpartum care**					
Immediately dries newborn with towel	89.3% (67.8-97.1)	90.5% (73.9-96.9)	93.9% (84.7-97.7)	98.7% (94.5-99.7)	99.4% (95.6-99.9)
Places baby on mother's abdomen "skin-to-skin"	68.1% (30.5-91.2)	49.0% (26.4-72.0)	66.9% (41.6-85.1)	92.8% (85.0-96.7)	92.3% (76.8-97.8)
Delayed bathing until at least 1 hour after birth	94.3% (87.6-97.5)	96.7% (93.4-98.4)	97.9% (95.6-99.0)	98.1% (96.9-99.0)	96.5% (88.9-99.0)

For peer review only

Ties or clamps cord when pulsations stop or by 2-3 minutes after birth	92.6% (81.6-97.3)	92.8% (82.7-97.2)	91.4% (87.3-94.3)	92.8% (85.4-96.6)	92.7% (76.6-98.0)
Cuts cord with clean blade or scissors	99.7% (97.8-100)	99.7% (97.9-100)	98.8% (95.2-99.7)	99.7% (97.8-100)	99.4% (97.3-99.9)
Administers chlorhexidine to the newborn cord	Not measured	73.7% (46.0-90.2)	81.9% (54.6-94.5)	95.0% (88.8-97.8)	91.1% (78.4-96.6)
Breastfeeding initiated within the first hour	67.5% (41.8-85.7)	27.6% (13.1-49.2)	33.7% (18.1-54.0)	60.6% (39.9-78.2)	55.3% (38.0-71.4)
Check's baby's temperature within 15 minutes after birth	2.7% (0.4-15.2)	0%	0.6% (0.1-5.7)	1.9% (0.8-4.3)	5.4% (1.2-22.0)
Takes mother's vital signs 15 minutes after birth	0.3% (0-2.8)	1.8% (0.5-5.9)	1.8% (0.4-7.3)	6.2% (2.4-14.7)	4.5% (0.9-19.1)
Weighs the baby	Not measured	86.3% (57.2-96.7)	85.6% (63.1-95.4)	96.3% (89.8-98.7)	88.5% (45.4-98.6)
Mother and newborn kept in same room after delivery (rooming in)	98.3% (96.1-99.3)	96.7% (88.3-99.1)	95.7% (92.8-97.5)	98.9% (96.8-99.7)	99.7% (98.4-98.8)
Baby kept skin-to-skin with mother for first hour after birth	68.8% (40.1-87.9)	39.5% (20.0-63.0)	50.3% (29.4-71.1)	72.3% (59.6-82.3)	73.2% (49.0-88.6)
Provides tetracycline ointment prophylaxis	0%	0.3% (0-2.9)	0.9% (0.2-4.6)	10.4% (4.4-22.4)	4.8% (1.5-13.9)
Administers Vitamin K to newborn	0%	0.3% (0-2.1)	0%	0%	0%
Administers chlorhexidine to the newborn cord	0%	73.7% (46.2-90.1)	81.9% (54.8-94.4)	95.0% (88.8-97.8)	91.1% (78.5-96.6)
If mother identified as HIV positive, administers ARV to newborn	100%	75.0% (20.5-97.2)	0%	0%	25.0% (2.3-82.5)
Administers antibiotics to mother	1.0% (0.3-3.6)	1.2% (0.5-2.7)	0%	1.8% (0.9-3.5)	6.0% (1.1-27.0)

For peer review only

Reporting checklist for cross sectional study.

Based on the STROBE cross sectional guidelines.

Instructions to authors

Complete this checklist by entering the page numbers from your manuscript where readers will find each of the items listed below.

Your article may not currently address all the items on the checklist. Please modify your text to include the missing information. If you are certain that an item does not apply, please write "n/a" and provide a short explanation.

Upload your completed checklist as an extra file when you submit to a journal.

In your methods section, say that you used the STROBE cross sectionalreporting guidelines, and cite them as:

von Elm E, Altman DG, Egger M, Pocock SJ, Gotzsche PC, Vandenbroucke JP. The Strengthening the Reporting of Observational Studies in Epidemiology (STROBE) Statement: guidelines for reporting observational studies.

		Reporting Item	Page Number
**Title and abstract**			
Title	#1a	Indicate the study's design with a commonly used term in the title or the abstract	1

Abstract	#1b	Provide in the abstract an informative and balanced summary of what was done and what was found	2
**Introduction**			
Background / rationale	#2	Explain the scientific background and rationale for the investigation being reported	4
Objectives	#3	State specific objectives, including any prespecified hypotheses	4
**Methods**			
Study design	#4	Present key elements of study design early in the paper	4
Setting	#5	Describe the setting, locations, and relevant dates, including periods of recruitment, exposure, follow-up, and data collection	4
Eligibility criteria	#6a	Give the eligibility criteria, and the sources and methods of selection of participants.	5-7
	#7	Clearly define all outcomes, exposures, predictors, potential confounders, and effect modifiers. Give diagnostic criteria, if applicable	6-7
Data sources / measurement	#8	For each variable of interest give sources of data and details of methods of assessment (measurement). Describe comparability of assessment methods if there is more than one group. Give information separately for for exposed and unexposed groups if applicable.	5

For peer review only

Bias	#9	Describe any efforts to address potential sources of bias	n/a
Study size	#10	Explain how the study size was arrived at	5
Quantitative variables	#11	Explain how quantitative variables were handled in the analyses. If applicable, describe which groupings were chosen, and why	7
Statistical methods	#12a	Describe all statistical methods, including those used to control for confounding	7
Statistical methods	#12b	Describe any methods used to examine subgroups and interactions	n/a
Statistical methods	#12c	Explain how missing data were addressed	n/a
Statistical methods	#12d	If applicable, describe analytical methods taking account of sampling strategy	7
Statistical methods	#12e	Describe any sensitivity analyses	n/a
**Results**			
Participants	#13a	Report numbers of individuals at each stage of study—eg numbers potentially eligible, examined for eligibility, confirmed eligible, included in the study, completing follow-up, and analysed. Give information separately for for exposed and unexposed groups if applicable.	8-10
Participants	#13b	Give reasons for non-participation at each stage	5

For peer review only

Participants	#13c	Consider use of a flow diagram	8
Descriptive data	#14a	Give characteristics of study participants (eg demographic, clinical, social) and information on exposures and potential confounders. Give information separately for exposed and unexposed groups if applicable.	8
Descriptive data	#14b	Indicate number of participants with missing data for each variable of interest	9-10
Outcome data	#15	Report numbers of outcome events or summary measures. Give information separately for exposed and unexposed groups if applicable.	10-12
Main results	#16a	Give unadjusted estimates and, if applicable, confounder-adjusted estimates and their precision (eg, 95% confidence interval). Make clear which confounders were adjusted for and why they were included	10-12
Main results	#16b	Report category boundaries when continuous variables were categorized	n/a
Main results	#16c	If relevant, consider translating estimates of relative risk into absolute risk for a meaningful time period	n/a
Other analyses	#17	Report other analyses done—e.g., analyses of subgroups and interactions, and sensitivity analyses	12
**Discussion**			
Key results	#18	Summarise key results with reference to study objectives	12-13

For peer review only

Limitations	#19	Discuss limitations of the study, taking into account sources of potential bias or imprecision. Discuss both direction and magnitude of any potential bias.	14
Interpretation	#20	Give a cautious overall interpretation considering objectives, limitations, multiplicity of analyses, results from similar studies, and other relevant evidence.	14-15
Generalisability	#21	Discuss the generalisability (external validity) of the study results	14
**Other Information**			
Funding	#22	Give the source of funding and the role of the funders for the present study and, if applicable, for the original study on which the present article is based	16

None The STROBE checklist is distributed under the terms of the Creative Commons Attribution License CC-BY. This checklist can be completed online using https://www.goodreports.org/, a tool made by the EQUATOR Network in collaboration with Penelope.ai

For peer review only
